# Development of a Python-based electron ionization mass spectrometry amino acid and peptide fragment prediction model

**DOI:** 10.1371/journal.pone.0297752

**Published:** 2024-02-16

**Authors:** Dominic N. McBrayer, Christina Signoretti, Matthew Pesce, Brianna M. Flood, Sneha Varghese, Fares Sirdah, Elena Toscano, Irtiza Bhatti, Shahadat Hossain

**Affiliations:** Department of Chemistry, State University of New York, New Paltz, New Paltz, NY, United States of America; Victoria University of Wellington, NEW ZEALAND

## Abstract

The increased fragmentation caused by harsher ionization methods used during mass spectrometry such as electron ionization can make interpreting the mass spectra of peptides difficult. Therefore, the development of tools to aid in this spectral analysis is important in utilizing these harsher ionization methods to study peptides, as these tools may be more accessible to some researchers. We have compiled fragmentation mechanisms described in the literature, confirmed them experimentally, and used them to create a Python-based fragment prediction model for peptides analyzed under direct exposure probe electron ionization mass spectrometry. This initial model has been tested using single amino acids as well as targeted libraries of short peptides. It was found that the model does well in predicting fragments of peptides composed of amino acids for which the model is well-defined, but several cases where additional mechanistic information needs to be incorporated have been identified.

## Introduction

Electron ionization mass spectrometry (EI-MS) uses a high-energy electron beam to ionize the sample, often resulting in the destruction of the structure of the sample molecules into fragments. This tendency to break sample molecules into smaller ions can sometimes be helpful by providing structural information. However, in the case of peptides, fragmentation can make confirming the identity of a peptide a challenging task because of the polymeric nature of peptides and the tendency of many of their smaller fragments to converge to the same or similar mass over charge (*m/z*) values. Despite this, some efforts have been made to study short peptides and amino acids with EI-mass spectrometry (EI-MS), usually using functionalized forms of the peptides/amino acids to make them volatile enough to be run through a gas chromatography column prior to ionization by the electron beam [1–3]. This functionalization is often required because of the amphoteric properties of many of the amino acids composing peptides, which reduces their volatility due to in-solution ionization. Direct exposure probe EI-MS (DEP-EI-MS), however, allows for the detection of un-functionalized amino acids by driving them directly into the gas phase and into the path of the electron beam. The development of softer ionization methods for mass spectrometry (e.g., electrospray ionization (ESI) or matrix-assisted laser desorption ionization (MALDI)) has provided a helpful means of studying peptides, however, such instrumentation, although becoming more prevalent, remains unavailable at some institutions while EI-MS instruments are more widely accessible.

Efforts to predict EI-MS spectra using calculations or machine learning are currently somewhat limited by available training data sets and/or computational power, which can restrict the size of the predicted molecule. Currently, the size restrictions appear to make EI-MS spectrometry less useful for identifying peptides, although efforts are being made to use machine learning to develop predictive models based on individual amino acids [4,5].

**Fig 1:**
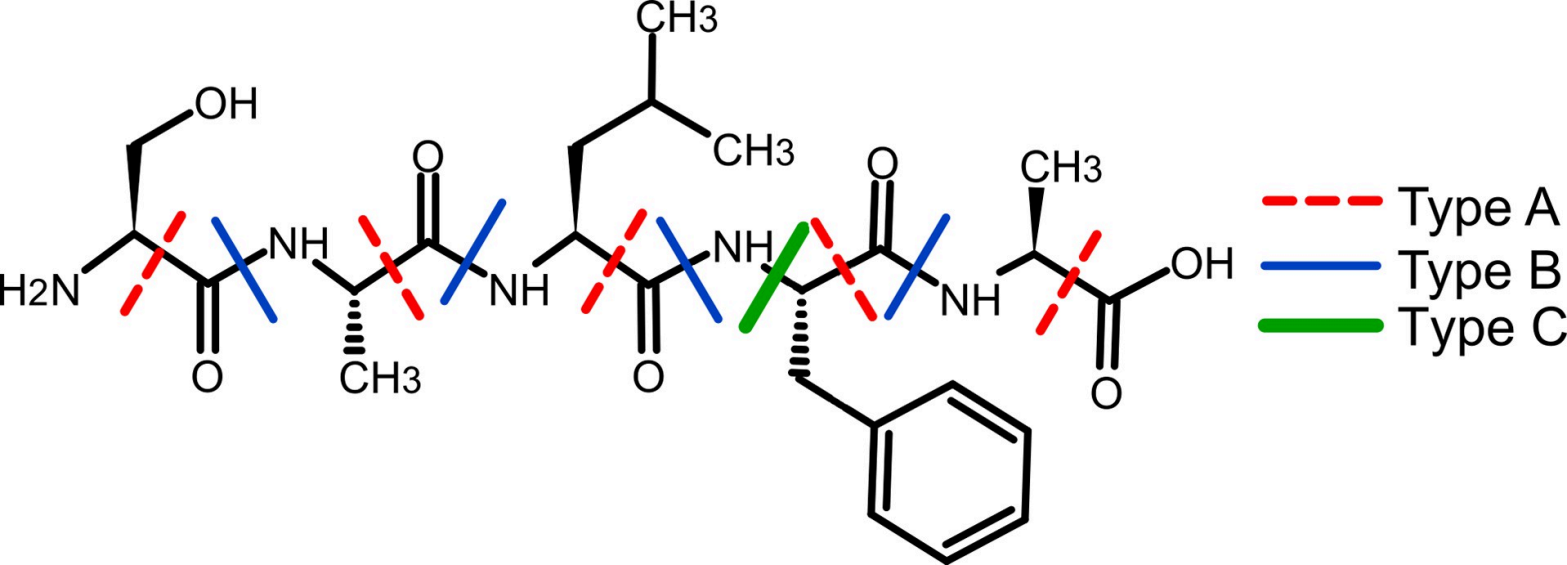
Classes of backbone cleavage on peptides. An example peptide, SALFA, demonstrating the locations of the three types of backbone cleavage. Type A cleavage (red dashed lines) occurs between the α-carbon and the carbonyl carbon of the same amino acid. Type B cleavage (blue narrow lines) occurs at the peptide bond between two amino acids. Type C cleavage (green thick lines) occurs between the α-carbon and the nitrogen of the same amino acid and it results in the release of the aromatic side chain. Type C has only been observed in the aromatic residues of Phe, Trp, and Tyr [3].

Some work has been done previously by others conducting EI-MS analyses on peptides. For example, around 200 of the 400 possible dipeptides (using proteinogenic amino acids) were studied using gas chromatography EI-MS (GC-EI-MS) [3]. This study identified several fragmentation mechanisms including type A, B, and C cleavage (Fig 1) of the peptide backbone as well as some common fragmentation pathways for some of the aliphatic amino acids (Val, Leu, Ile) and for the aromatic amino acids (Tyr, Phe). It also found that β-elimination was common for the alcohol-containing amino acids (Ser, Thr) and identified three possible fragments that carboxylic acid groups could undergo, including the C-terminus of all amino acids as well as the side chains of Glu and Asp. Due to issues with their functionalization process, the authors did not identify any fragmentations common to the amide-containing amino acids Asn or Gln. Other studies have also identified fragmentation products of peptides and amino acids in an electron beam, although mechanisms have not always been proposed for how those fragments formed [6–10].

We have conducted experiments on all of the single, unfunctionalized proteinogenic amino acids and a select subset of unfunctionalized di-, tri-, and tetrapeptides using DEP-EI-MS. The experimental focus was on confirming mechanisms already reported by others and identifying additional, generalizable mechanisms in order to characterize the mechanisms for use in the creation of a data-driven combinatorial fragment prediction model. The goal is for this model to aid in the confirmation of the presence of the desired peptide in the mass spectrum of a crude synthetic aliquot.

To this end, a data scheme was adopted using 1 x 6 arrays (Python lists) to represent the chemical formulas (e.g., the numbers of C, H, N, O, S, as well as the net charge) of source peptides and their predicted fragments. Lists are native python objects that store a sequence of other objects in the specified order, allowing them to represent 1 x n arrays of numbers where n is the number of entries in the list. For example, a fragment’s formula can be represented in Python by the following list [C,H,N,O,S,z] where C, H, N, O, S, and z are the numbers of the associated elements and the net charge respectively. Although in DEP-EI-MS, only singly charged species are expected, we included the net charge field in our formulas to allow for expansion of the model to support larger charges in the event that functionality might be needed in the future. A program was then written in Python to apply the fragment prediction model to systematically combine confirmed fragmentation mechanisms to generate a predicted “fingerprint” of possible spectrum peak *m/z* values. Several “flag” words were included in the definition text file to distinguish between cases where the observed fragment is nonrelative (e.g. will always have the same m/z value regardless of the parent compound’s structure and can therefore never be used combinatorially) and thus represents a fragment that will not provide any further fragmentation data in the model rather than relative fragments that instead result in variable end *m/z* values based on the composition of the parent structure, and which can a produce a fragment that may be capable of further fragmentation.

## Results and discussion

### Reproducible fragmentation products identified/confirmed from the proteinogenic amino acids

In this study, we have confirmed several fragment events and/or mechanisms that have previously been reported under different conditions [3,6,11] also occur during DEP-EI-MS (Tables 1 and 2). We have categorized these events and/or mechanisms by amino acid functional group as part of using them in our combinatorial fragmentation model. Table 1 contains reproducible DEP-EI-MS fragment events that give relative mass changes according to their suspected mechanisms. In this case, relative mass changes mean that the resulting detected fragment’s *m/z* will vary depending on the structure of the original molecule (e.g., the original molecule’s mass is being changed and detected and so the new *m/z* value resulting from the fragmentation event will be relative to the starting peptide’s mass). Table 2 contains identified reproducible DEP-EI-MS fragment events that are non-relative. The *m/z* value observed from a given non-relative fragmentation event corresponds to the lost fragment itself, so the *m/z* value from that event will not change with respect to the sequence of the peptide. These fragmentation types cannot occur in combination with other fragmentation events in the same way that relative fragmentations can because they correspond to a component lost from the main peptide backbone, rather than to the remaining peptide backbone after a fragmentation event. Therefore, the information provided from non-relative fragmentation is limited to describing the presence of the associated amino acid. The *m/z* value observed from a given relative fragmentation event, however, will vary with the mass of the parent peptide/fragment; thus, it can provide insights into other aspects of the parent peptide’s structure such as parts of its sequence.

**Table 1:** Relative mass fragmentation events^a^.

Fragmentation Mechanism	Apparent Associated Atom Loss(es)	Approximate Associated *m/z* Change^b^	Associated Amino Acids	Observed in Other Studies^c^
Ammonia	NH_3_	M—17	N, Q	[11]
β-Elimination	OH_2_ or SH_2_	M—18 or M—34	T, S, C	[3]
Carboxylic Acid (Whole)	CO_2_H	M—45	D, E, C-Term	[3]
Carboxylic Acid (Partial Radical)	OH	M—17	D, E, C-Term	[3]
Carboxylic Acid (Partial)	OH_2_	M—18	D, E, C-Term	[3]
Partial Side Chain Loss	C_2_H_5_S	M—61	M	[11]
Lost Side Chain	Variable (see ~74 *m/z* for single amino acid)	Variable	C, D, E, F, H, I, K, L, M, N, Q, R, S, T, V, W	[11]
Side Chain Cyclization	NH_3_	M—17	K	[11]
*C*-terminus + alkene ^d^	CO_2_H_2_	M—46	K, P, I	[11]
Side Partial Guanidino Loss	CH_2_N_2_	M—42	R	
Side Guanidino Loss + Alkene	CH_6_N_3_	M—60	R	

^a^ Mechanisms are based on monoisotopic mass change comparisons found with derivatized or non-derivatized amino acids/peptides (all have been confirmed in this study to occur in non-derivatized amino acids/peptides). All predictions are for non-derivatized amino acids or peptides.

^b^M is the peptide/fragment mass prior to further fragmentation.

^c^Mechanism was seen for at least one amino acid in the given study.

^d^ Since loss of the C-terminus has been observed to be combinatorial, alkene formation was treated as a separate mechanism in the model, although it has only been observed thus far in combination with some form of *C*-terminal loss.

**Table 2:** Non-relative mass fragmentation events (charged side chain losses)^a^.

Fragmentation Mechanism	Associated Amino Acids	Apparent Associated Formulas	Associated *m/z*	Observed in Other Studies^b^
Charged Side Chain Loss	F	C_7_H_7_^+^	91.1	[3,11]
	Y	C_7_H_7_O^+^	107.0	[3,11]
	W	C_9_H_8_N^+^	130.1	[11]
	H	C_4_H_5_N_2_^+^	81.0	[3,11]
	M	C_3_H_7_S^+^	75.0	
	L	C_4_H_9_^+^	57.1	
Charged Side Chain Loss (Alkene)	F	C_8_H_7_^+^	103.1	[11]
	Y	C_8_H_7_O^+^	119.0	[11]
	H	C_5_H_5_N_2_^+^	93.1	[11]
Charged Side Chain Loss (Radical)	H	C_4_H_6_N_2_^+^	82.1	[11]
Charged Side Chain Methylene Sub Frag Loss	M	C_2_H_5_S^+^	61.0	[11]

^a^ Mechanisms are based on monoisotopic mass change comparisons found with derivatized or non-derivatized amino acids/peptides (all have been confirmed in this study to occur in non-derivatized amino acids/peptides). All predictions are for non-derivatized amino acids or peptides.

^b^Mechanism was seen for at least one amino acid in the given study.

**Fig 2:**
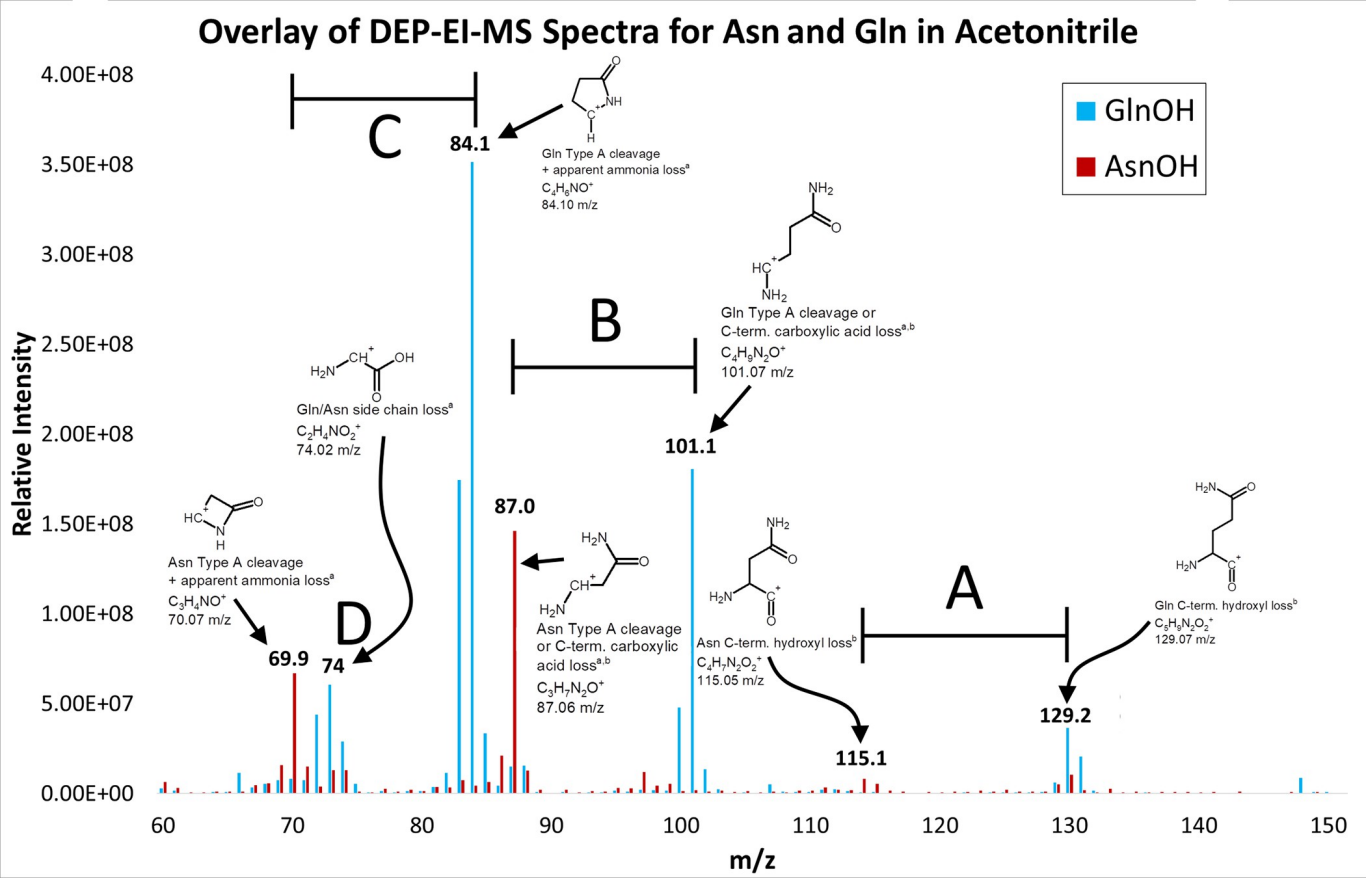
Comparison of reproducible Gln and Asn fragmentation behavior during DEP-EI-MS. Three dominant fragmentation mechanisms were observed in the Gln (blue trace) and Asn (red trace) mass spectra. A) Peaks associated with an “Ammonia loss” fragmentation are specific to the amide-containing amino acids. Based on the relative intensity, having this be the sole fragmentation event is relatively rare. B) Peaks associated with loss of the *C*-terminal carboxy group is a common fragmentation event seen in most amino acids and peptides tested. C) Peaks associated with the combination of both a loss of the *C*-terminal carboxy group and the “Ammonium loss” fragmentation event. This is particularly favored in Gln. D) Peaks at around 74 *m/z* which corresponds with the loss of side chain and is held in common with any amino acid side chain loss. Once again, this fragmentation peak is more pronounced in Gln. The maximum peak intensity is 2.86 X 10^8^ counts for Gln, and 1.38 X 10^8^ counts for Asn. Proposed structures are shown along with the resulting fragment formula and monoisotopic *m/z*. ^a^degradation type also observed in [11], ^b^degradation type also observed in [3].

As can be seen in Table 1 above, one fragment degradation mechanism held in common during DEP-EI-MS across nearly all amino acids is the loss of the side chain with a positive charge left on the amino acid backbone. Since in this form of fragmentation, the backbone is all that defines the differences between the amino acid fragments, the resulting peak can likely only be used for determining that amino acids may be present in the sample, but not the identity of those amino acids. However, in the context of a peptide, it is possible that different combinations of these side chain losses on the same peptide may aid in verifying a suspected peptide’s contents and possibly even its sequence. Several of the identified mechanisms have also been observed to occur in a combinatorial manner as we initially hypothesized. For example, the apparent loss of ammonia from Asn and Gln occurs individually, but also in combination with *C*-terminus fragmentation events within the same amino acid (Fig 2). In Gln, the combined loss of the *C*-terminal carboxyl group along with side chain ammonia loss was the most abundant degradation mass observed while in Asn the loss of the *C*-terminal carboxyl group alone dominated based on relative peak intensities.

**Fig 3:**
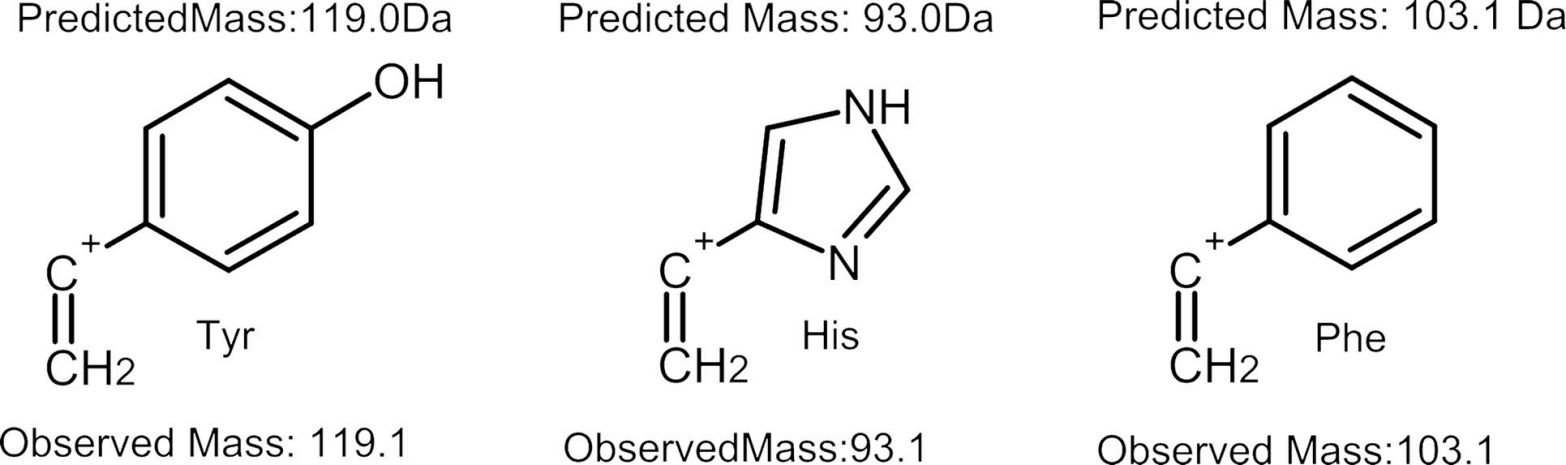
Observed cationic alkene side chain fragments from aromatic residues. For three of the aromatic amino acids, a peak was observed which corresponds to the loss of the side chain with a gain of charge along with the formation of a double bond between the α-carbon and the first carbon of the side chain. The above structures, each displaying extended conjugation from the aromatic side chain to the α-carbon, represent the likely fragment produced.

A very common degradation mode seen in aromatic residues was detection of the detached side chain either with (Phe, Tyr, His) or without (Phe, Tyr, His, Trp) the apparent formation of an alkene (Fig 3, S21–S24 Figs). These degradation products have also been observed under different EI-MS conditions [11]. Charged side chains were also reproducibly observed for a few aliphatic amino acids (Met and Leu, S25 and S26 Figs). In the case of Met, this included loss of either the entire side chain as a charged fragment or a partial loss (less one methylene) of the sidechain as a charged fragment.

After observing many of the β-elimination peaks that others had reported for Ser and Thr (S27 and S28 Figs) [3], we hypothesized that we would see similar behavior for Cys (S29 Fig). This proved to be the case, with Cys fragments suggesting fragmentation events similar to those seen with Ser and Thr. Comparing relative peak intensities within the spectra suggested the most abundant degradation product of Ser was the loss of its side chain while in Thr and Cys it was the loss of the *C*-terminal carboxyl.

We reproducibly detected all three of the previously reported *C*-terminus fragment types [3]. Based on the relative peak intensities within the spectra, the complete loss of the *C*-terminal carboxylic acid appears to be the most prevalent form of *C*-terminal fragmentation, while hydroxyl radical loss or water loss rarely occur in comparison. We observed combinatorial degradation behaviors between the carboxylic acids in Glu and Asp (S30 and S31 Figs) alongside their *C*-terminal carboxylic acids. In these cases, mixtures of events including hydroxyl radical loss, water loss, and/or carboxyl radical loss were observed. Combinatorial degradation with hydroxyl radical loss gave peaks with much lower relative intensity. For both Glu and Asp, the loss of both a water and a carboxyl group between the side chain and the *C*-terminus appeared to be the most common fragmentation mode based on the relative intensities of the peaks.

The basic residues Lys and Arg (S32 and S33 Figs) exhibited more unique fragmentation behavior than many of the amino acids. Lys reproducibly produced a fragment peak that resembled the apparent ammonia loss mechanism observed in Asn and Gln. This fragmentation has been observed before and has been attributed to the cyclization of Lys resulting in loss of its side chain amine [11]. Based on relative peak intensities, this mechanism in combination with loss of the *C*-terminal carboxyl group appears to result in the most abundant fragmentation products of Lys. The Arg fragments resembled a mixture of behavior of the aliphatic side chains and the aromatic groups with degradation producing charged side chain fragments. These fragments appeared to be localized around the guanidino group, producing *m/z* values that matched best with loss of the guanidino group itself or loss of the guanidino group along with some of the aliphatic portions of the side chain. Comparing relative intensities suggests that the most common fragmentation pattern for Arg is the loss of the guanidino group with formation of an alkene alongside the loss of the *C*-terminal carboxyl group.

Lys, Pro (S34 Fig), and Ile (S35 Fig) each showed a fragment product that appeared to correspond with the loss of the *C*-terminal carboxyl group combined with the formation of an alkene. The spectra for Lys and Pro contained low intensity peaks corresponding to alkene formation with hydroxyl radical loss, while the spectrum for Ile only contained alkene formation combined with *C*-terminal carboxyl group loss. The most prevalent Val (S36 Fig) fragments involved full or partial loss of the side chain or C-terminal degradations.

### Summary of the Python-based prediction model’s approach

**Fig 4:**
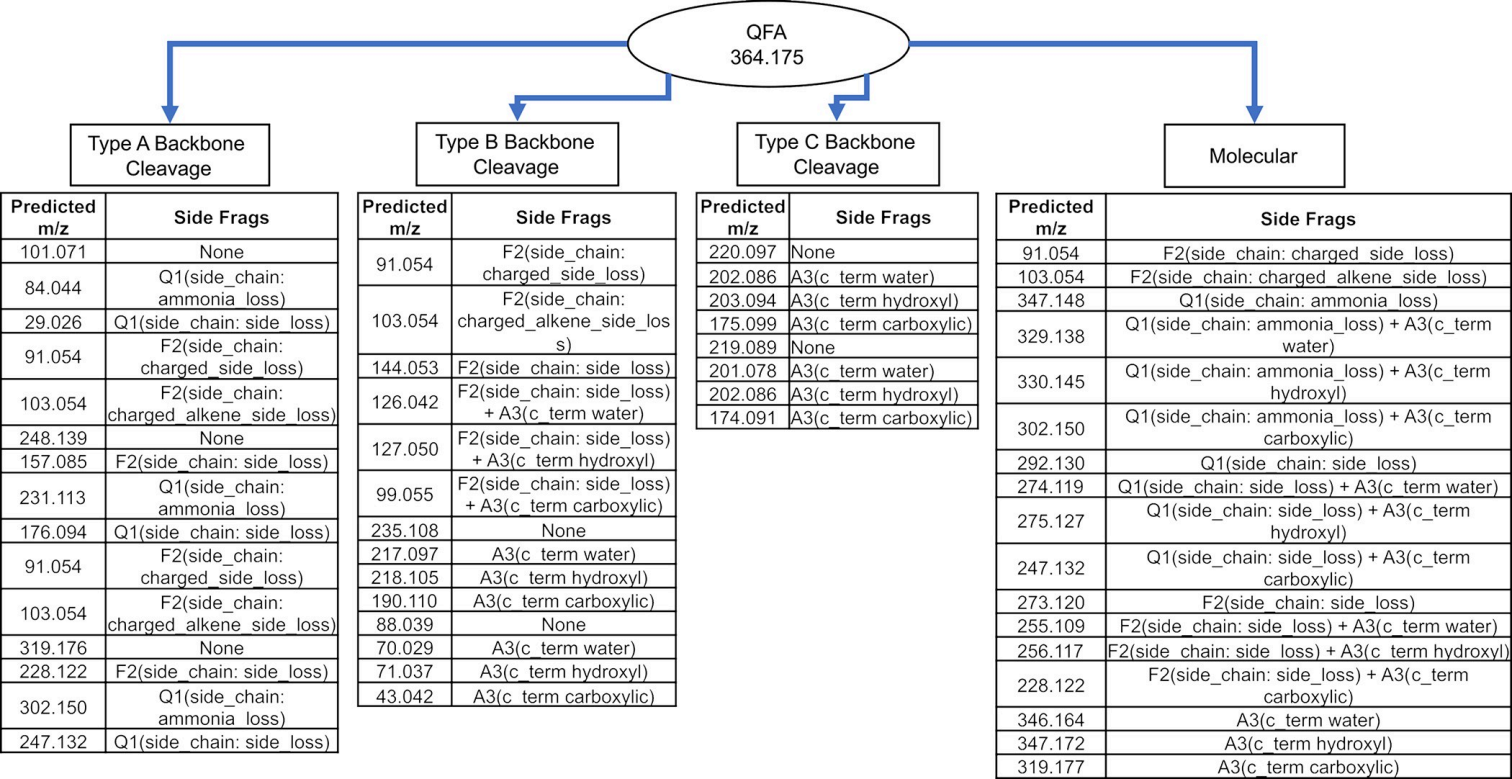
QFA fragment peak generation flow chart. The current model produces 55 fragments including the original peptide with unique mechanism flow paths after duplicates are removed. Note that the presence of non-relative fragmentation events does result in a few duplicated *m/z* predictions because those events are not combinatorial with the fragmentations that might have preceded them in a given degradation pathway since they will result in the same end fragment.

It was hypothesized that at least some, if not all fragmentation events had the capacity to be combinatorial in nature. For example, a backbone fragmentation event might also be accompanied by one or more side chain fragmentations within the resulting fragment. Therefore, the model was designed to allow for a “mix and match” approach when making predictions in order to create an exhaustive list of possible fragments. To keep track of the theoretical fragmentation paths for the generated fragments, each fragment maintains a record of the fragmentation mechanisms applied that resulted in its generation (see Methods for more details on the model design approach). Fig 4 depicts an example prediction flow chart for the sequence QFA. Because the program is essentially generating predictions by systematically applying defined fragmentation mechanisms with the associated “book-keeping”, it is a deterministic process that will always generate the same results for a given set of mechanism definitions. The source code and definition files containing the fragmentation mechanism data are publicly available on Github (See S2 File for a link).

### Comparison of current model predictions with short peptides

A total of 12 tri/tetra peptides were synthesized (Table 3) using Wang resin preloaded with alanine. In addition, 9 dipeptides were synthesized (S1 Table) using Wang resin preloaded with alanine or isoleucine. This meant all syntheses required a maximum of three coupling reactions (for the tetrapeptide). To simulate a common usage in a lab environment, crude samples of the peptides were tested to see how well the model would match up in the event of trying to confirm the presence of a desired synthetic peptide and to gain insights into areas where the model needs additional improvement to allow it to handle predictions in crude samples. Samples containing representative compounds for the protecting groups in the synthesized peptides were also analyzed to aid with identifying peaks arising from those groups. Table 4 lists the major observed peaks associated with the protecting groups used.

**Table 3:** Crude synthetic tri/tetra peptides used for prediction comparison.

Peptide Sequence	M^+^ Ion *m/z*
AFA	307.15
CIA	305.14
QFA	364.17
WFA	422.19
WHA	412.19
KMA	348.18
MKA	348.18
SHA	313.14
ARA	316.19
EEA	347.13
QKA	345.20
SEQA	433.18

**Table 4:** Major peaks arising from protecting groups used in peptide synthesis.

Protecting Group Standard	Observed peaks/peak clusters (*m/z*)
Fmoc-Cl	88, 89, 139, 151, 152, 163, 164, 165, 166, 176, 177, 178, 179, 196, 258, 260
Trt-Cl	77, 78, 105, 129, 154, 155 165, 166, 167, 181, 182, 183, 184, 243, 244
Pbf-Cl	91, 100, 115, 119, 129, 147, 173, 174, 175, 176, 189, 190
Boc/Ot-Bu (di-tert-butyl carbamate)	69, 70, 71, 82, 83, 84, 100, 111, 112, 113, 129, 147, 157

**Fig 5:**
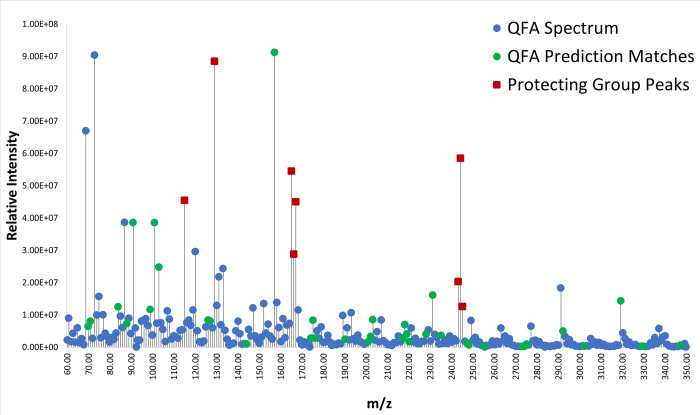
QFA DEP-EI-mass spectrum compared with predicted peaks. In blue are all plotted peaks observed in the spectrum for the QFA peptide. In green are peaks that match with predictions generated using our model. Red squares mark peaks associated with protecting groups used in the synthesis of the peptides. A peak was considered a match if it was within the max instrumental error (+/- 0.25 *m/z*) of the mass spectrometer. The maximum peak intensity is 9.12 X 10^7^ counts.

**Fig 6:**
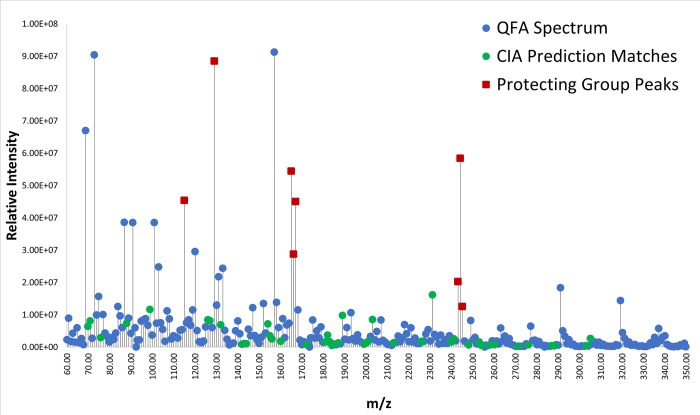
QFA DEP-EI-mass spectrum compared with predicted peaks for the sequence CIA. As a counter example for evaluating the program’s predictions, peak matching was done between an incorrect sequence, CIA, and the QFA mass spectrum. In blue are all plotted peaks observed in the spectrum for the QFA peptide. In green are peaks that match with CIA fragmentation predictions generated using our model. Red squares mark peaks associated with protecting groups used in the synthesis of the peptides. A peak was considered a match if it was within the max instrumental error (+/- 0.25 *m/z*) of the mass spectrometer. The maximum peak intensity is 9.12 X 10^7^ counts.

When the spectra were compared against the predictions made by the program model, it was found that the program identified several locations of major clusters within the spectrum, particularly when peaks associated with protecting groups were removed. For example, the QFA spectrum (Fig 5) shows matches at nearly every major non-protecting group peak except for peaks at approximately 69, 73, 87, 120, 131, 133, 147, 152, 168, and 291 *m/z*. The peaks at 69, 73, and 87 occur in nearly all spectra, and so are unlikely to be helpful in distinguishing between different peptides. These results suggest that the model and prediction algorithm already do a fair job at “fingerprinting” a peptide, although there is room for further improvement. As a means of confirming that the prediction model can actually be used to tell distinct peptides apart, a comparison analysis was done using the prediction for the peptide CIA but comparing with the spectrum for QFA. Because these peptides have very distinct compositions and amino acids with different fragmentation patterns, it was expected that the comparison would have fewer matches among the more prominent peaks if the prediction algorithm truly is doing a reasonable job of fingerprinting different peptides. Fig 6 shows the results of this comparison. There are a fair number of matches within an error margin of 0.25 *m/z* (the maximum instrument error as determined by comparing the instrument’s calibration values against the standard gas actual values over mass ranges being measured). However, none of those matches are associated with the most abundant peaks within the spectrum. This suggests that the predictions may have some utility in distinguishing whether a spectrum is likely to represent a given sequence, for a subset of peptides.

Examining the different tripeptides, it also becomes apparent that the model does better with some sequences than others, suggesting that additional mechanisms remain to be identified. For example, the CIA spectrum (S1 Fig) had fewer major non-protecting group associated peaks, and while the prediction algorithm matched several of those peaks, several peaks remain unmatched which may correspond to fragmentation mechanisms not yet identified and incorporated into the model.

**Fig 7:**
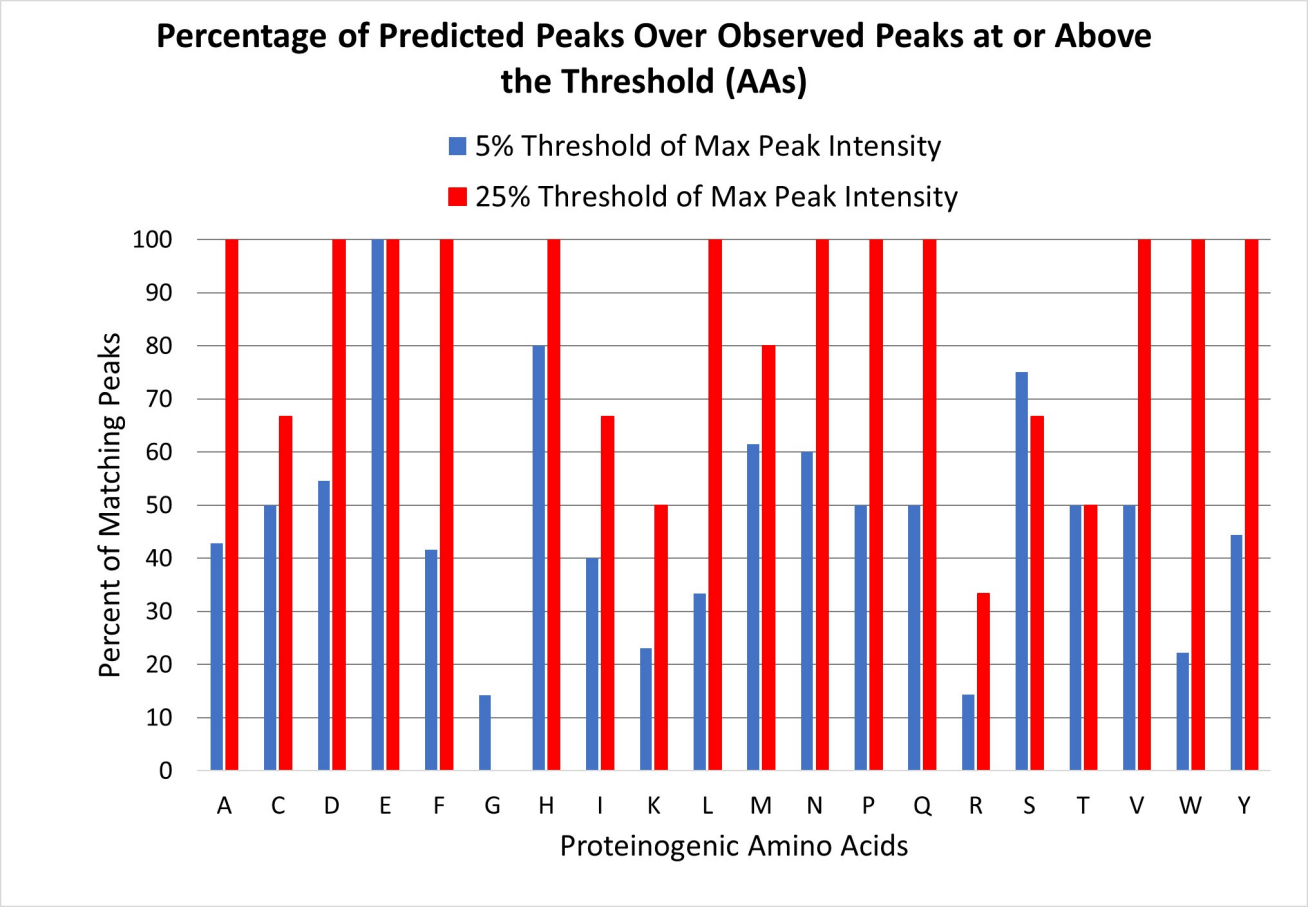
Percent matches of proteinogenic amino acids. Percentages of prediction-matching peaks relative to all peaks were calculated using peaks whose intensity met or exceeded the threshold intensity relative to the most intense peak in the spectrum.

**Fig 8:**
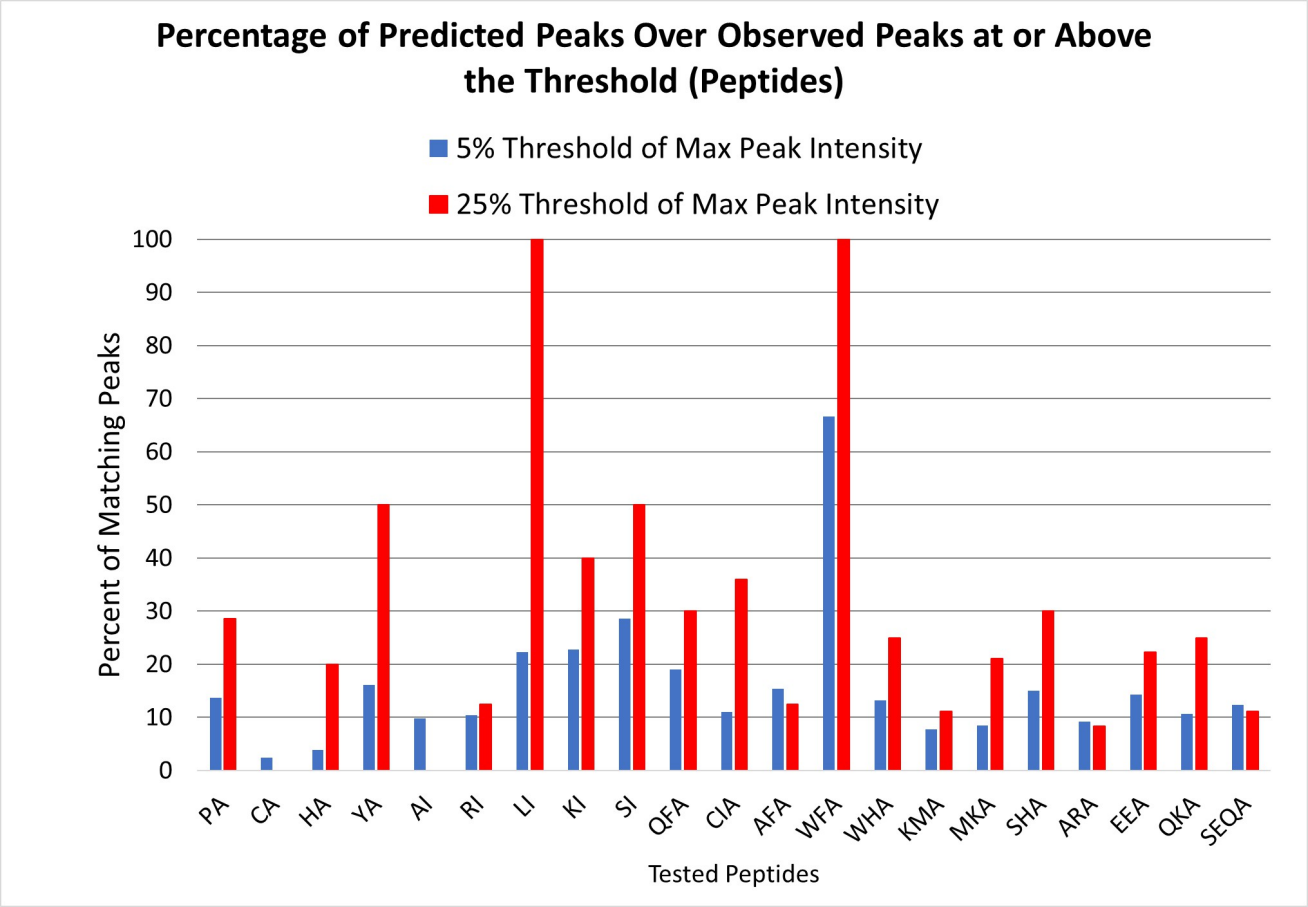
Percent matches of crude synthetic peptides. Percentages of prediction-matching peaks relative to all peaks were calculated using peaks whose intensity met or exceeded the threshold intensity relative to the most intense peak in the spectrum.

As mentioned above, peak matches had an enforced upper error of 0.25 *m/z*. However, the average uncertainty between the predicted mass and the matched mass in the spectrum across all peak matches was lower than this at 0.14 +/- 0.074 *m/z*. Our prediction scoring analysis (Figs 7 and 8) confirmed that the model had good coverage of the major peaks for most amino acids, with the exception of Gly (which has no amino-acid specific identified degradation mechanisms in the model), and Arg. At the low threshold of 5% where a larger number of peaks were included in the analysis, the amino acids averaged to about 48% of the spectrum matched across all amino acids, with match percentages ranging from 14% to 100% depending on the amino acid. At the more stringent intensity threshold of 25%, the scores improved significantly with an average match across all amino acids of about 80% with scores ranging from 0% (Gly) to 100%. These data suggest that the model does well at detecting the most intense amino acid peaks, but can still benefit from improvement for the lower intensity peaks. They also emphasize that the model has significant weaknesses in regards to a small subset of amino acids (namely Gly and Arg). However, glycine’s lack of side chain structure limits the diagnostic fragmentation mechanisms available to it. That leaves arginine as the weakest portion of the model in its current form.

**Fig 9:**
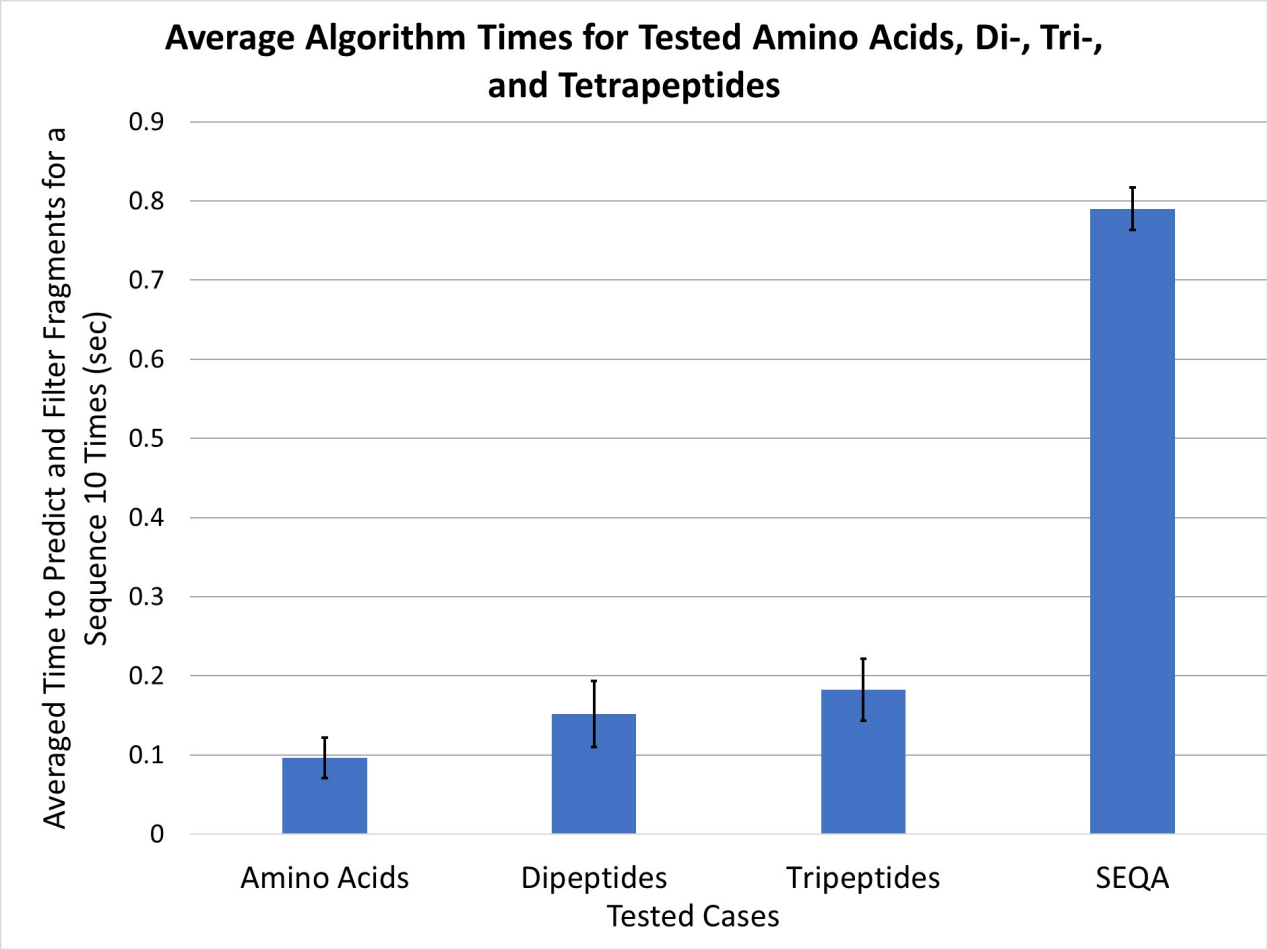
Average algorithm run times. The average run times for the 20 proteinogenic amino acids as well as the di-, tri-, and tetrapeptides (SEQA) investigated in this work were evaluated by timing how long it took the algorithm to generate and filter its predictions for each sequence 10 times.

The algorithm was also assessed to determine an estimated run time for predictions covering the amino acids and sequences discussed (Fig 9). As expected, as the number of possible fragments increased with peptide length, the run time increased as well. Notably, our tetrapeptide sequence SEQA, which includes high-fragment amino acids Glu and Gln, showed a significant jump in run time (from just under 0.2 sec to nearly 0.8 sec) compared to the single amino acids and the di- and tri- peptides tested. However, all tested sequences averaged to less than one second of processing time to conduct the analysis over 10 iterations.

### Next steps in model improvement

Under the conditions used in this study, some fragments are far more prevalent than others for a given amino acid or peptide. The peaks associated with these fragments therefore have greater potential for detection and use as diagnostic peaks. A possible improvement for the model would involve identifying those peaks that consistently exhibit higher associated abundances and using them to designate certain peaks as being more likely to be visible in a spectrum. However, at the individual amino acid level, very few peaks were seen to be unique to a given amino acid. Even in the peptides tested, often the most intense peaks have lower *m/z* values that can overlap with fragments from other sources. This expected result reinforces the importance of using multiple peak matches within the “fingerprint” to make the decision on whether a peptide match is present or not. Finding and adding more mechanisms to the model, particularly for amino acids that currently have lower prediction fidelity such as arginine, will allow the current model’s predictions to become more robust. Because of the enormous sequence space represented by the different amino acid arrangements in peptides as they get larger, the development of a high-throughput means of both synthesizing peptides and collecting their EI-MS data would be an invaluable tool to facilitate further improvements to the current model by allowing a larger amount of the sequence space to be sampled quickly.

## Conclusions

We have confirmed several mechanisms of fragmentation observed in EI-MS experiments and have organized these mechanisms into a predictive model using Python. In some cases, mechanisms can be applied in a combinatorial manner to make predictions for EI-MS fragmentation both within individual amino acids and within peptides (for example, in the case of carboxylic acid and/or amide degradation). This initial predictive model has had reasonable success in confirming the observed peaks seen in the spectra, particularly for spectra incorporating amino acids whose available fragmentation mechanisms appear to have been more fully identified or covered by the model. However, it is also clear that additional mechanisms remain to be identified and incorporated into the model to further enhance its predictions. Further, considering reproducible relative peak intensities in the future may help further refine predictions. To our knowledge, this work reflects the first EI-MS prediction model for peptides to be developed, and represents a major step towards being able to identify probable amino acid content of peptide samples, and provides a tool that can aid in determining whether a desired peptide is present in a sample analyzed using EI-MS by providing a list of possible *m/z* values for comparison with experimental spectra.

## Materials and methods

### General

Unless otherwise specified, all 20 proteinogenic unprotected amino acids were purchased from Sigma Aldrich while resins and Fmoc- and associated side-chain-protected amino acids used for peptide synthesis were purchased from aapptec. Chemicals used for protecting group peak analysis such as trityl-chloride, 2,2,4,6,7-pentamethldihydrobenzofuran-5-sulfonyl (pbf) chloride, and ditertbutylcarbamate were purchased through Thermo Fisher Scientific. All reagents were used without additional purification. All mass spectrometry sample solutions were prepared in 18 MΩ water and HPLC-grade acetonitrile (Thermo Fisher Scientific). All peptide synthesis reactions were conducted manually in 6 mL polypropylene reaction vessels containing porous frits with washing of the resin with *N*,*N*-dimethylformamide (DMF) and/or dichloromethane (DCM) between all reaction steps. Unless otherwise noted, peptide synthesis was conducted using Wang resin preloaded with either Ala (loading capacity of 0.503 mmol/g) or Ile (loading capacity of 0.33 mmol/g). Peptide syntheses were conducted on 0.05 mmol scales in approximately 2 mL of reaction solution. The Python script that implements the prediction model was developed and tested on Python versions 3.6.4 through 3.9.7. The source code and definition files containing the fragmentation mechanism data are publicly available on Github (See S2 File for a link).

### Fmoc removal

Fmoc removal was accomplished through treatment of the resin with 2 mL of 2% piperidine and 2% 1,8-Diazabicyclo[5.4.0]undec-7-ene (DBU) in DMF and shaking for 6 min. This process was repeated once more (for a total of 12 min deprotection time). The resin was then washed three times with DMF, shaking for 1 min each wash.

### HBTU and DIPEA-facilitated coupling

Amino acid coupling steps were accomplished by using 2-(1H-benzotriazol-1-yl)-1,1,3,3-tetramethyluronium hexafluorophosphate (HBTU) as the coupling reagent and *N*,*N*-diisopropylethylamine (DIPEA) as the base. 2 equiv of the desired amino acid were combined with 1.95 equiv of HBTU before being dissolved in approximately 2 mL of DMF. 4 equiv of DIPEA were then added, and the solution was shaken for 1 minute before addition to the resin and reaction for 2 to 16 h. Following coupling, the resin was washed three times with DMF, shaking for 1 min each wash.

### Chloranil test

The chloranil test for primary and secondary amines was conducted as previously described [12]. Briefly, a small amount of dried resin was placed in a microcentrifuge tube. To the dried resin was added 2 drops of 2% (v/v) acetaldehyde in DMF and two drops of 2% (w/v) p-chloranil in DMF. The mixture was allowed to stand for 4–6 min at room temperature. Dark blue to green-stained resin beads indicated the presence of unreacted primary and/or secondary amines.

### Final cleavage

The resin was transferred from the original reaction vessel to a 15 mL polypropylene centrifuge tube. 3 mL of 2.5% water and 2.5% Triisopropylsilane (TIPS) in Trifluoroacetic acid (TFA) was added and the tube was shaken for 3 h. The resin was filtered through a cotton plug in a polypropylene syringe and washed with a small amount of TFA. The filtrate was collected and the solvent evaporated prior to further use.

### Collection of DEP-EI-MS spectra

A DSQ II mass spectrometer (Thermo Fisher Scientific) with a direct exposure probe was used for all DEP-EI-MS experiments. Prior to data collection, the DEP sample loop was inserted into the instrument and cleaned 5 times initially and 3–4 times in between separate sample reads. Amino acid, peptide, and protecting group control solutions were prepared at concentrations of approximately 2–5 mg/mL in mixtures of water and acetonitrile that ranged from 0% ACN to 75% ACN depending on the solubility of each amino acid or peptide. While outside of the mass spectrometer, 1 μL of the solution was pipetted onto the tip of the sample loop at the end of the probe and the probe was allowed to stand until all the solvent had evaporated. The probe was then inserted into the mass spectrometer and a spectrum was collected in positive ion mode using the following conditions: After a 30 sec hold at 0 mA, the current was ramped at 20 mA/s up to 1000 mA. Spectral data was collected with a scan rate of 3500 amu/s over a mass range of 60 to 350 *m/z*. One exception to these conditions was made for Gly, which was scanned over a narrower mass range from 10 to 250 *m/z*. The ion volume where the sample loop was inserted was maintained at 200°C for at least 2 hours prior to calibration and throughout the data collection. All spectra were recorded in duplicate with each replicate collected from a freshly prepared sample.

### Building the initial model in Python

Our initial model defines all structures by a formula list: [#C,#H,#N,#O,#S,charge]. Since only positive ion mode spectra were considered in this study, all fragmentation definitions are associated with that mode of detection. The backbone cleavage mechanisms identified by Seifert et al. [3] were considered to be intrinsic to all peptides and were hard-coded, along with *C*-terminus degradation, into the Python program used for the prediction model. The side-chain fragmentation definitions for amino acids and their associated fragmentation events were instead placed in a data text file so that the program can be updated with new fragmentation mechanism data as they become available without needing to change the code itself. This also means it should be possible to include nonconventional amino acids or possibly protected amino acids in the future by updating this definition file. The data structures used in this initial model are as summarized below:

An AminoAcid class which stores the amino acid’s name (e.g. “A1” to indicate the first alanine in a sequence), its formula list, a classification of its side chain (e.g. “alcohol”, “acidic”) for use in data analysis later, a list of fragmentation formula lists associated with that amino acid, and a string fragmentation label used to trace multiple fragmentation events during the prediction process. AminoAcid objects contain add and subtract formula methods to allow their own formula to be updated from a supplied formula list, and a higher order fragment method that would allow a given amino acid to generate its own predicted fragmentations as defined in the definition file.

A PepFrag class which stores a list of AminoAcid objects (representing a peptide sequence) and a string that stores the cumulative fragmentation record during the prediction process. PepFrag objects have methods that allow them to form peptide bonds (done during initialization to update the formula appropriately after being given the sequence of amino acids), add/subtract formula methods, and a fragment method that initializes the fragment prediction process. Additional methods that are called as part of the fragment method include ones that handle Type-A, B, or C backbone cleavage, and a side chain fragment generator as well as methods that calculate the monoisotopic mass of the PepFrag based on its formula.

The generation of fragment predictions by the program follows a general protocol. First, the original peptide sequence is systematically cleaved with all possible backbone cleavage events, starting with Type A, then Type B, and finally Type C. The resulting fragments are pooled and then each is systematically subjected to side chain fragmentation for all amino acids within its sequence. This process is then repeated for all sub-fragments and all results are pooled. In this way, an entire peptide sequence can be iterated through, generating a thorough list of possible fragments that may be observable in the spectrum. The “mix and match” process assumes that there will be at most only one side-chain fragmentation event per each individual amino acid in a given fragmentation event. This assumption was made since significant portions of the side chain are lost in the fragmentation events, meaning that conservation of side chain mass would preclude having two fragmentation events occur simultaneously. Further, since side chain fragmentation definitions were derived from associated peaks in spectra, their combination within the same amino acid should be unnecessary.

In some cases, the thoroughness of the programmed systematic approach creates duplicate fragment predictions. Final results are therefore filtered to remove these duplicates before outputting them. In some rare cases, the combination of considered fragmentation events for single amino acids result in negative mass predictions (indicating overlap in the predicted loss of atoms). Those predictions are also filtered out prior to output of the results. The program also includes functions that allow mass spectrum peak data to be loaded from text files and compared with the generated predictions and output an “annotated” text file with peaks labeled with matching fragments, background peaks, and/or the peak with the greatest intensity.

Because our experiments have shown that some fragmentation events cannot be combinatorial, several key words are reserved for use by the program and/or definition file to avoid the combination of mutually exclusive fragmentation events. The word “type” is reserved to indicate backbone cleavage and should not be included in fragment event definition labels. Likewise, “charged” is used to indicate that the fragmentation event is associated with a non-relative mass fragmentation event (e.g. the defined structure formula will be directly detected as an ion), while “side” is used to associate a fragmentation with a side chain (primarily to distinguish it from a *C*-terminus degradation when conducting the mixing and matching of fragmentation types).

### Scoring predictions against their associated experimental spectra

As a means of scoring the fit between a set of predictions and mass spectral data, we used threshold values relative to the most intense peak in the spectrum as cut-offs for choosing the peaks to compare. This analysis was conducted on the spectra for all 20 proteinogenic amino acids as well as for all of the synthetic peptides. Since the peptide spectra were of crude peptides, a list of potential contaminants (such as the protecting groups used) was used to remove peaks matching an expected contaminant from the analysis. We used a low threshold value of 5% relative to the highest intensity peak to evaluate how the prediction model performed when most peaks would be counted in the assessment. To evaluate how the model performed at detecting high intensity peaks, a threshold value of 25% was used. A fitness score was generated by calculating the percentage of peaks with intensities equal to or greater than the given threshold that matched a prediction from the model relative to the total number of peaks in the spectrum whose intensities met the threshold. A peak was counted as a match if it was within the maximum instrumental error within the range scanned (+/- 0.25 *m/z*).

### Timing the prediction algorithm

The built-in time.time() function provided with Python was used to estimate the time it took the algorithm to generate predictions for each of the 20 proteinogenic amino acids as well as for each of the peptides used in this study. In each case, the time it took the algorithm to reach the point that results would be ready to be displayed (after generation and filtering of duplicates/negative predictions) 10 times. The timing evaluation was conducted using Python version 3.9.7 on a computer running Windows 10 and using an 11^th^ Gen Intel® Core™ i7-11700 @ 2.50 GHz processor.

## Supporting information

S1 Table Crude synthetic dipeptides used for prediction comparison. (PDF)

S1 Fig CIA DEP-EI-mass spectrum compared with predicted peaks. In blue are all plotted peaks observed in the spectrum for the CIA peptide. In green are peaks that match with predictions generated using our model. Red squares mark peaks associated with protecting groups used in the synthesis of the peptides. A peak was considered a match if it was within the max instrumental error (+/- 0.25 *m/z*) of the mass spectrometer.
(TIF)

S2 Fig AFA DEP-EI-mass spectrum compared with predicted peaks. In blue are all plotted peaks observed in the spectrum for the AFA peptide. In green are peaks that match with predictions generated using our model. Red squares mark peaks associated with protecting groups used in the synthesis of the peptides. A peak was considered a match if it was within the max instrumental error (+/- 0.25 *m/z*) of the mass spectrometer.
(TIF)

S3 Fig WFA DEP-EI-mass spectrum compared with predicted peaks. In blue are all plotted peaks observed in the spectrum for the WFA peptide. In green are peaks that match with predictions generated using our model. Red squares mark peaks associated with protecting groups used in the synthesis of the peptides. A peak was considered a match if it was within the max instrumental error (+/- 0.25 *m/z*) of the mass spectrometer.
(TIF)

S4 Fig WHA DEP-EI-mass spectrum compared with predicted peaks. In blue are all plotted peaks observed in the spectrum for the WHA peptide. In green are peaks that match with predictions generated using our model. Red squares mark peaks associated with protecting groups used in the synthesis of the peptides. A peak was considered a match if it was within the max instrumental error (+/- 0.25 *m/z*) of the mass spectrometer.
(TIF)

S5 Fig KMA DEP-EI-mass spectrum compared with predicted peaks. In blue are all plotted peaks observed in the spectrum for the KMA peptide. In green are peaks that match with predictions generated using our model. Red squares mark peaks associated with protecting groups used in the synthesis of the peptides. A peak was considered a match if it was within the max instrumental error (+/- 0.25 *m/z*) of the mass spectrometer.
(TIF)

S6 Fig MKA DEP-EI-mass spectrum compared with predicted peaks. In blue are all plotted peaks observed in the spectrum for the MKA peptide. In green are peaks that match with predictions generated using our model. Red squares mark peaks associated with protecting groups used in the synthesis of the peptides. A peak was considered a match if it was within the max instrumental error (+/- 0.25 *m/z*) of the mass spectrometer.
(TIF)

S7 Fig SHA DEP-EI-mass spectrum compared with predicted peaks. In blue are all plotted peaks observed in the spectrum for the SHA peptide. In green are peaks that match with predictions generated using our model. Red squares mark peaks associated with protecting groups used in the synthesis of the peptides. A peak was considered a match if it was within the max instrumental error (+/- 0.25 *m/z*) of the mass spectrometer.
(TIF)

S8 Fig ARA DEP-EI-mass spectrum compared with predicted peaks. In blue are all plotted peaks observed in the spectrum for the ARA peptide. In green are peaks that match with predictions generated using our model. Red squares mark peaks associated with protecting groups used in the synthesis of the peptides. A peak was considered a match if it was within the max instrumental error (+/- 0.25 *m/z*) of the mass spectrometer.
(TIF)

S9 Fig EEA DEP-EI-mass spectrum compared with predicted peaks. In blue are all plotted peaks observed in the spectrum for the EEA peptide. In green are peaks that match with predictions generated using our model. Red squares mark peaks associated with protecting groups used in the synthesis of the peptides. A peak was considered a match if it was within the max instrumental error (+/- 0.25 *m/z*) of the mass spectrometer.
(TIF)

S10 Fig QKA DEP-EI-mass spectrum compared with predicted peaks. In blue are all plotted peaks observed in the spectrum for the QKA peptide. In green are peaks that match with predictions generated using our model. Red squares mark peaks associated with protecting groups used in the synthesis of the peptides. A peak was considered a match if it was within the max instrumental error (+/- 0.25 *m/z*) of the mass spectrometer.
(TIF)

S11 Fig SEQA DEP-EI-mass spectrum compared with predicted peaks. In blue are all plotted peaks observed in the spectrum for the SEQA peptide. In green are peaks that match with predictions generated using our model. Red squares mark peaks associated with protecting groups used in the synthesis of the peptides. A peak was considered a match if it was within the max instrumental error (+/- 0.25 *m/z*) of the mass spectrometer.
(TIF)

S12 Fig PA DEP-EI-mass spectrum compared with predicted peaks. In blue are all plotted peaks observed in the spectrum for the PA peptide. In green are peaks that match with predictions generated using our model. Red squares mark peaks associated with protecting groups used in the synthesis of the peptides. A peak was considered a match if it was within the max instrumental error (+/- 0.25 *m/z*) of the mass spectrometer.
(TIF)

S13 Fig CA DEP-EI-mass spectrum compared with predicted peaks. In blue are all plotted peaks observed in the spectrum for the CA peptide. In green are peaks that match with predictions generated using our model. Red squares mark peaks associated with protecting groups used in the synthesis of the peptides. A peak was considered a match if it was within the max instrumental error (+/- 0.25 *m/z*) of the mass spectrometer.
(TIF)

S14 Fig HA DEP-EI-mass spectrum compared with predicted peaks. In blue are all plotted peaks observed in the spectrum for the HA peptide. In green are peaks that match with predictions generated using our model. Red squares mark peaks associated with protecting groups used in the synthesis of the peptides. A peak was considered a match if it was within the max instrumental error (+/- 0.25 *m/z*) of the mass spectrometer.
(TIF)

S15 Fig YA DEP-EI-mass spectrum compared with predicted peaks. In blue are all plotted peaks observed in the spectrum for the YA peptide. In green are peaks that match with predictions generated using our model. Red squares mark peaks associated with protecting groups used in the synthesis of the peptides. A peak was considered a match if it was within the max instrumental error (+/- 0.25 *m/z*) of the mass spectrometer.
(TIF)

S16 Fig AI DEP-EI-mass spectrum compared with predicted peaks. In blue are all plotted peaks observed in the spectrum for the AI peptide. In green are peaks that match with predictions generated using our model. Red squares mark peaks associated with protecting groups used in the synthesis of the peptides. A peak was considered a match if it was within the max instrumental error (+/- 0.25 *m/z*) of the mass spectrometer.
(TIF)

S17 Fig RI DEP-EI-mass spectrum compared with predicted peaks. In blue are all plotted peaks observed in the spectrum for the RI peptide. In green are peaks that match with predictions generated using our model. Red squares mark peaks associated with protecting groups used in the synthesis of the peptides. A peak was considered a match if it was within the max instrumental error (+/- 0.25 *m/z*) of the mass spectrometer.
(TIF)

S18 Fig LI DEP-EI-mass spectrum compared with predicted peaks. In blue are all plotted peaks observed in the spectrum for the LI peptide. In green are peaks that match with predictions generated using our model. Red squares mark peaks associated with protecting groups used in the synthesis of the peptides. A peak was considered a match if it was within the max instrumental error (+/- 0.25 *m/z*) of the mass spectrometer.
(TIF)

S19 Fig KI DEP-EI-mass spectrum compared with predicted peaks. In blue are all plotted peaks observed in the spectrum for the KI peptide. In green are peaks that match with predictions generated using our model. Red squares mark peaks associated with protecting groups used in the synthesis of the peptides. A peak was considered a match if it was within the max instrumental error (+/- 0.25 *m/z*) of the mass spectrometer.
(TIF)

S20 Fig SI DEP-EI-mass spectrum compared with predicted peaks. In blue are all plotted peaks observed in the spectrum for the SI peptide. In green are peaks that match with predictions generated using our model. Red squares mark peaks associated with protecting groups used in the synthesis of the peptides. A peak was considered a match if it was within the max instrumental error (+/- 0.25 *m/z*) of the mass spectrometer.
(TIF)

S21 Fig Annotated DEP-EI-MS fragmentation spectrum for Phe. Several fragmentation mechanisms are present in the Phe spectrum. This includes two non-relative fragmentation mechanisms where the observed peak corresponds to the released side chain (with or without the apparent formation of an alkene). Beyond the common loss of side chain mechanism shared by all of the amino acids, two relative fragmentation mechanisms include Type A-like cleavage or the loss of the C-terminal carboxylic acid group and a Type C cleavage without formation of an alkene (nd). The maximum peak intensity for the shown spectrum is 4.64 X 10^8^ counts. Proposed structures are shown along with the resulting fragment formula and monoisotopic *m/z*. ^a^degradation type also observed in *[11]*, ^b^degradation type also observed in *[3]*.
(TIF)

S22 Fig Annotated DEP-EI-MS fragmentation spectrum for Tyr. Several fragmentation mechanisms are present in the Tyr spectrum. Similar to Phe, there are two non-relative fragmentation mechanisms where the observed peak corresponds to the released side chain (with or without the apparent formation of an alkene). The common loss of side chain mechanism is present as well as a peak for the molecular (non-fragmented) ion. Type A-like cleavage or the loss of the C-terminal carboxylic acid group composes the last of the identified fragmentation mechanisms. The maximum peak intensity for the shown spectrum is 3.62 X 10^8^ counts. Proposed structures are shown along with the resulting fragment formula and monoisotopic *m/z*. ^a^degradation type also observed in *[11]*, ^b^degradation type also observed in *[3]*.
(TIF)

S23 Fig Annotated DEP-EI-MS fragmentation spectrum for Trp. Several fragmentation mechanisms are present in the Trp spectrum. Only one non-relative fragmentation mechanism was observed, appearing to release the charged side chain similar to the other conjugated amino acids. The peak corresponding to the loss of the side chain was present, but at a much lower intensity than for most of the other amino acids. The molecular ion was observed along with a Type A-like cleavage or the loss of the C-terminal carboxylic acid group. The remaining mechanisms involved Type-C cleavage with and without alkene formation. The maximum peak intensity for the shown spectrum is 5.54 X 10^8^ counts. Proposed structures are shown along with the resulting fragment formula and monoisotopic *m/z*. ^a^degradation type also observed in [11], ^b^degradation type also observed in [3].
(TIF)

S24 Fig Annotated DEP-EI-MS fragmentation spectrum for His. Several fragmentation mechanisms are present in the His spectrum. Similar to Phe and Tyr, there are two non-relative fragmentation mechanisms where the observed peak corresponds to the released side chain (with or without the apparent formation of an alkene). In addition, a third non-relative mechanism involves release of a protonated side chain with radical formation. Type A-like cleavage or the loss of the C-terminal carboxylic acid group composes the last of the identified fragmentation mechanisms. The maximum peak intensity for the shown spectrum is 7.83 X 10^7^ counts. Proposed structures are shown along with the resulting fragment formula and monoisotopic *m/z*. ^a^degradation type also observed in *[11]*, ^b^degradation type also observed in *[3]*.
(TIF)

S25 Fig Annotated DEP-EI-MS fragmentation spectrum for Met. Several fragmentation mechanisms are present in the Met spectrum. Two non-relative fragmentation mechanisms were observed, releasing different charged sub-fragments of the side chain. The common full loss of side chain mechanism was observed as well as a partial loss of side chain. Type A-like cleavage or the loss of the C-terminal carboxylic acid group was observed along with the C-terminal loss of water. A peak corresponding to the molecular ion was also observed at a relatively high intensity. The maximum peak intensity for the shown spectrum is 1.19 X 10^8^ counts. Proposed structures shown along with the resulting fragment formula and monoisotopic *m/z*. ^a^degradation type also observed in [11], ^b^degradation type also observed in [3].
(TIF)

S26 Fig Annotated DEP-EI-MS fragmentation spectrum for Leu. Several fragmentation mechanisms are present in the Leu spectrum. The common side chain loss mechanism is present along with a Type A-like cleavage resulting in the loss of the C-terminal carboxylic acid group. Peaks associated with partial loss of the side chain were observed in combination with loss of either water or a hydroxyl loss from the C-terminus. The maximum peak intensity for the shown spectrum is 8.59 X 10^7^ counts. Proposed structures are shown along with the resulting fragment formula and monoisotopic *m/z*. ^a^degradation type also observed in [11], ^b^degradation type also observed in [3].
(TIF)

S27 Fig Annotated DEP-EI-MS fragmentation spectrum for Ser. Several fragmentation mechanisms are present in the Ser spectrum. No non-relative peak fragmentation mechanisms were seen. The common full loss of side chain mechanism was observed. Peaks corresponding to β-elimination and C-terminal loss of water as well as β-elimination and loss of the C-terminal hydroxyl group are observed. No peak corresponding to β-elimination alone was observed. Type A-like cleavage or the loss of the C-terminal carboxyl group was observed. C-terminal loss of water was seen as well as the protonated molecular ion. The maximum peak intensity for the SerOH spectrum is 1.12 X 10^8^ counts. Proposed structures are shown along with the resulting fragment formula and monoisotopic *m/z*. ^a^degradation type also observed in [11], ^b^degradation type also observed in [3].
(TIF)

S28 Fig Annotated DEP-EI-MS fragmentation spectrum for Thr. Several fragmentation mechanisms are present in the Thr spectrum. No non-relative peak fragmentation mechanisms were identified. The peak at a *m/z* of 74.0 could correspond either to the common side chain loss mechanism, or due to Type A-like cleavage or the loss of the C-terminal carboxylic acid group. Two β-elimination peaks combined with the loss of a C-termina water or hydroxyl group are observed. A peak corresponding to the loss of water from the C-terminus is also present. The maximum peak intensity for the shown spectrum is 8.79 X 10^7^ counts. Proposed structures are shown along with the resulting fragment formula and monoisotopic *m/z*. ^a^degradation type also observed in [11], ^b^degradation type also observed in [3].
(TIF)

S29 Fig Annotated DEP-EI-MS fragmentation spectrum for Cys. Several fragmentation mechanisms are present in the Cys spectrum. No non-relative fragmentation mechanisms were seen. The common full loss of the side chain mechanism was observed. Peaks corresponding to β-elimination alone, β-elimination and loss of the C-terminal hydroxyl group, and β-elimination and C-terminal loss of water were observed. Type A-like cleavage or the loss of the C-terminal carboxyl group was seen, as well as a peak corresponding to the molecular ion. The maximum peak intensity for Cys spectrum is 2.83 X 10^8^ counts. Proposed structures are shown along with the resulting fragment formula and monoisotopic *m/z*. ^a^degradation type also observed in [11], ^b^degradation type also observed in [3].
(TIF)

S30 Fig Annotated DEP-EI-MS fragmentation spectrum for Glu. Several fragmentation mechanisms are present in the Glu spectrum. No non-relative peak fragmentation mechanisms were seen. The common full loss of side chain mechanism was observed. Peaks corresponding to Type A-like cleavage or the loss of the C-terminal carboxylic acid group and side chain water loss, Type A-like cleavage and loss of the C-terminal hydroxyl group, and Type A-like cleavage or C-terminal carboxylic acid loss were seen. A peak corresponding to either C-terminal or side chain water loss was observed. A peak corresponding to either C-terminal or side chain hydroxyl group loss was observed. The maximum peak intensity for the shown spectrum is 3.11 X 10^8^ counts. Proposed structures are shown along with the resulting fragment formula and monoisotopic *m/z*. ^a^degradation type also observed in [11], ^b^degradation type also observed in [3].
(TIF)

S31 Fig Annotated DEP-EI-MS fragmentation spectrum for Asp. Several fragmentation mechanisms are present in the Asp spectrum. No non-relative peak fragmentation mechanisms were seen. The common full loss of side chain mechanism was observed. Peaks corresponding to Type A-like cleavage and side chain water loss, Type A-like cleavage and loss of the side chain hydroxyl group, and Type A-like cleavage or C-terminal carboxylic acid loss were seen. Peaks corresponding to side chain water loss and C-terminal water loss, and side chain hydroxyl loss and C-terminal hydroxyl loss were observed. A peak corresponding to either side chain water loss and C-terminal hydroxyl loss, or side chain hydroxyl loss and C-terminal water loss was seen. A peak corresponding to either C-terminal or side chain water loss was observed. A peak corresponding to either C-terminal or side chain hydroxyl group loss was observed. The maximum peak intensity for the shown spectrum is 8.80 X 10^7^ counts. Proposed structures are shown along with the resulting fragment formula and monoisotopic *m/z*. ^a^degradation type also observed in [11], ^b^degradation type also observed in [3].
(TIF)

S32 Fig Annotated DEP-EI-MS fragmentation spectrum for Lys. Several fragmentation mechanisms are present in the Lys spectrum. No non-relative peak fragmentation mechanisms were seen. The common full loss of side chain mechanism was observed. Peaks corresponding to Type A-like cleavage and side chain cyclization, Type A-like cleavage and side chain alkene formation, and Type A-like cleavage or C-terminal carboxylic acid loss were seen. Peaks corresponding to side chain cyclization and C-terminal hydroxyl loss, and side chain cyclization and C-terminal water loss were observed. A peak indicating C-terminal hydroxyl loss was seen, as well as a peak indicating C-terminal water loss. The maximum peak intensity for the shown spectrum is 1.44 X 10^8^ counts. Proposed structures are shown along with the resulting fragment formula and monoisotopic *m/z*. ^a^degradation type also observed in [11], ^b^degradation type also observed in [3].
(TIF)

S33 Fig Annotated DEP-EI-MS fragmentation spectrum for Arg. Several fragmentation mechanisms are present in the Arg spectrum. No non-relative peak fragmentation mechanisms were seen. Loss of side chain guanidino group with alkene formation and C-terminal carboxylic acid loss was seen. Peaks corresponding to partial loss of guanidino group with proton gain and C-terminal water loss, as well as partial loss of guanidino group with proton gain and C-terminal carboxylic acid loss or Type A-like cleavage, were observed. A peak corresponding to Type A-like cleavage of C-terminal carboxylic acid loss was seen. The common full loss of side chain mechanism was observed. Loss of the guanidino group with alkene formation and C-terminal hydroxyl loss was noted. Partial loss of the guanidino group with proton gain and C-terminal hydroxyl loss was also observed. The maximum peak intensity for the shown spectrum is 2.73 X 10^7^ counts. Apparent structures are shown along with the resulting fragment formula and monoisotopic *m/z*. ^a^degradation type also observed in [11], ^b^degradation type also observed in [3].
(TIF)

S34 Fig Annotated DEP-EI-MS fragmentation spectrum for Pro. Several fragmentation mechanisms are present in the Pro spectrum. No non-relative peak fragmentation mechanisms were seen. Peaks corresponding to Type A-like cleavage or C-terminal carboxylic acid loss, and Type A-like cleavage or C-terminal carboxylic acid loss and side chain alkene formation were observed. Peaks corresponding to side chain alkene formation and C-terminal water loss, and side chain alkene formation and C-terminal hydroxyl loss were observed. The protonated molecular ion was also observed. The maximum peak intensity for the shown spectrum is 3.60 X 10^8^ counts. Proposed structures are shown along with the resulting fragment formula and monoisotopic *m/z*. ^a^degradation type also observed in [11], ^b^degradation type also observed in [3].
(TIF)

S35 Fig Annotated DEP-EI-MS fragmentation spectrum for Ile. Several fragmentation mechanisms are present in the Ile spectrum. No non-relative peak fragmentation mechanisms were seen. Peaks corresponding to side chain loss and partial side chain loss were seen. Peaks corresponding to Type A-like cleavage or C-terminal carboxylic acid loss and side chain alkene formation, and Type A-like cleavage or C-terminal carboxylic acid loss were also observed. The maximum peak intensity for the shown spectrum is 2.99 X 10^8^ counts. Proposed structures are shown along with the resulting fragment formula and monoisotopic *m/z*. ^a^degradation type also observed in [11], ^b^degradation type also observed in [3].
(TIF)

S36 Fig Annotated DEP-EI-MS fragmentation spectrum for Val. Several fragmentation mechanisms are present in the Val spectrum. No non-relative peak fragmentation mechanisms were seen. Type A-like cleavage or C-terminal carboxylic acid loss was observed. Peaks corresponding to side chain loss, and partial side chain loss and C-terminal water loss were seen. C-terminal water loss was also observed. The maximum peak intensity for the shown spectrum is 7.92 X 10^7^ counts. Proposed structures are shown along with the resulting fragment formula and monoisotopic *m/z*. ^a^degradation type also observed in [11], ^b^degradation type also observed in [3].
(TIF)

S1 File Collection of representative DEP-EI mass spectra for Ala, Gly, and synthesis protecting groups. Representative spectra for the proteinogenic amino acids alanine and glycine are included along with spectra corresponding to the Trt, Fmoc, Pbf, and Boc/t-Bu protecting groups.
(PDF)

S2 File Link to Github source code repository. The provided link allows access to the public Github repository with the source Python script file as well as the used definition files.
(PDF)

S3 File Zip file of raw representative mass spectra data. The included zip file contains text files with the data for the spectra used in the analysis discussed in this study.
(ZIP)
