# Supplementary material for: Development of a Python-based electron ionization mass spectrometry amino acid and peptide fragment prediction model
Source: PLoS One. 2024 Feb 16;19(2):e0297752. doi: 10.1371/journal.pone.0297752 (PMC10871511; doi:10.1371/journal.pone.0297752)
Supplement: S1 Table — (PDF) [file pone.0297752.s001.pdf]

Table S1: Crude Synthetic Dipeptides Used for Prediction Comparison.

**Peptide Sequence**	**M^+ Ion m/z**
AI	202.13
KI	259.19
SI	218.13
RI	287.20
LI	244.18
PA	186.10
CA	192.06
HA	226.11
YA	252.11
