# Supplementary figures and images for: Development of a Python-based electron ionization mass spectrometry amino acid and peptide fragment prediction model
Source: PLoS One. 2024 Feb 16;19(2):e0297752. doi: 10.1371/journal.pone.0297752 (PMC10871511; doi:10.1371/journal.pone.0297752)

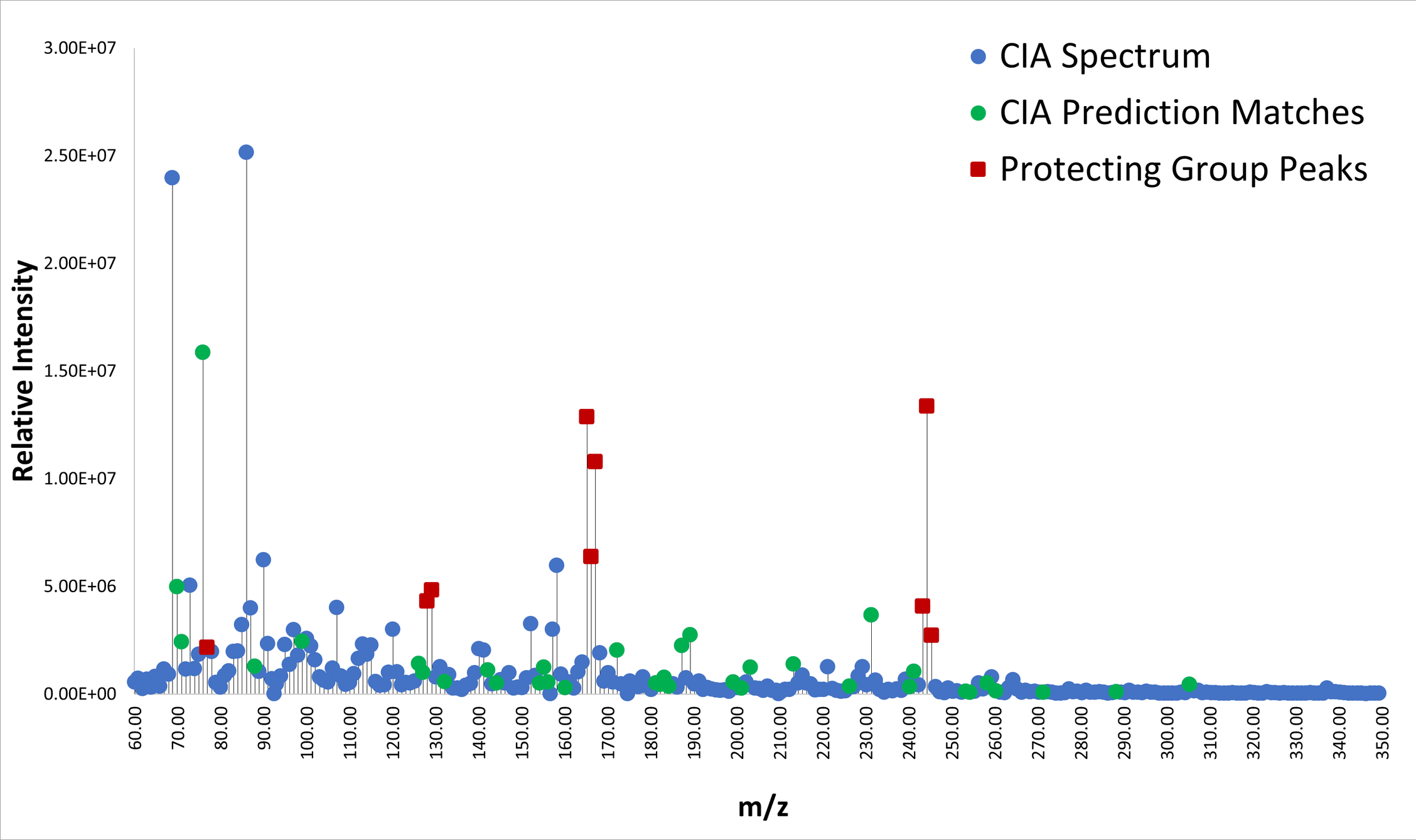

Supplement: S1 Fig — In blue are all plotted peaks observed in the spectrum for the CIA peptide. In green are peaks that match with predictions generated using our model. Red squares mark peaks associated with protecting groups used in the synthesis of the peptides. A peak was considered a match if it was within the max instrumental error (+/- 0.25 m/z) of the mass spectrometer. (TIF) [file pone.0297752.s002.tif]

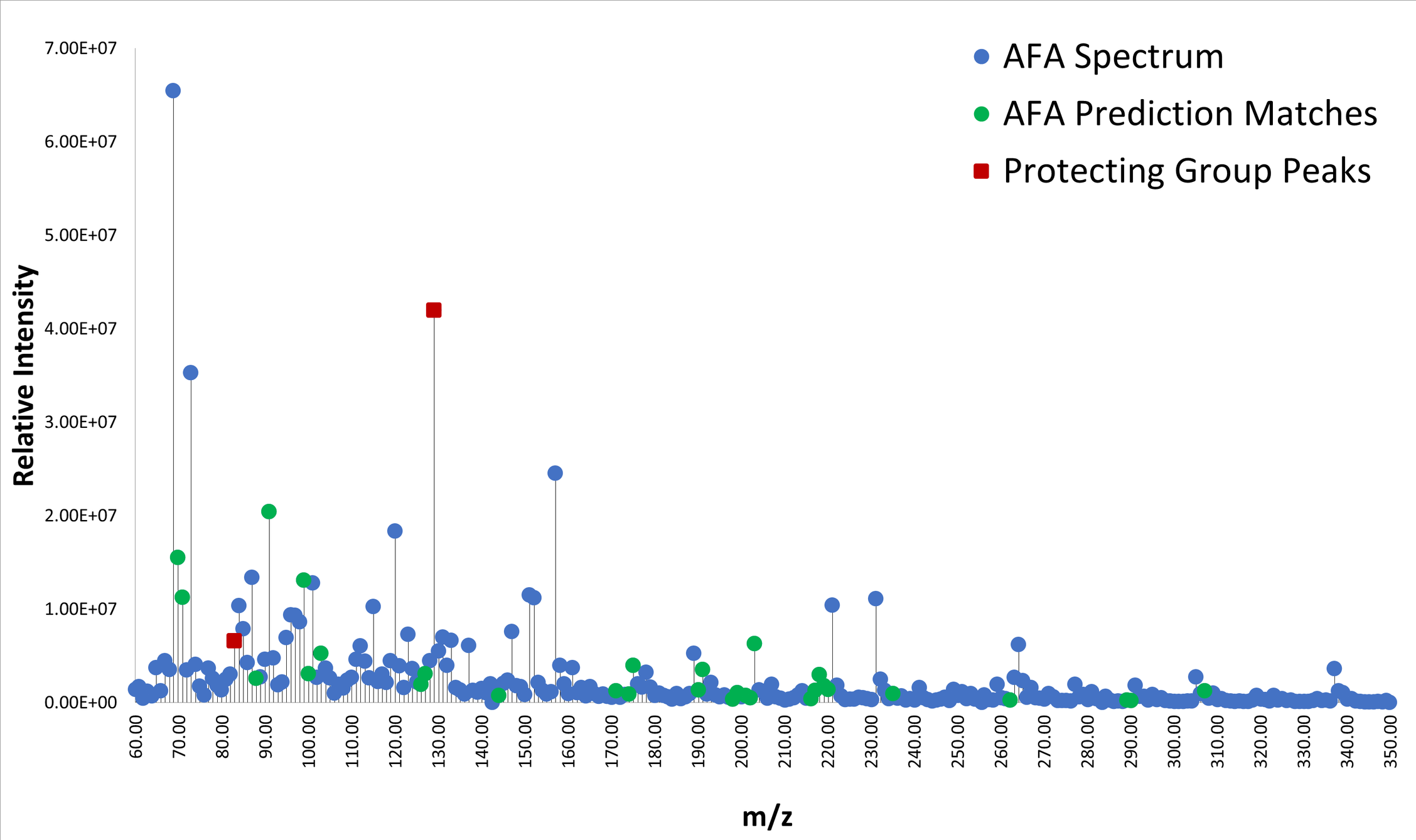

Supplement: S2 Fig — In blue are all plotted peaks observed in the spectrum for the AFA peptide. In green are peaks that match with predictions generated using our model. Red squares mark peaks associated with protecting groups used in the synthesis of the peptides. A peak was considered a match if it was within the max instrumental error (+/- 0.25 m/z) of the mass spectrometer. (TIF) [file pone.0297752.s003.tif]

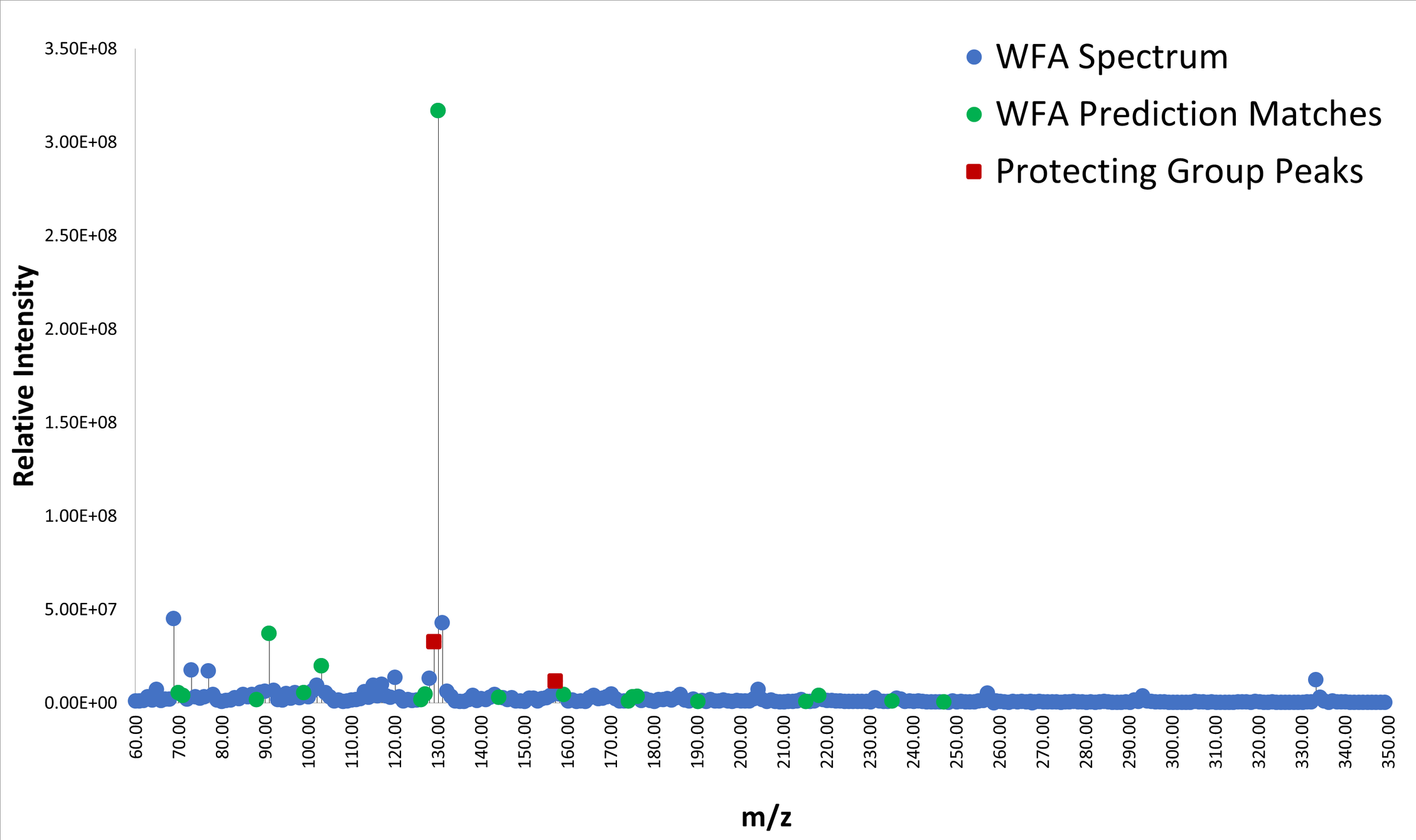

Supplement: S3 Fig — In blue are all plotted peaks observed in the spectrum for the WFA peptide. In green are peaks that match with predictions generated using our model. Red squares mark peaks associated with protecting groups used in the synthesis of the peptides. A peak was considered a match if it was within the max instrumental error (+/- 0.25 m/z) of the mass spectrometer. (TIF) [file pone.0297752.s004.tif]

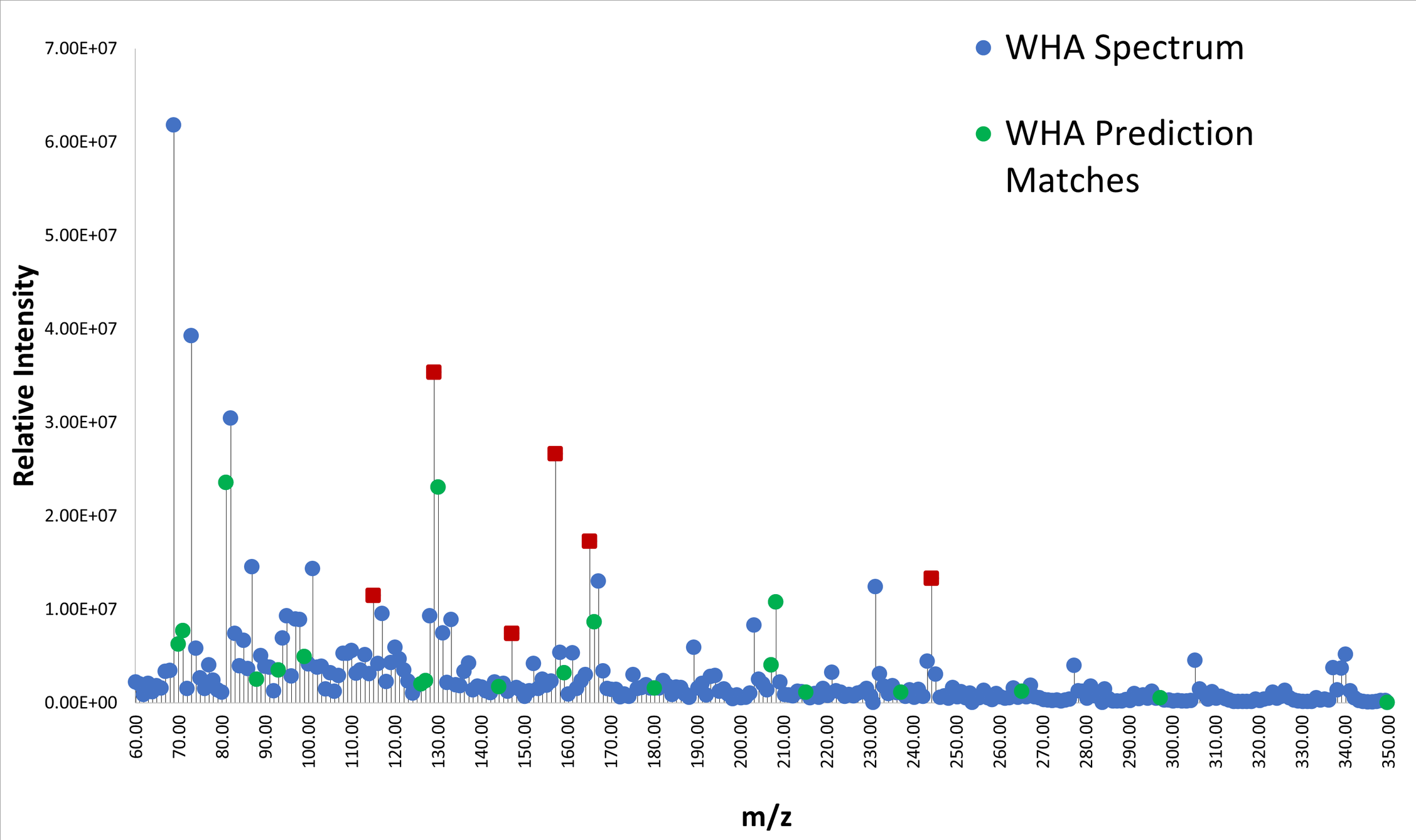

Supplement: S4 Fig — In blue are all plotted peaks observed in the spectrum for the WHA peptide. In green are peaks that match with predictions generated using our model. Red squares mark peaks associated with protecting groups used in the synthesis of the peptides. A peak was considered a match if it was within the max instrumental error (+/- 0.25 m/z) of the mass spectrometer. (TIF) [file pone.0297752.s005.tif]

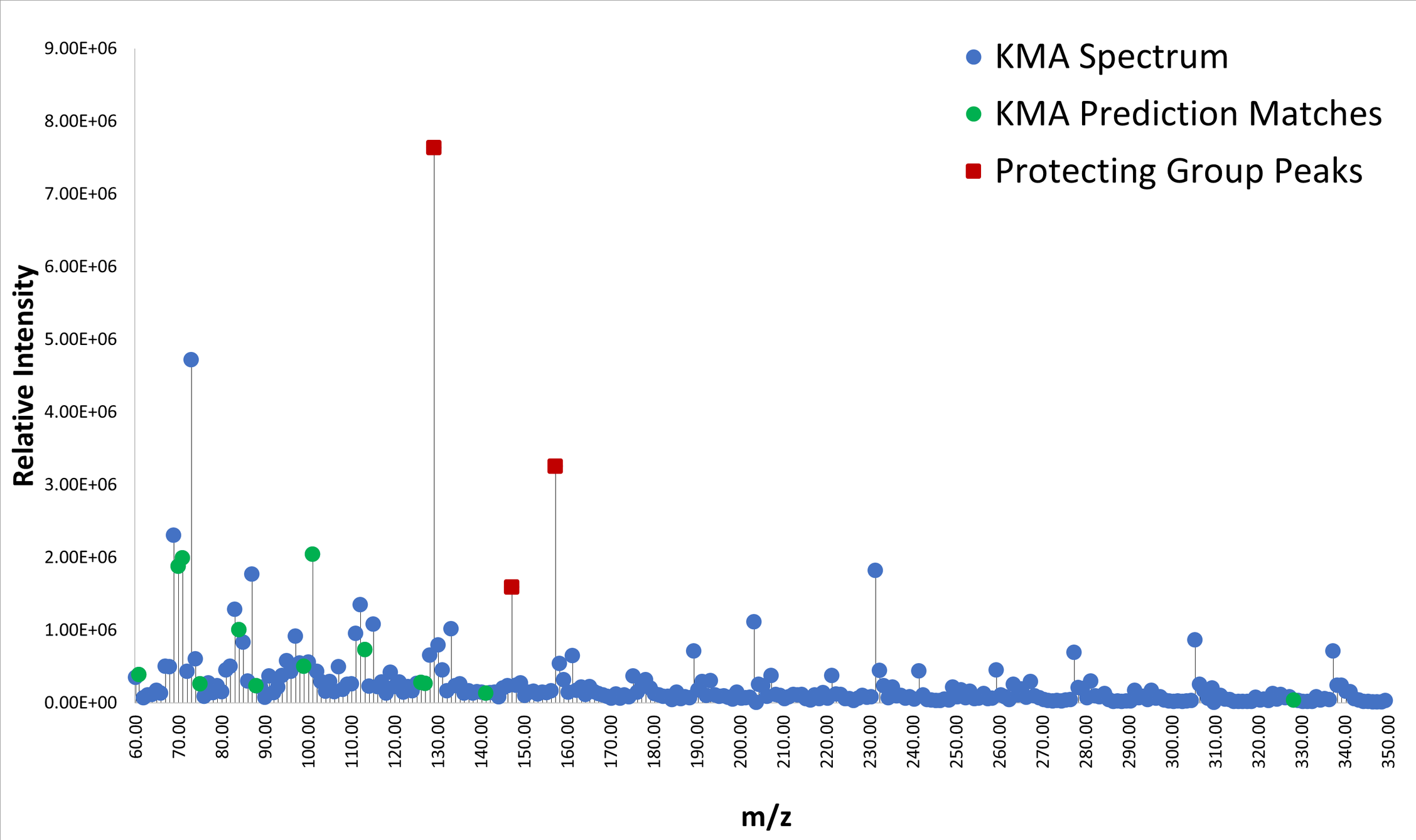

Supplement: S5 Fig — In blue are all plotted peaks observed in the spectrum for the KMA peptide. In green are peaks that match with predictions generated using our model. Red squares mark peaks associated with protecting groups used in the synthesis of the peptides. A peak was considered a match if it was within the max instrumental error (+/- 0.25 m/z) of the mass spectrometer. (TIF) [file pone.0297752.s006.tif]

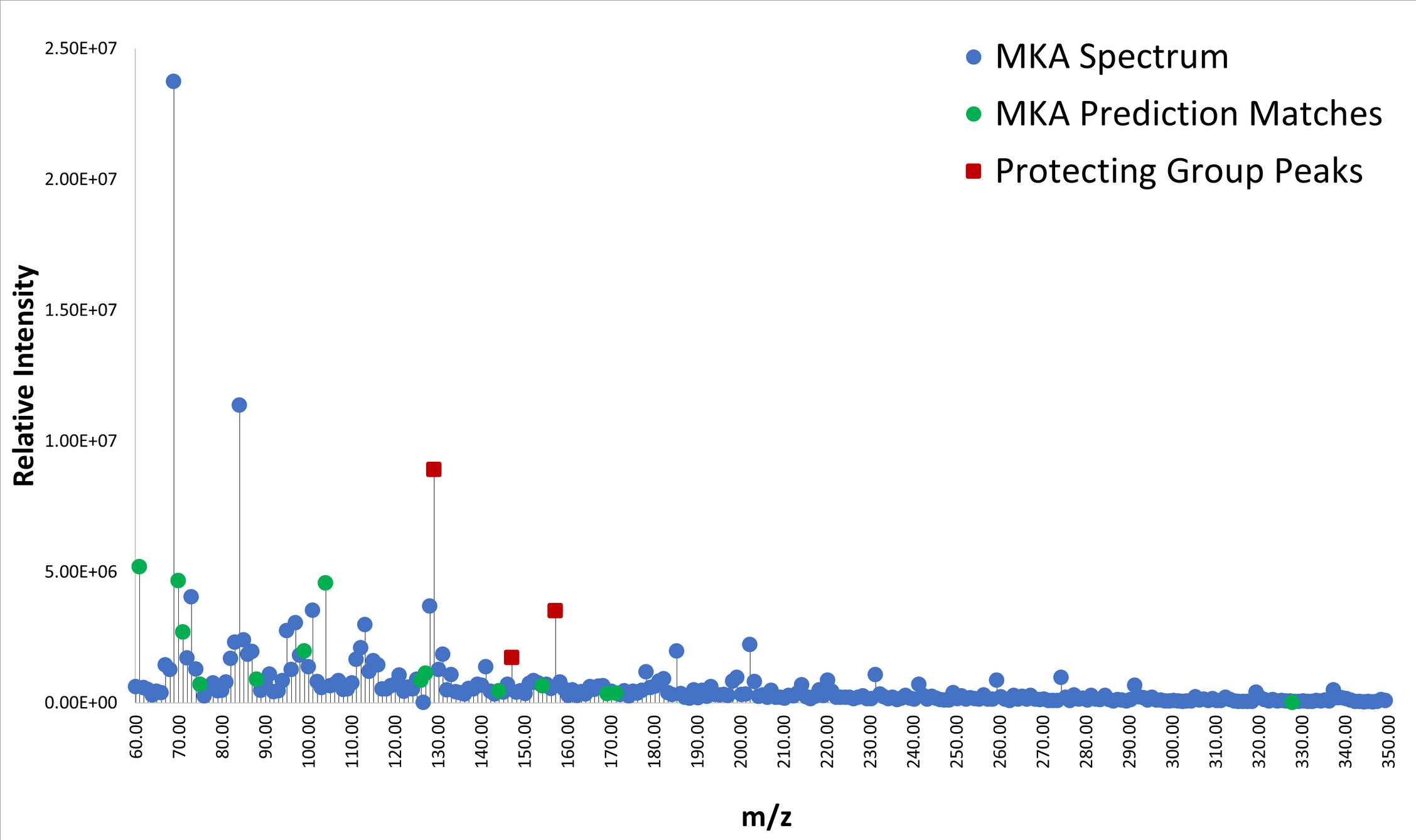

Supplement: S6 Fig — In blue are all plotted peaks observed in the spectrum for the MKA peptide. In green are peaks that match with predictions generated using our model. Red squares mark peaks associated with protecting groups used in the synthesis of the peptides. A peak was considered a match if it was within the max instrumental error (+/- 0.25 m/z) of the mass spectrometer. (TIF) [file pone.0297752.s007.tif]

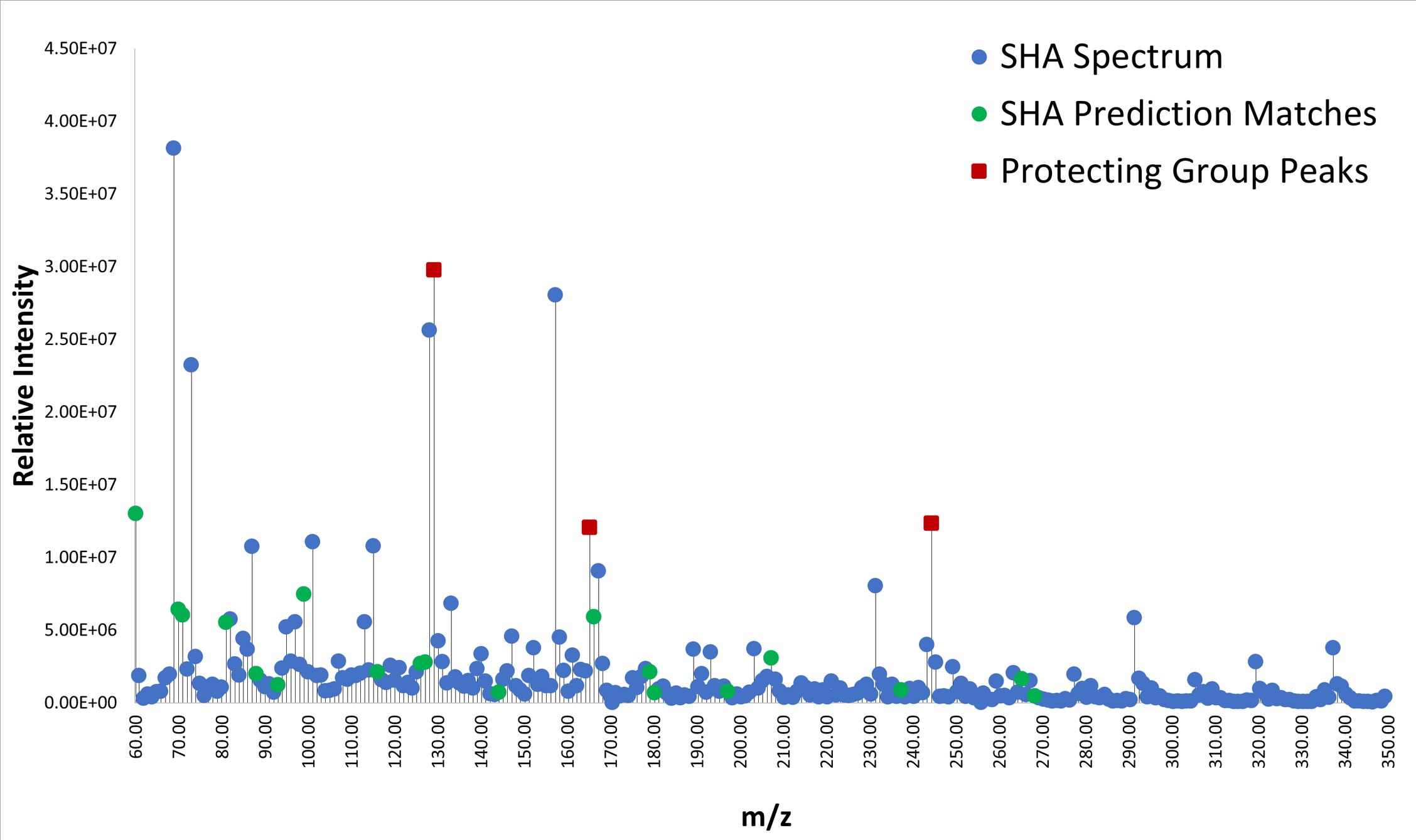

Supplement: S7 Fig — In blue are all plotted peaks observed in the spectrum for the SHA peptide. In green are peaks that match with predictions generated using our model. Red squares mark peaks associated with protecting groups used in the synthesis of the peptides. A peak was considered a match if it was within the max instrumental error (+/- 0.25 m/z) of the mass spectrometer. (TIF) [file pone.0297752.s008.tif]

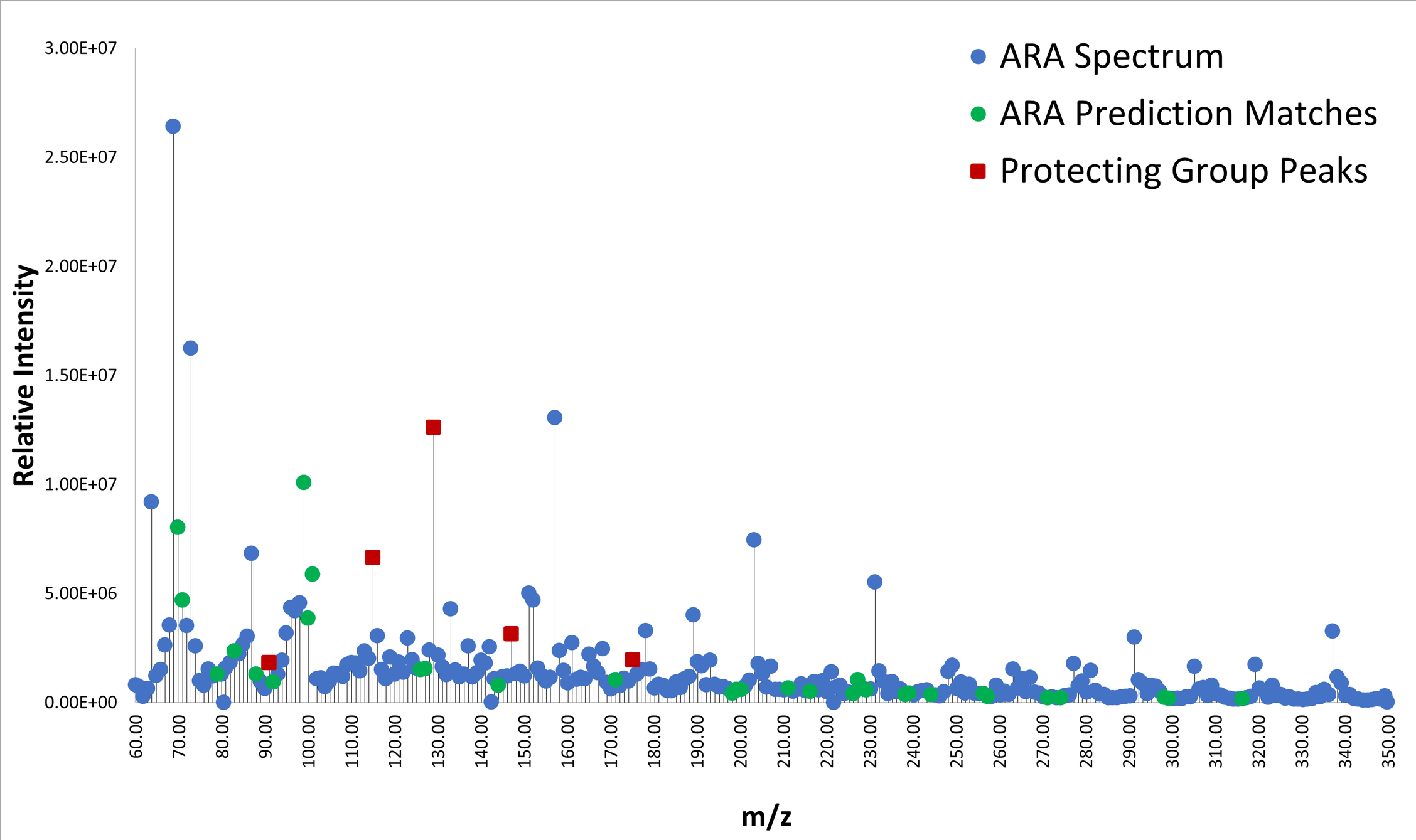

Supplement: S8 Fig — In blue are all plotted peaks observed in the spectrum for the ARA peptide. In green are peaks that match with predictions generated using our model. Red squares mark peaks associated with protecting groups used in the synthesis of the peptides. A peak was considered a match if it was within the max instrumental error (+/- 0.25 m/z) of the mass spectrometer. (TIF) [file pone.0297752.s009.tif]

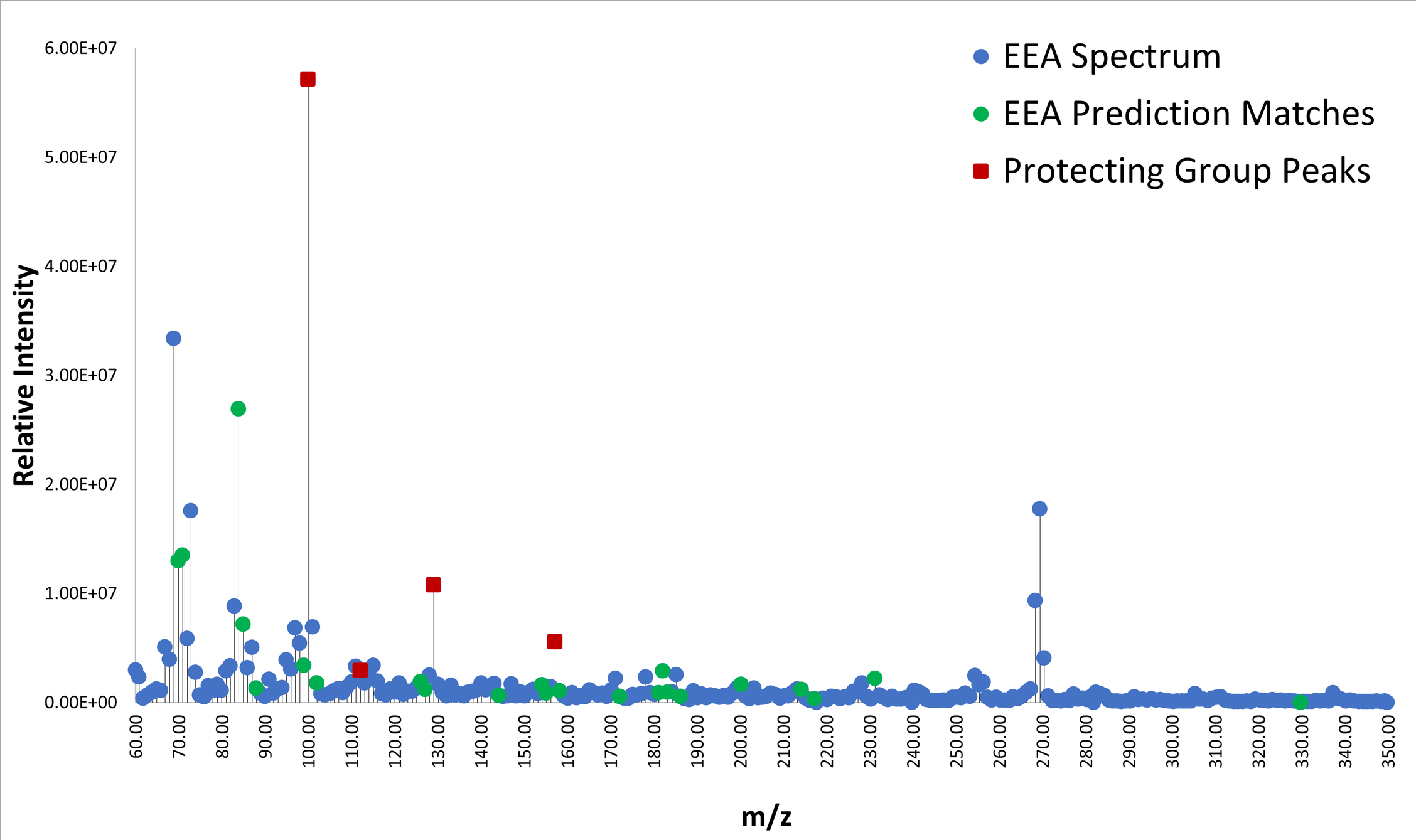

Supplement: S9 Fig — In blue are all plotted peaks observed in the spectrum for the EEA peptide. In green are peaks that match with predictions generated using our model. Red squares mark peaks associated with protecting groups used in the synthesis of the peptides. A peak was considered a match if it was within the max instrumental error (+/- 0.25 m/z) of the mass spectrometer. (TIF) [file pone.0297752.s010.tif]

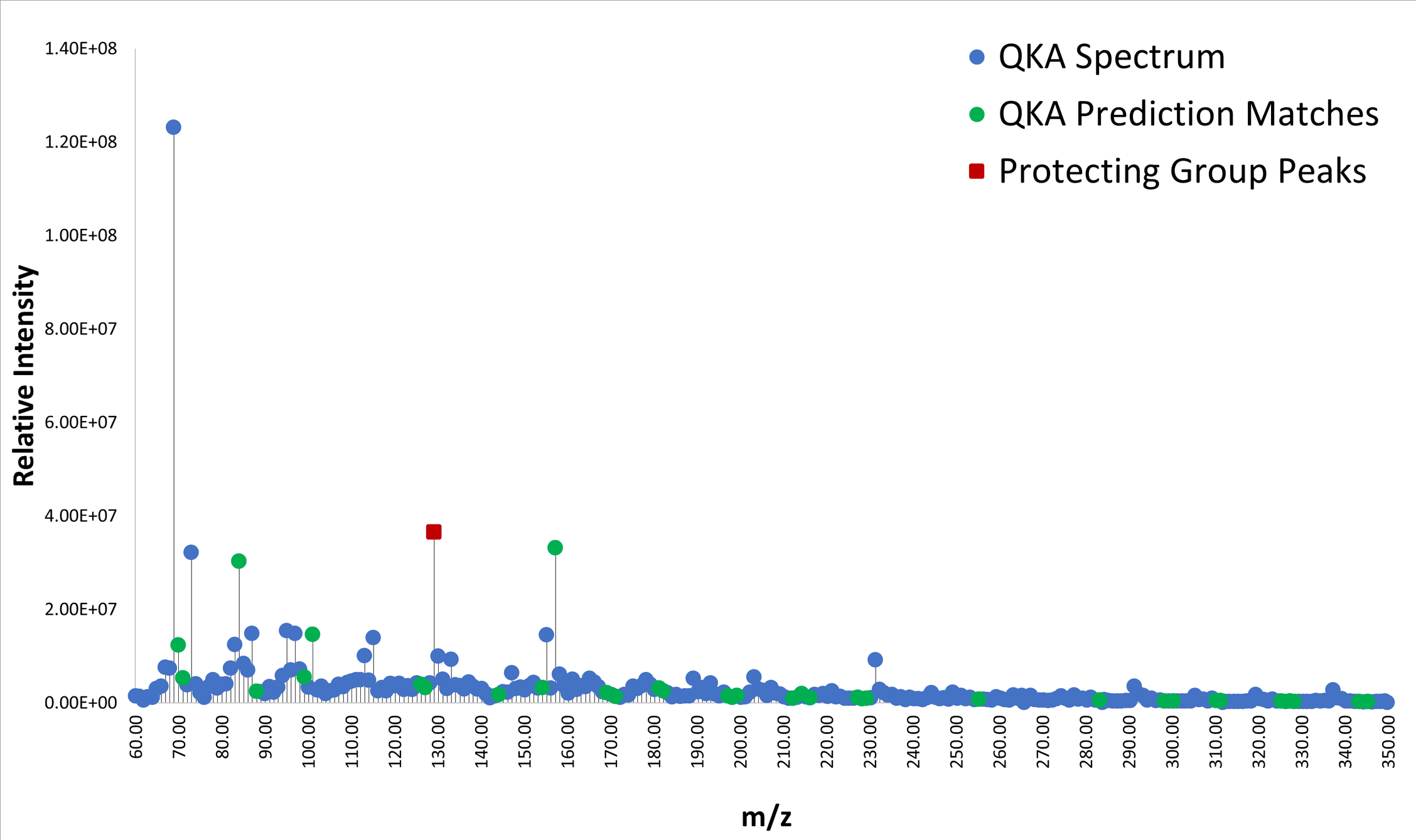

Supplement: S10 Fig — In blue are all plotted peaks observed in the spectrum for the QKA peptide. In green are peaks that match with predictions generated using our model. Red squares mark peaks associated with protecting groups used in the synthesis of the peptides. A peak was considered a match if it was within the max instrumental error (+/- 0.25 m/z) of the mass spectrometer. (TIF) [file pone.0297752.s011.tif]

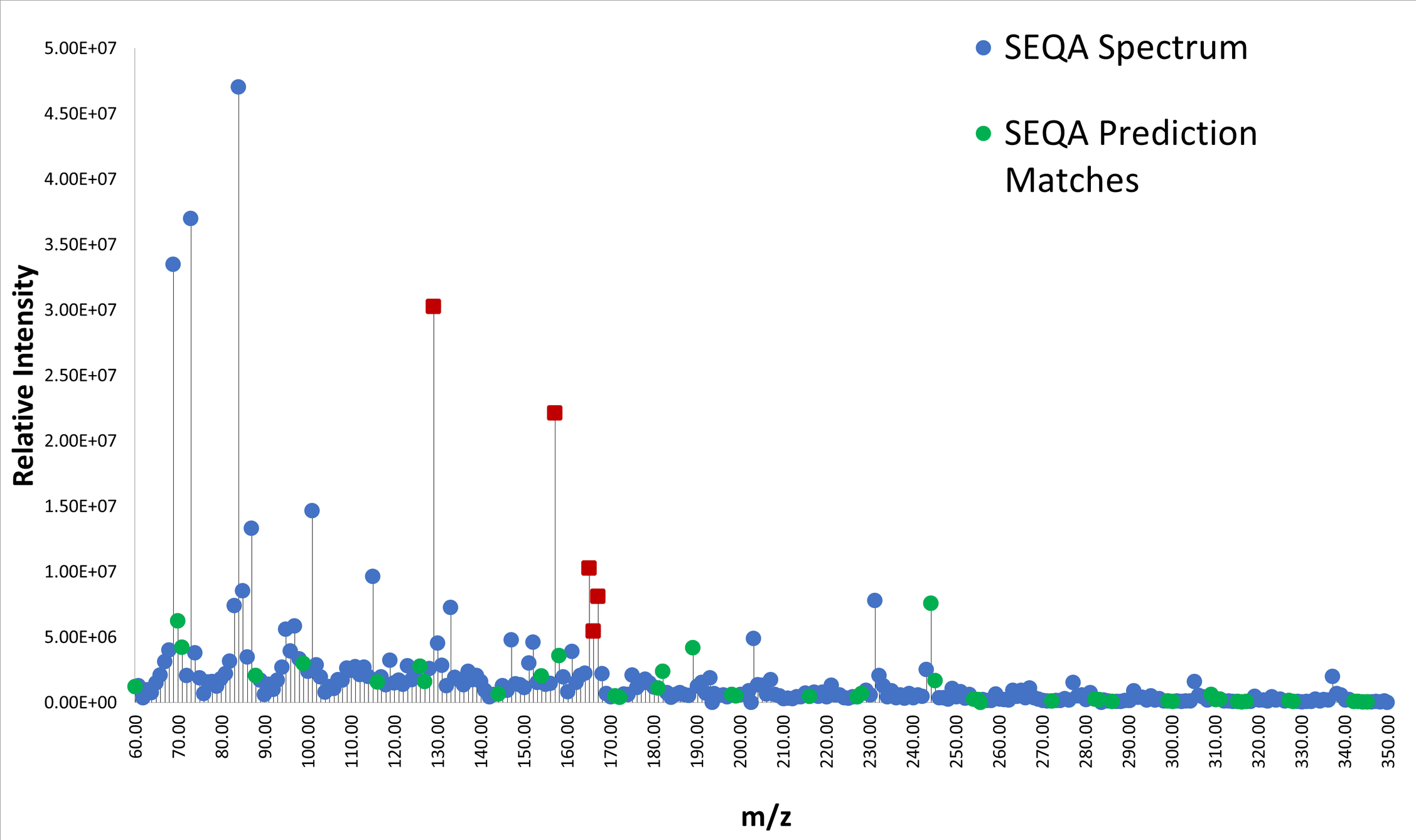

Supplement: S11 Fig — In blue are all plotted peaks observed in the spectrum for the SEQA peptide. In green are peaks that match with predictions generated using our model. Red squares mark peaks associated with protecting groups used in the synthesis of the peptides. A peak was considered a match if it was within the max instrumental error (+/- 0.25 m/z) of the mass spectrometer. (TIF) [file pone.0297752.s012.tif]

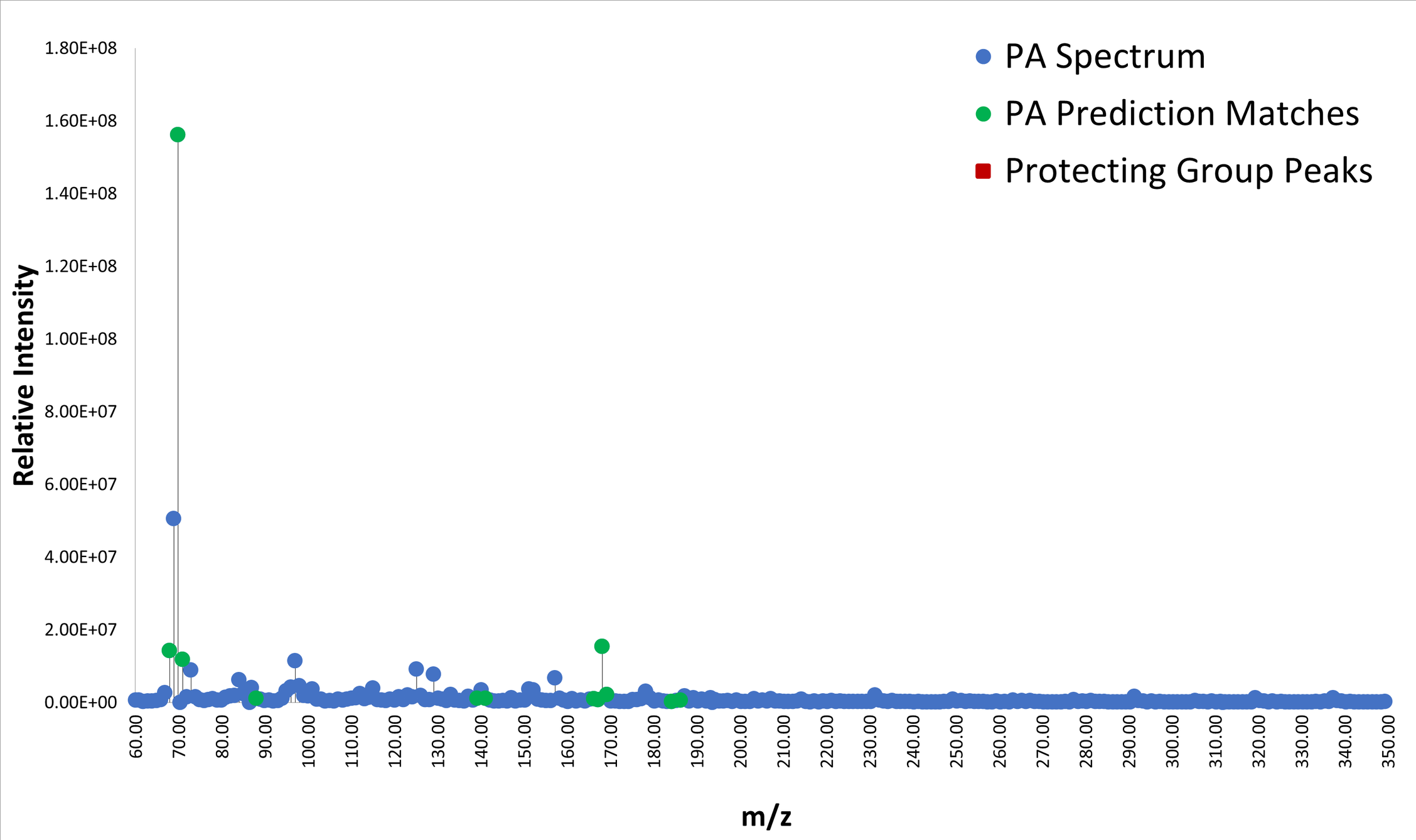

Supplement: S12 Fig — In blue are all plotted peaks observed in the spectrum for the PA peptide. In green are peaks that match with predictions generated using our model. Red squares mark peaks associated with protecting groups used in the synthesis of the peptides. A peak was considered a match if it was within the max instrumental error (+/- 0.25 m/z) of the mass spectrometer. (TIF) [file pone.0297752.s013.tif]

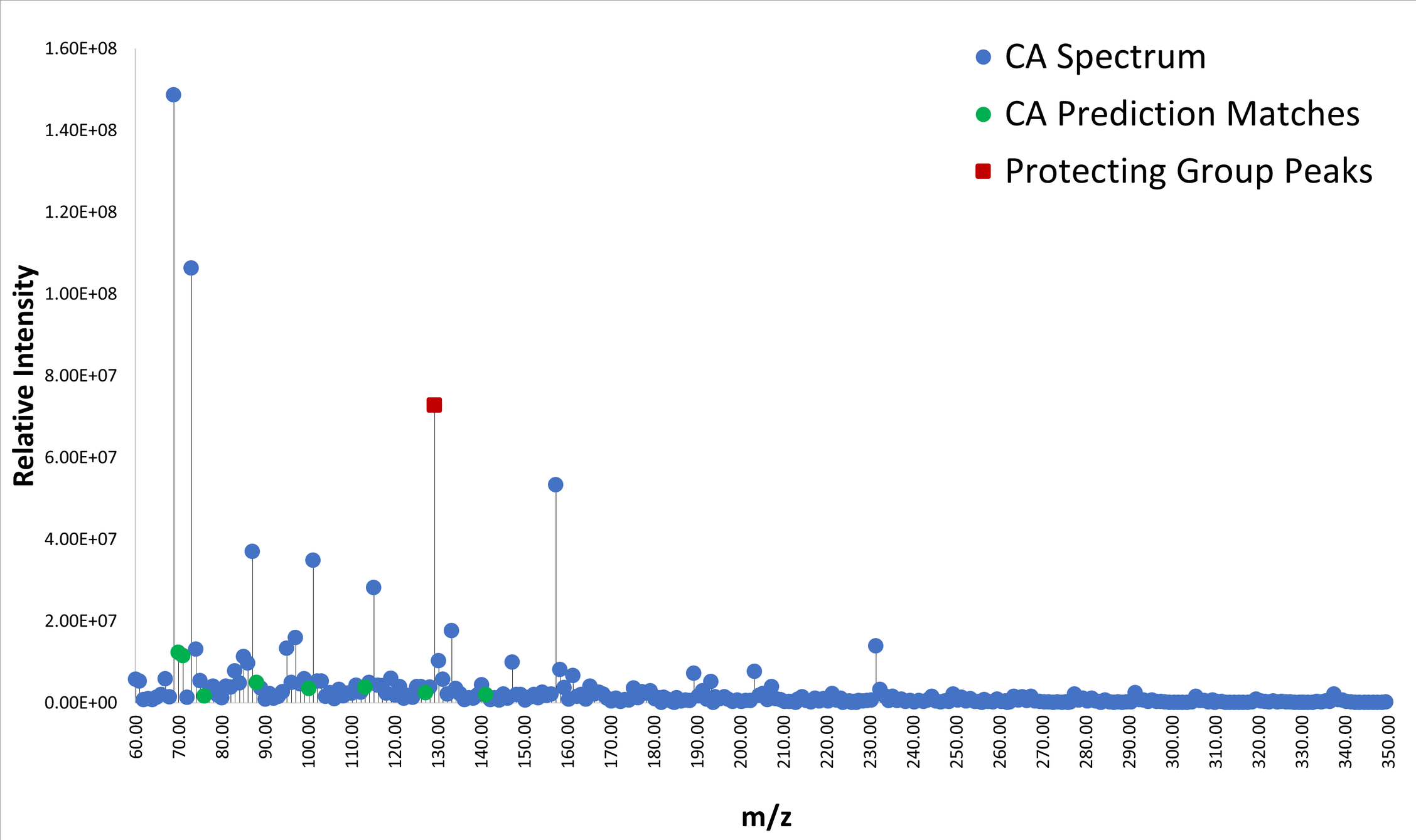

Supplement: S13 Fig — In blue are all plotted peaks observed in the spectrum for the CA peptide. In green are peaks that match with predictions generated using our model. Red squares mark peaks associated with protecting groups used in the synthesis of the peptides. A peak was considered a match if it was within the max instrumental error (+/- 0.25 m/z) of the mass spectrometer. (TIF) [file pone.0297752.s014.tif]

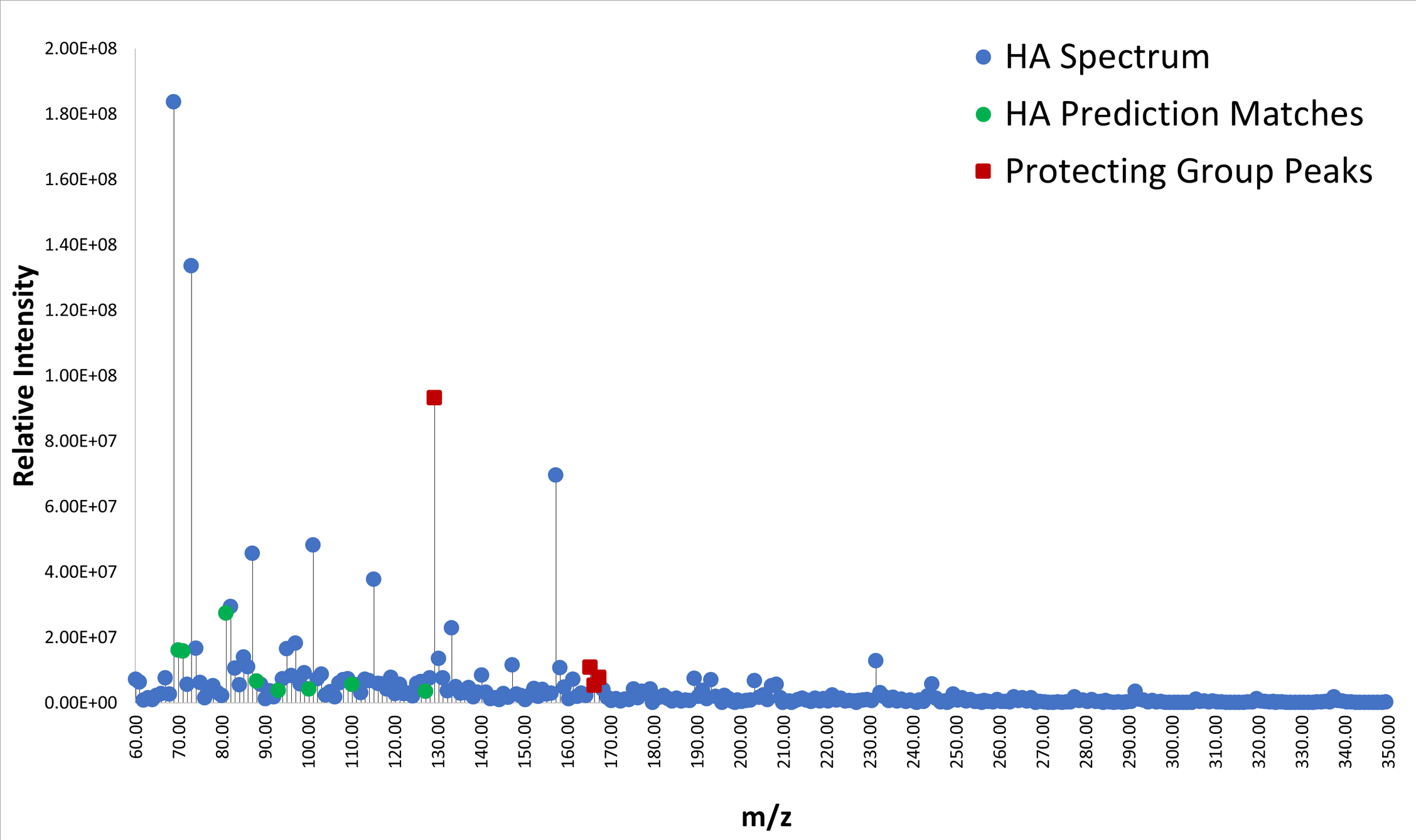

Supplement: S14 Fig — In blue are all plotted peaks observed in the spectrum for the HA peptide. In green are peaks that match with predictions generated using our model. Red squares mark peaks associated with protecting groups used in the synthesis of the peptides. A peak was considered a match if it was within the max instrumental error (+/- 0.25 m/z) of the mass spectrometer. (TIF) [file pone.0297752.s015.tif]

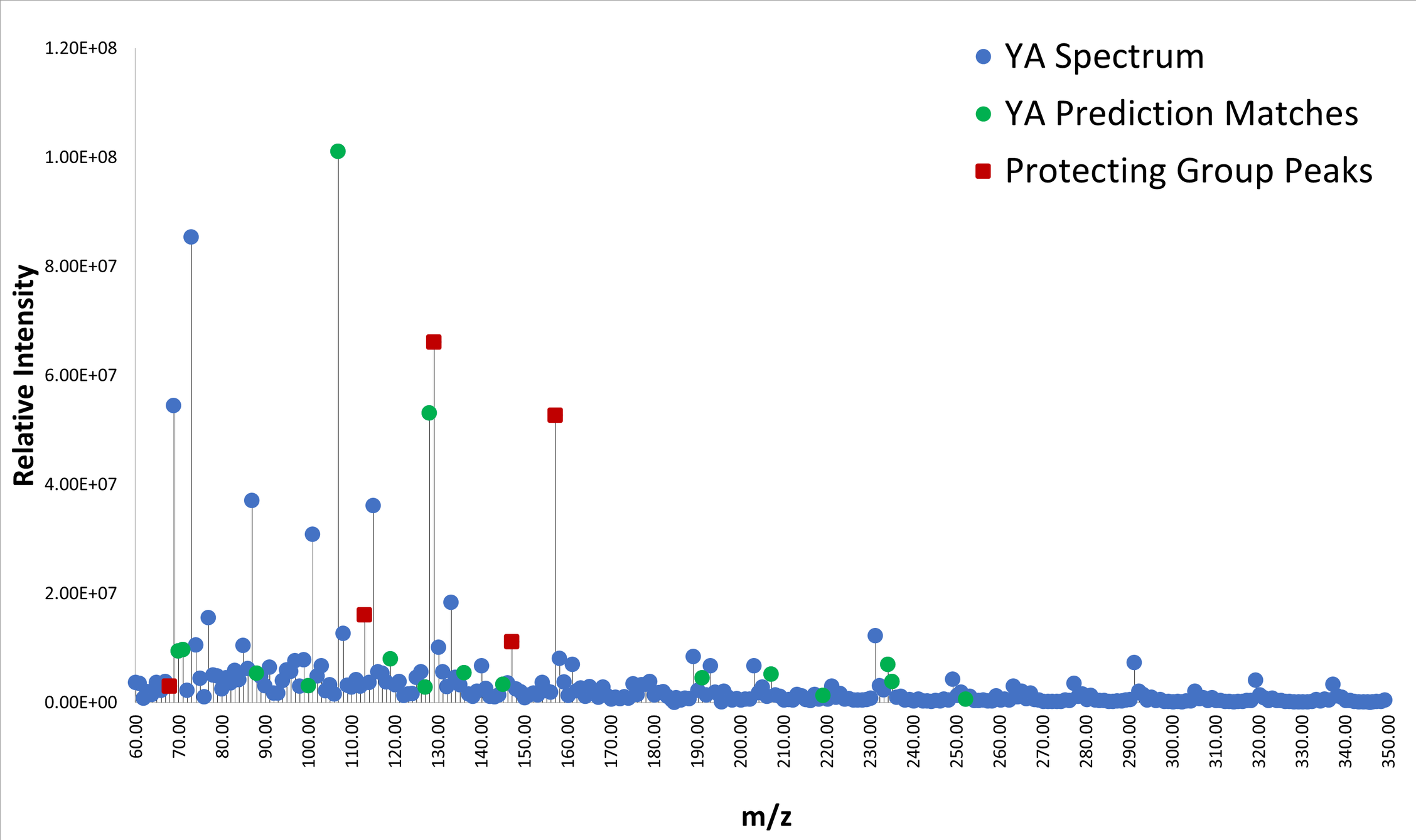

Supplement: S15 Fig — In blue are all plotted peaks observed in the spectrum for the YA peptide. In green are peaks that match with predictions generated using our model. Red squares mark peaks associated with protecting groups used in the synthesis of the peptides. A peak was considered a match if it was within the max instrumental error (+/- 0.25 m/z) of the mass spectrometer. (TIF) [file pone.0297752.s016.tif]

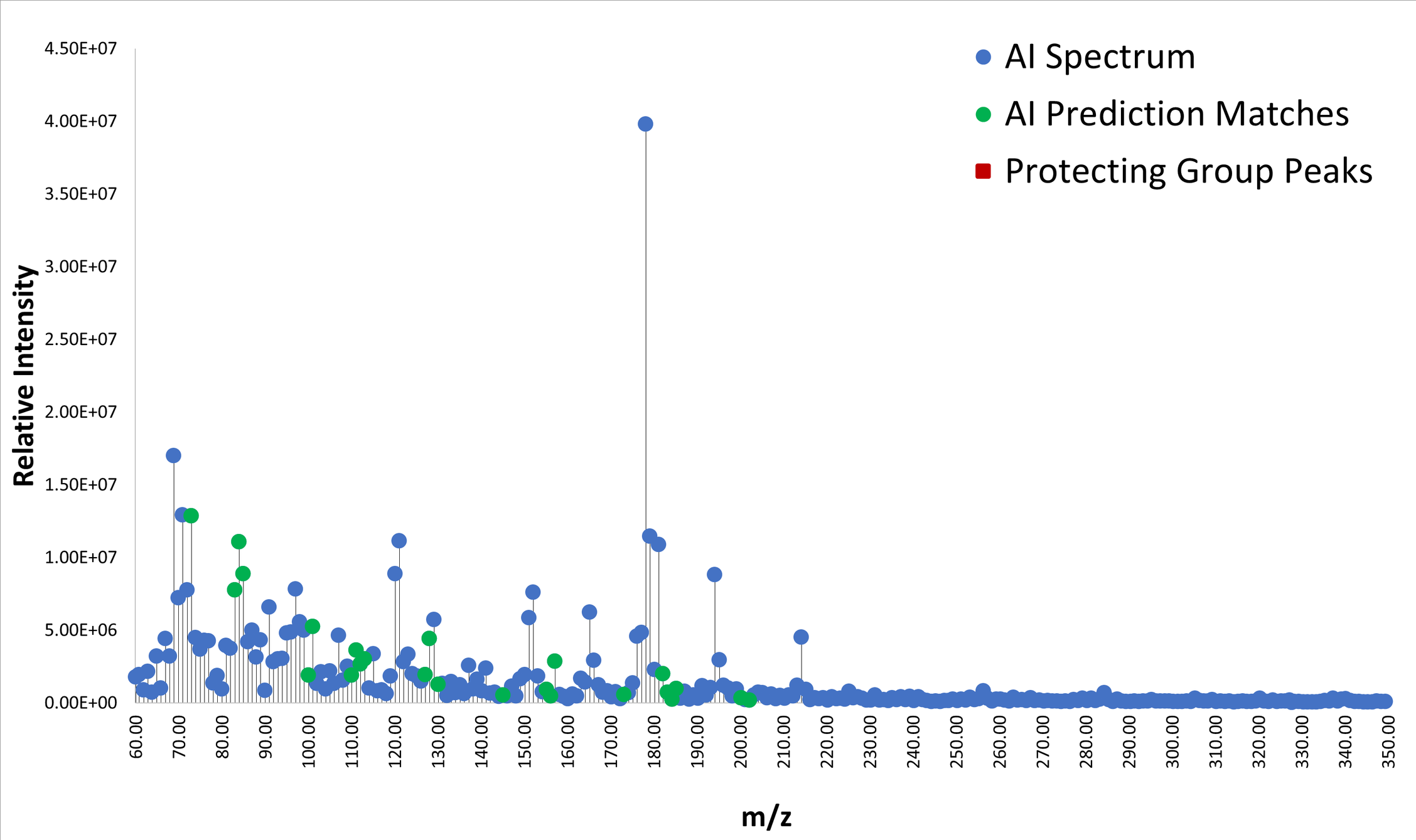

Supplement: S16 Fig — In blue are all plotted peaks observed in the spectrum for the AI peptide. In green are peaks that match with predictions generated using our model. Red squares mark peaks associated with protecting groups used in the synthesis of the peptides. A peak was considered a match if it was within the max instrumental error (+/- 0.25 m/z) of the mass spectrometer. (TIF) [file pone.0297752.s017.tif]

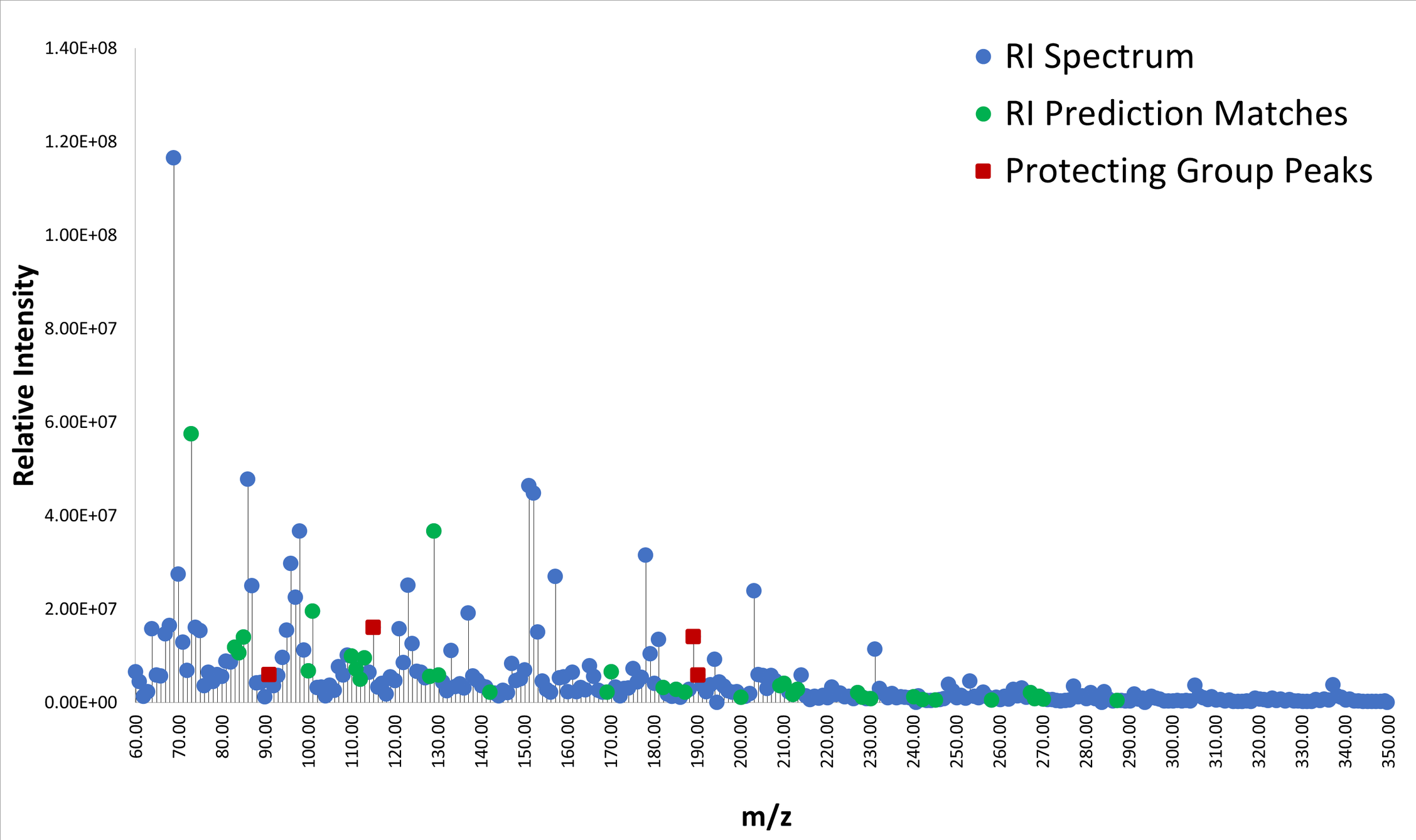

Supplement: S17 Fig — In blue are all plotted peaks observed in the spectrum for the RI peptide. In green are peaks that match with predictions generated using our model. Red squares mark peaks associated with protecting groups used in the synthesis of the peptides. A peak was considered a match if it was within the max instrumental error (+/- 0.25 m/z) of the mass spectrometer. (TIF) [file pone.0297752.s018.tif]

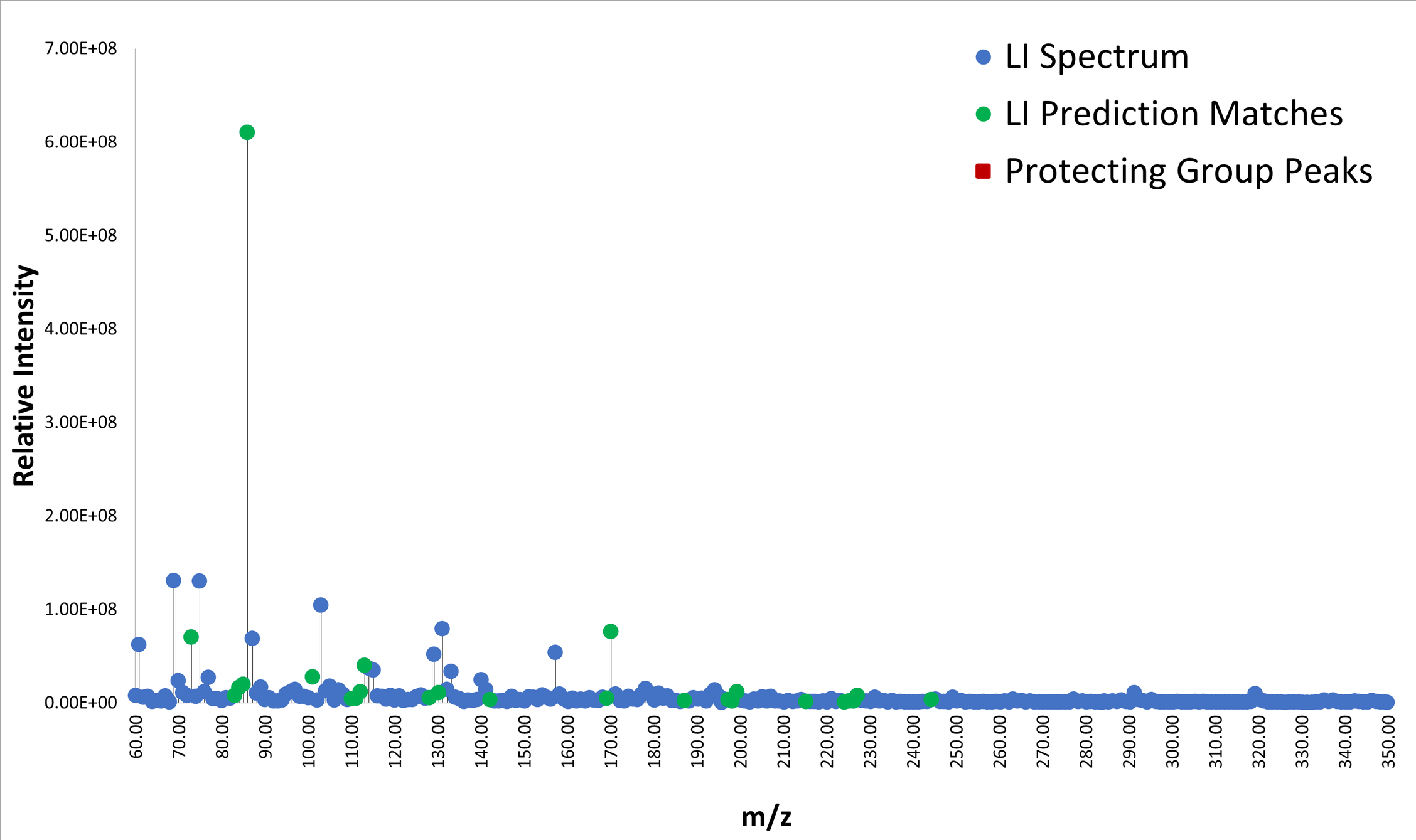

Supplement: S18 Fig — In blue are all plotted peaks observed in the spectrum for the LI peptide. In green are peaks that match with predictions generated using our model. Red squares mark peaks associated with protecting groups used in the synthesis of the peptides. A peak was considered a match if it was within the max instrumental error (+/- 0.25 m/z) of the mass spectrometer. (TIF) [file pone.0297752.s019.tif]

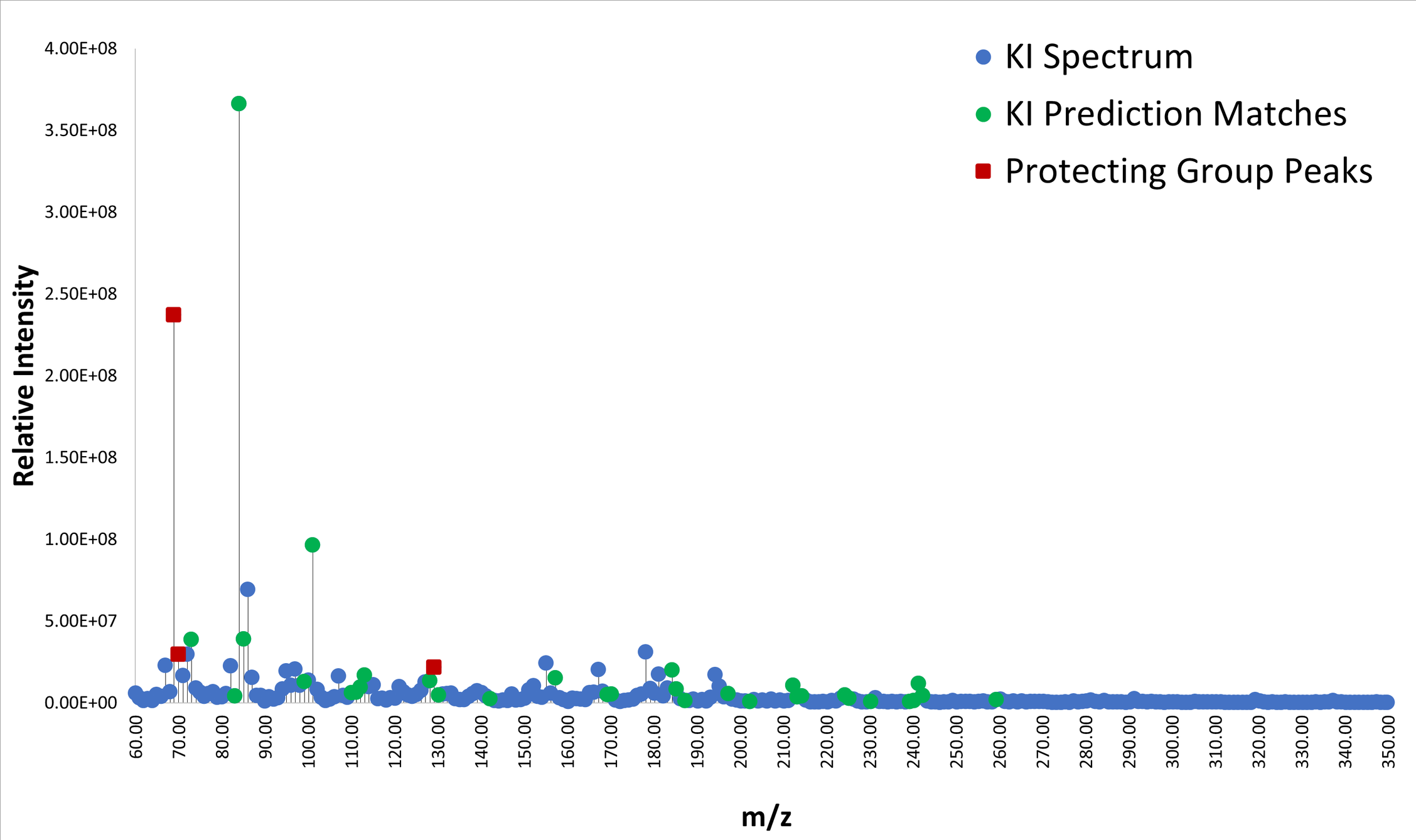

Supplement: S19 Fig — In blue are all plotted peaks observed in the spectrum for the KI peptide. In green are peaks that match with predictions generated using our model. Red squares mark peaks associated with protecting groups used in the synthesis of the peptides. A peak was considered a match if it was within the max instrumental error (+/- 0.25 m/z) of the mass spectrometer. (TIF) [file pone.0297752.s020.tif]

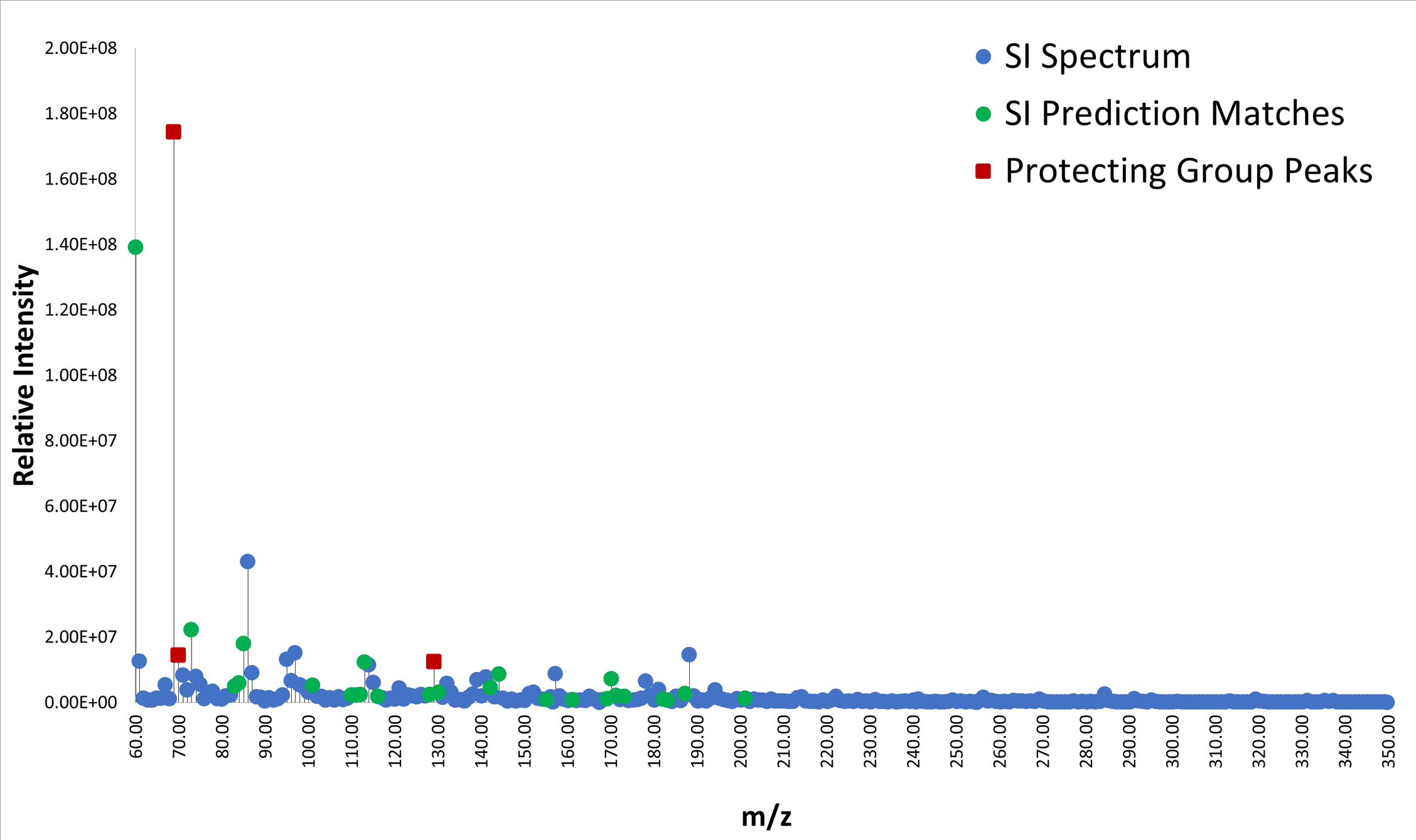

Supplement: S20 Fig — In blue are all plotted peaks observed in the spectrum for the SI peptide. In green are peaks that match with predictions generated using our model. Red squares mark peaks associated with protecting groups used in the synthesis of the peptides. A peak was considered a match if it was within the max instrumental error (+/- 0.25 m/z) of the mass spectrometer. (TIF) [file pone.0297752.s021.tif]

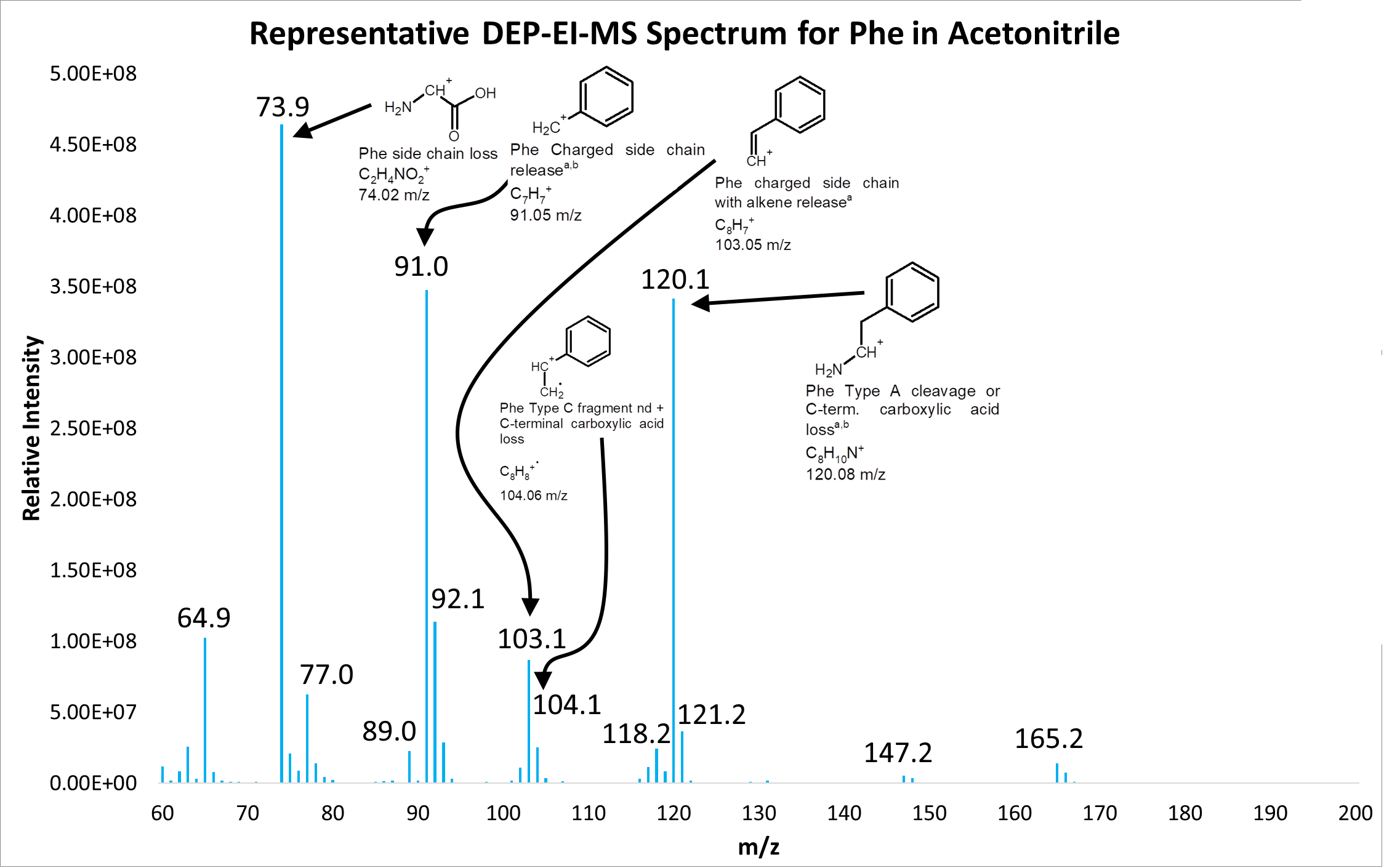

Supplement: S21 Fig — Several fragmentation mechanisms are present in the Phe spectrum. This includes two non-relative fragmentation mechanisms where the observed peak corresponds to the released side chain (with or without the apparent formation of an alkene). Beyond the common loss of side chain mechanism shared by all of the amino acids, two relative fragmentation mechanisms include Type A-like cleavage or the loss of the C-terminal carboxylic acid group and a Type C cleavage without formation of an alkene (nd). The maximum peak intensity for the shown spectrum is 4.64 X 108 counts. Proposed structures are shown along with the resulting fragment formula and monoisotopic m/z. adegradation type also observed in [11], bdegradation type also observed in [3]. (TIF) [file pone.0297752.s022.tif]

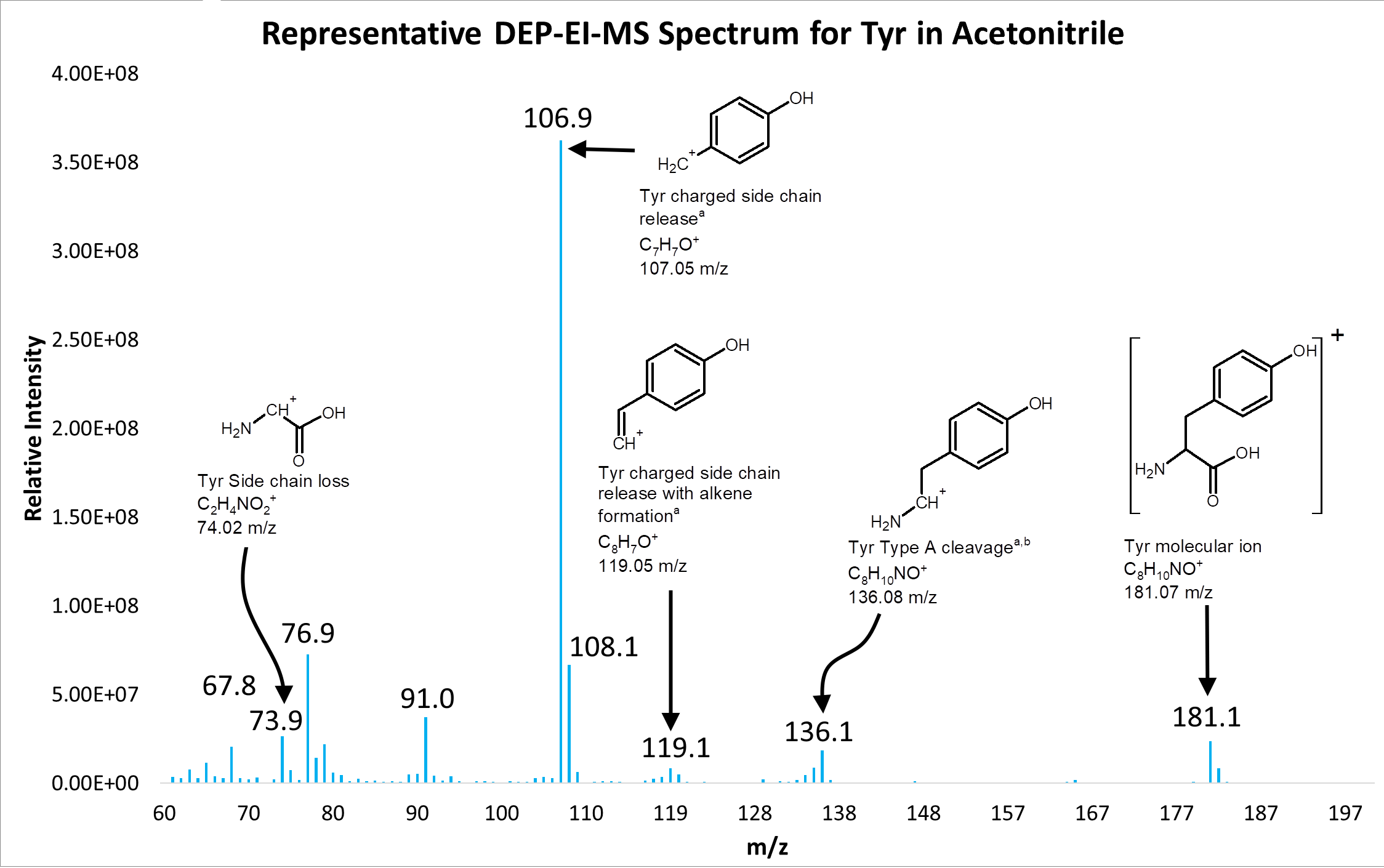

Supplement: S22 Fig — Several fragmentation mechanisms are present in the Tyr spectrum. Similar to Phe, there are two non-relative fragmentation mechanisms where the observed peak corresponds to the released side chain (with or without the apparent formation of an alkene). The common loss of side chain mechanism is present as well as a peak for the molecular (non-fragmented) ion. Type A-like cleavage or the loss of the C-terminal carboxylic acid group composes the last of the identified fragmentation mechanisms. The maximum peak intensity for the shown spectrum is 3.62 X 108 counts. Proposed structures are shown along with the resulting fragment formula and monoisotopic m/z. adegradation type also observed in [11], bdegradation type also observed in [3]. (TIF) [file pone.0297752.s023.tif]

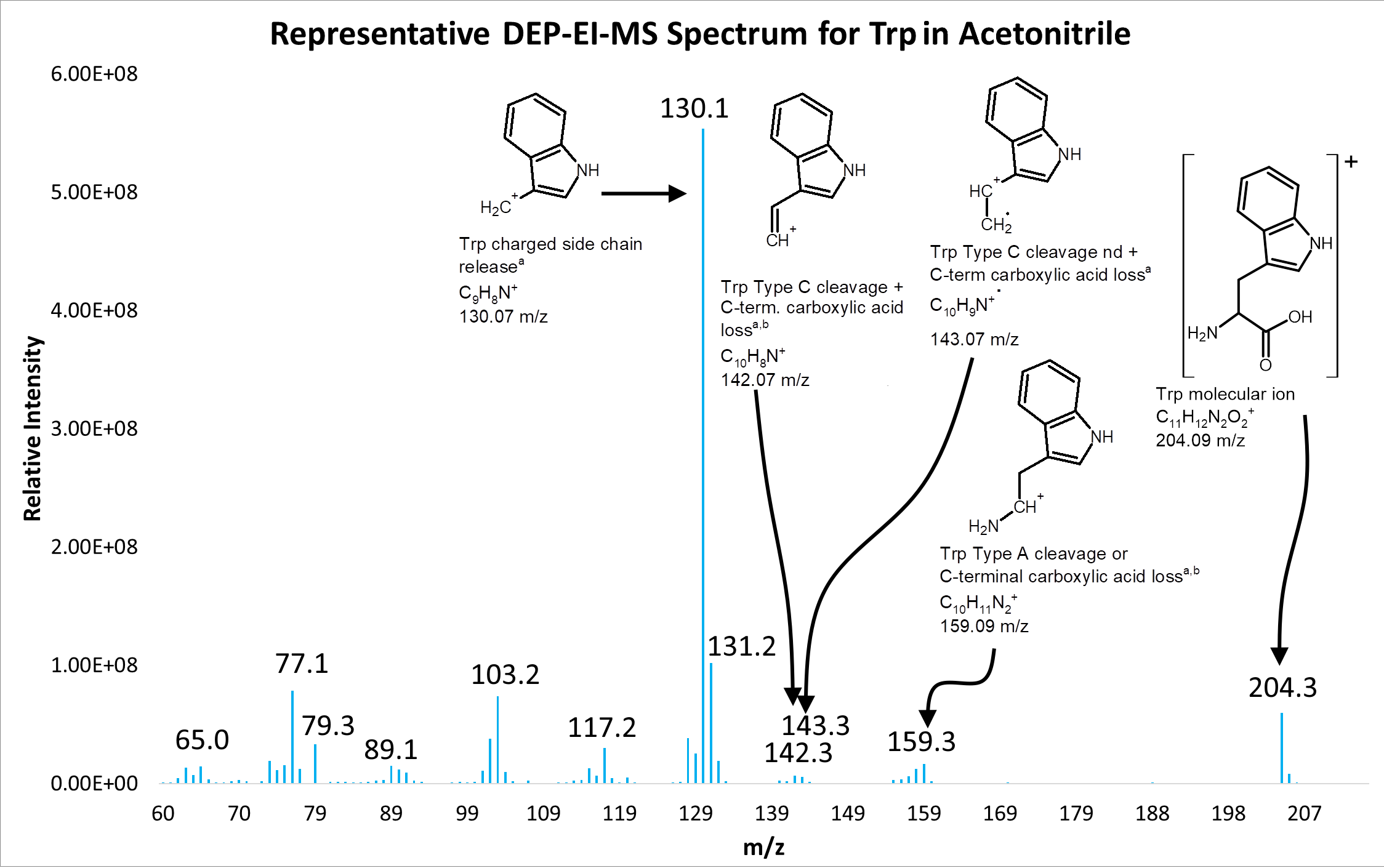

Supplement: S23 Fig — Several fragmentation mechanisms are present in the Trp spectrum. Only one non-relative fragmentation mechanism was observed, appearing to release the charged side chain similar to the other conjugated amino acids. The peak corresponding to the loss of the side chain was present, but at a much lower intensity than for most of the other amino acids. The molecular ion was observed along with a Type A-like cleavage or the loss of the C-terminal carboxylic acid group. The remaining mechanisms involved Type-C cleavage with and without alkene formation. The maximum peak intensity for the shown spectrum is 5.54 X 108 counts. Proposed structures are shown along with the resulting fragment formula and monoisotopic m/z. adegradation type also observed in [11], bdegradation type also observed in [3]. (TIF) [file pone.0297752.s024.tif]

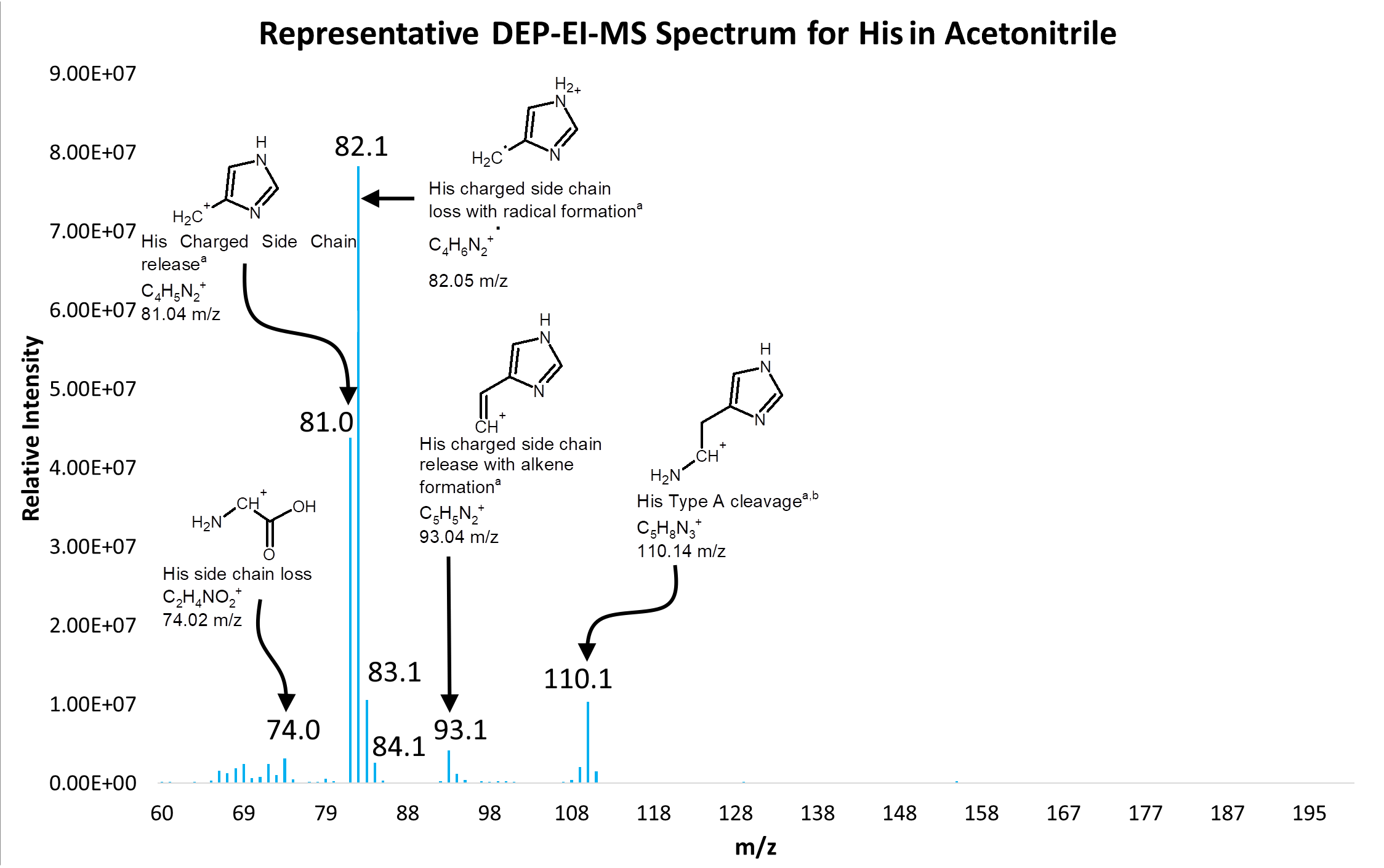

Supplement: S24 Fig — Several fragmentation mechanisms are present in the His spectrum. Similar to Phe and Tyr, there are two non-relative fragmentation mechanisms where the observed peak corresponds to the released side chain (with or without the apparent formation of an alkene). In addition, a third non-relative mechanism involves release of a protonated side chain with radical formation. Type A-like cleavage or the loss of the C-terminal carboxylic acid group composes the last of the identified fragmentation mechanisms. The maximum peak intensity for the shown spectrum is 7.83 X 107 counts. Proposed structures are shown along with the resulting fragment formula and monoisotopic m/z. adegradation type also observed in [11], bdegradation type also observed in [3]. (TIF) [file pone.0297752.s025.tif]

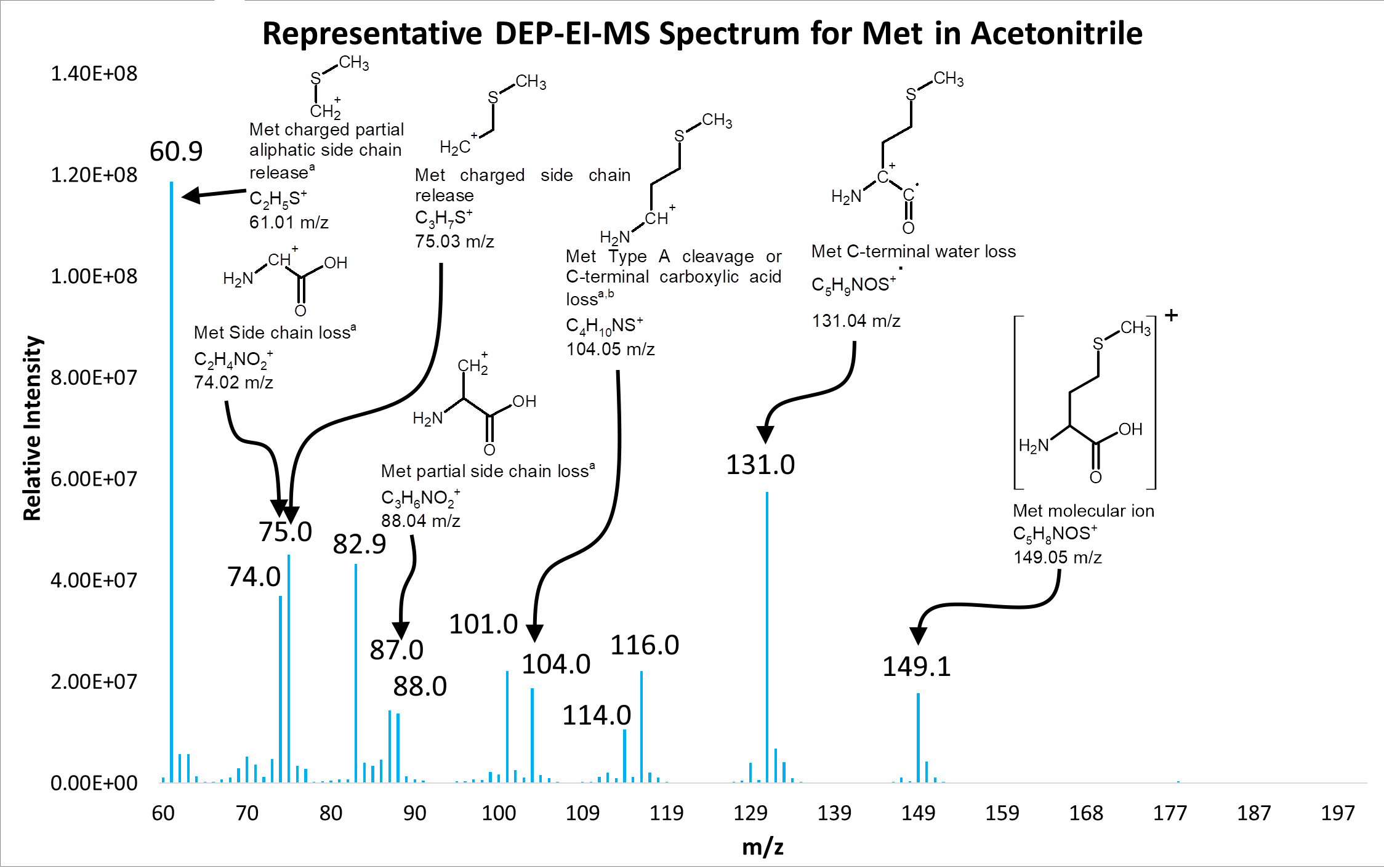

Supplement: S25 Fig — Several fragmentation mechanisms are present in the Met spectrum. Two non-relative fragmentation mechanisms were observed, releasing different charged sub-fragments of the side chain. The common full loss of side chain mechanism was observed as well as a partial loss of side chain. Type A-like cleavage or the loss of the C-terminal carboxylic acid group was observed along with the C-terminal loss of water. A peak corresponding to the molecular ion was also observed at a relatively high intensity. The maximum peak intensity for the shown spectrum is 1.19 X 108 counts. Proposed structures shown along with the resulting fragment formula and monoisotopic m/z. adegradation type also observed in [11], bdegradation type also observed in [3]. (TIF) [file pone.0297752.s026.tif]

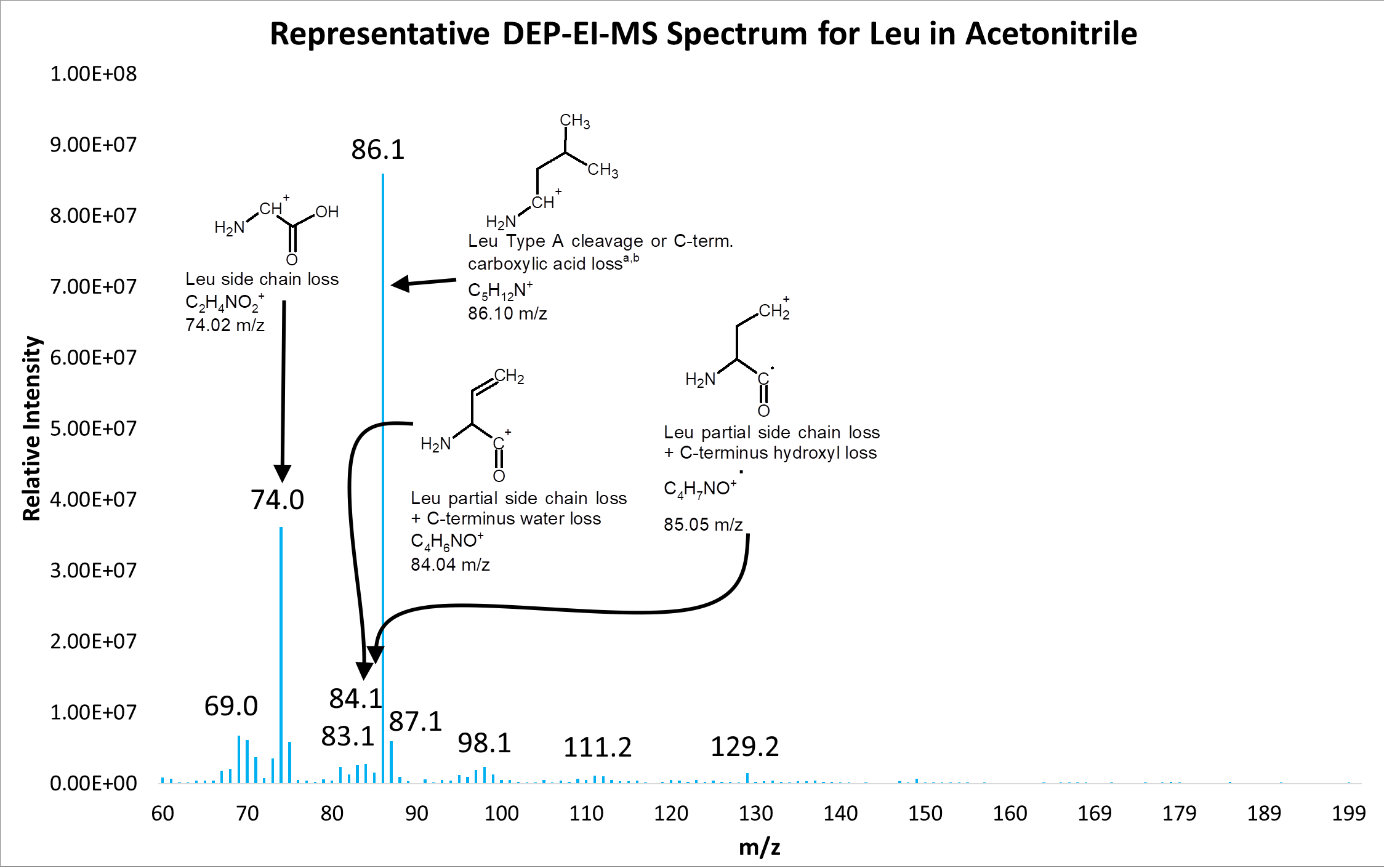

Supplement: S26 Fig — Several fragmentation mechanisms are present in the Leu spectrum. The common side chain loss mechanism is present along with a Type A-like cleavage resulting in the loss of the C-terminal carboxylic acid group. Peaks associated with partial loss of the side chain were observed in combination with loss of either water or a hydroxyl loss from the C-terminus. The maximum peak intensity for the shown spectrum is 8.59 X 107 counts. Proposed structures are shown along with the resulting fragment formula and monoisotopic m/z. adegradation type also observed in [11], bdegradation type also observed in [3]. (TIF) [file pone.0297752.s027.tif]

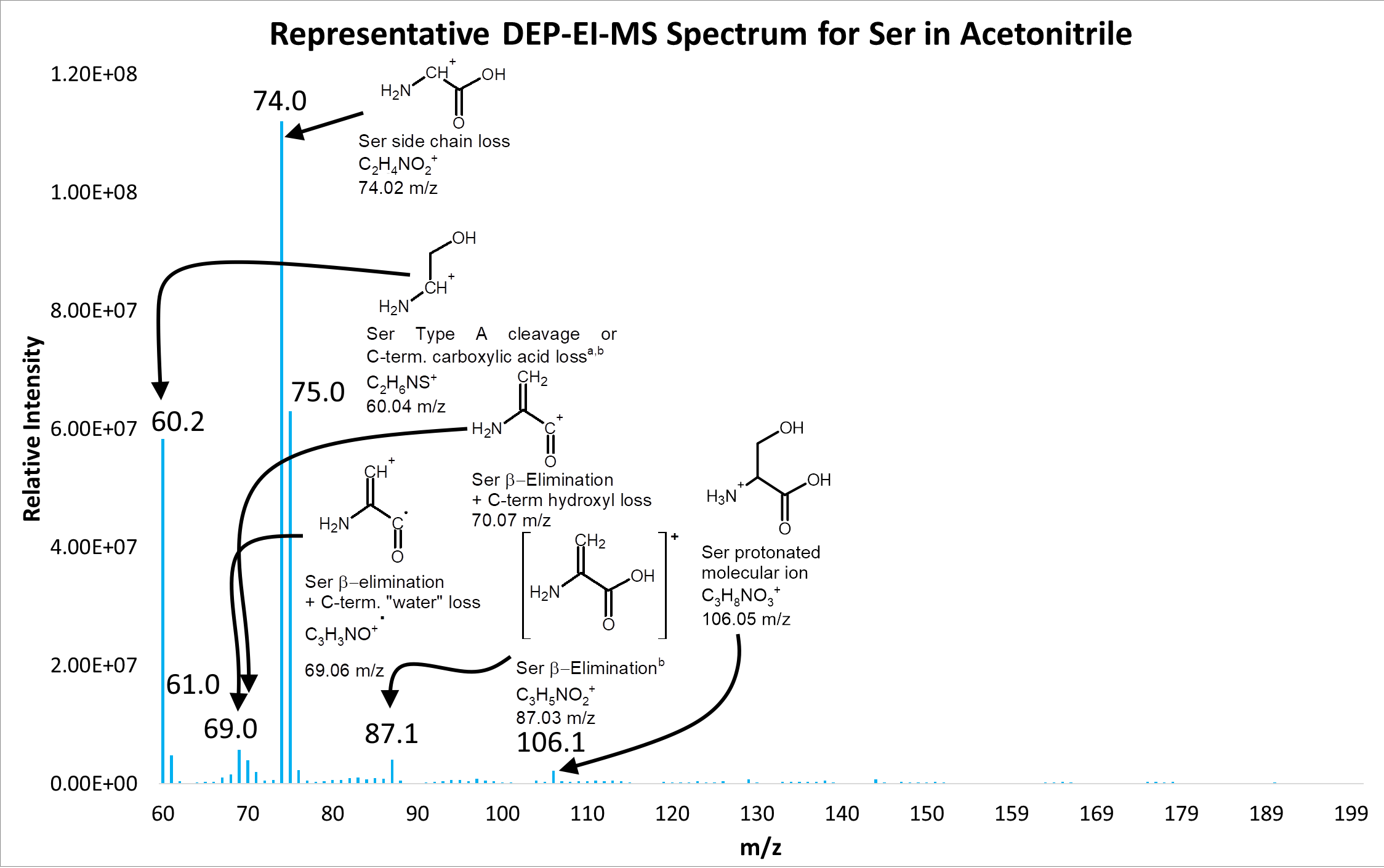

Supplement: S27 Fig — Several fragmentation mechanisms are present in the Ser spectrum. No non-relative peak fragmentation mechanisms were seen. The common full loss of side chain mechanism was observed. Peaks corresponding to β-elimination and C-terminal loss of water as well as β-elimination and loss of the C-terminal hydroxyl group are observed. No peak corresponding to β-elimination alone was observed. Type A-like cleavage or the loss of the C-terminal carboxyl group was observed. C-terminal loss of water was seen as well as the protonated molecular ion. The maximum peak intensity for the SerOH spectrum is 1.12 X 108 counts. Proposed structures are shown along with the resulting fragment formula and monoisotopic m/z. adegradation type also observed in [11], bdegradation type also observed in [3]. (TIF) [file pone.0297752.s028.tif]

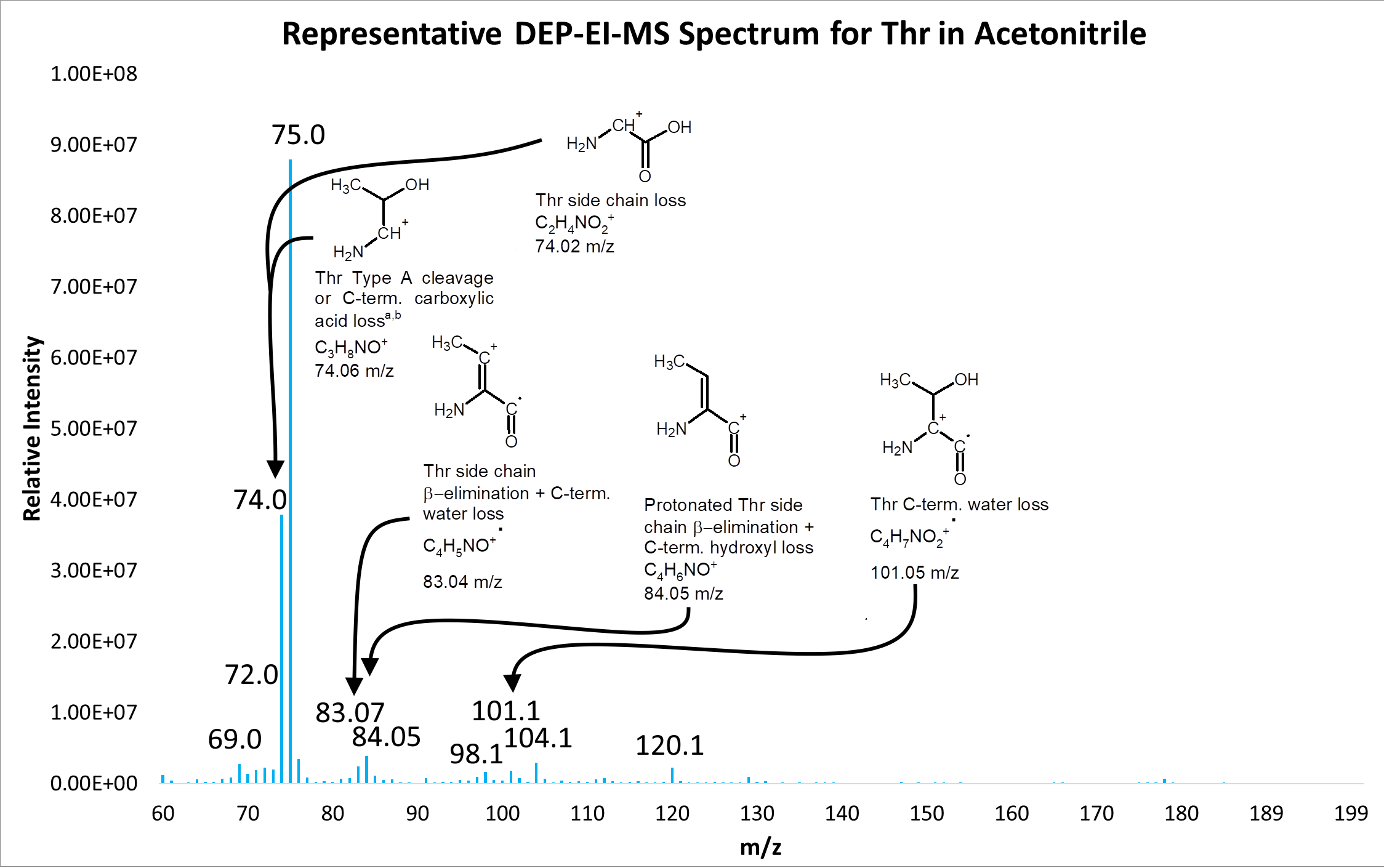

Supplement: S28 Fig — Several fragmentation mechanisms are present in the Thr spectrum. No non-relative peak fragmentation mechanisms were identified. The peak at a m/z of 74.0 could correspond either to the common side chain loss mechanism, or due to Type A-like cleavage or the loss of the C-terminal carboxylic acid group. Two β-elimination peaks combined with the loss of a C-termina water or hydroxyl group are observed. A peak corresponding to the loss of water from the C-terminus is also present. The maximum peak intensity for the shown spectrum is 8.79 X 107 counts. Proposed structures are shown along with the resulting fragment formula and monoisotopic m/z. adegradation type also observed in [11], bdegradation type also observed in [3]. (TIF) [file pone.0297752.s029.tif]

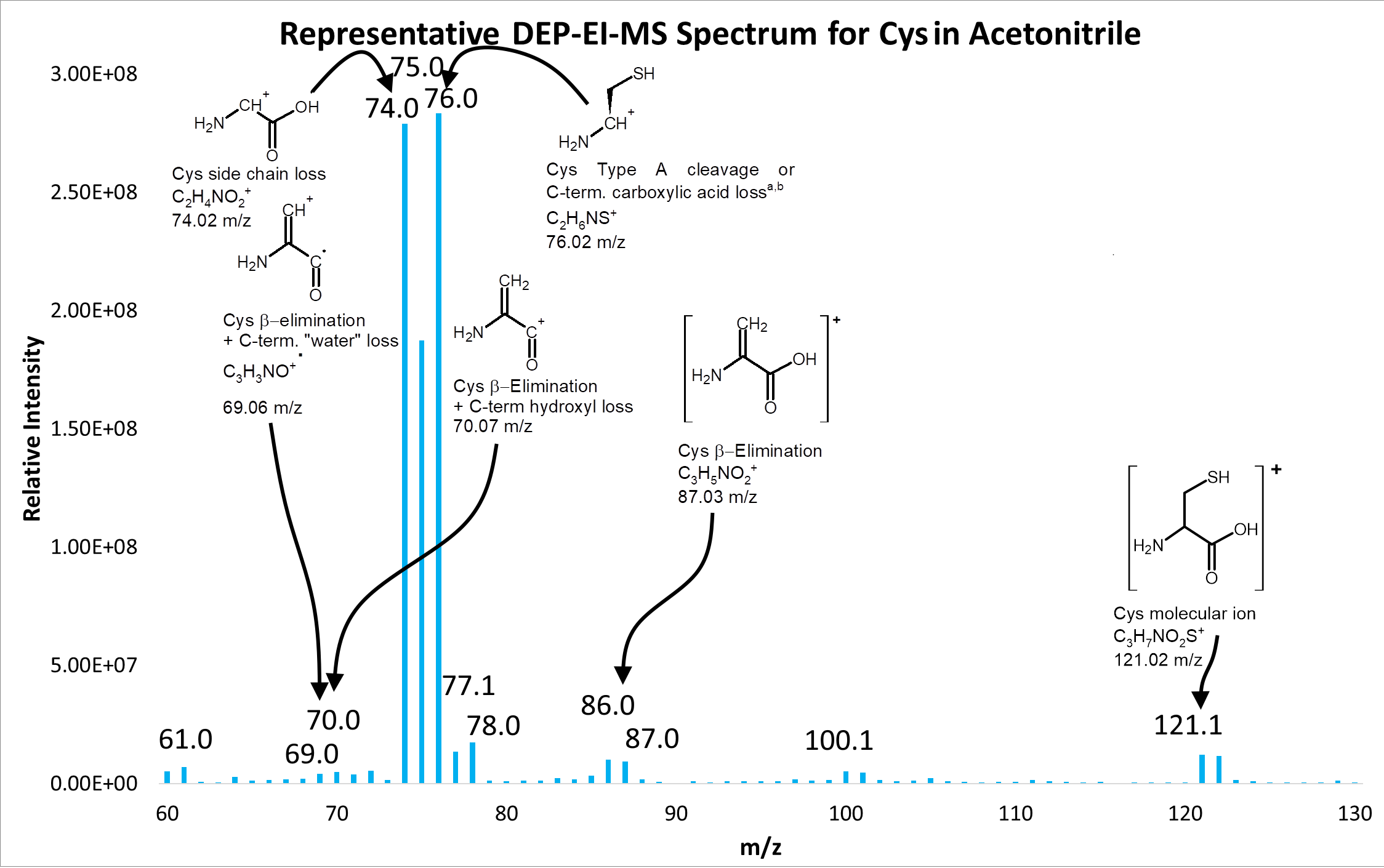

Supplement: S29 Fig — Several fragmentation mechanisms are present in the Cys spectrum. No non-relative fragmentation mechanisms were seen. The common full loss of the side chain mechanism was observed. Peaks corresponding to β-elimination alone, β-elimination and loss of the C-terminal hydroxyl group, and β-elimination and C-terminal loss of water were observed. Type A-like cleavage or the loss of the C-terminal carboxyl group was seen, as well as a peak corresponding to the molecular ion. The maximum peak intensity for Cys spectrum is 2.83 X 108 counts. Proposed structures are shown along with the resulting fragment formula and monoisotopic m/z. adegradation type also observed in [11], bdegradation type also observed in [3]. (TIF) [file pone.0297752.s030.tif]

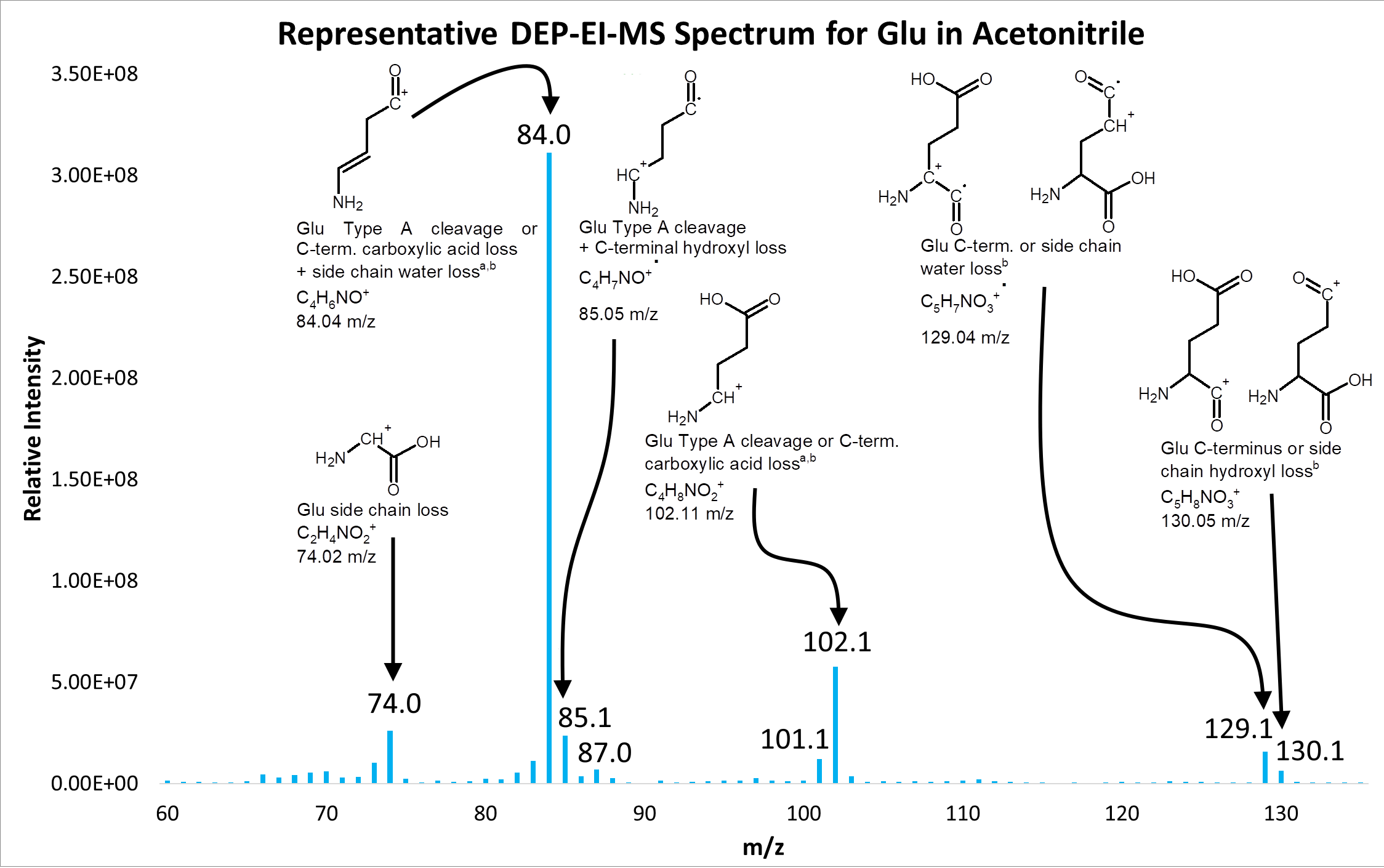

Supplement: S30 Fig — Several fragmentation mechanisms are present in the Glu spectrum. No non-relative peak fragmentation mechanisms were seen. The common full loss of side chain mechanism was observed. Peaks corresponding to Type A-like cleavage or the loss of the C-terminal carboxylic acid group and side chain water loss, Type A-like cleavage and loss of the C-terminal hydroxyl group, and Type A-like cleavage or C-terminal carboxylic acid loss were seen. A peak corresponding to either C-terminal or side chain water loss was observed. A peak corresponding to either C-terminal or side chain hydroxyl group loss was observed. The maximum peak intensity for the shown spectrum is 3.11 X 108 counts. Proposed structures are shown along with the resulting fragment formula and monoisotopic m/z. adegradation type also observed in [11], bdegradation type also observed in [3]. (TIF) [file pone.0297752.s031.tif]

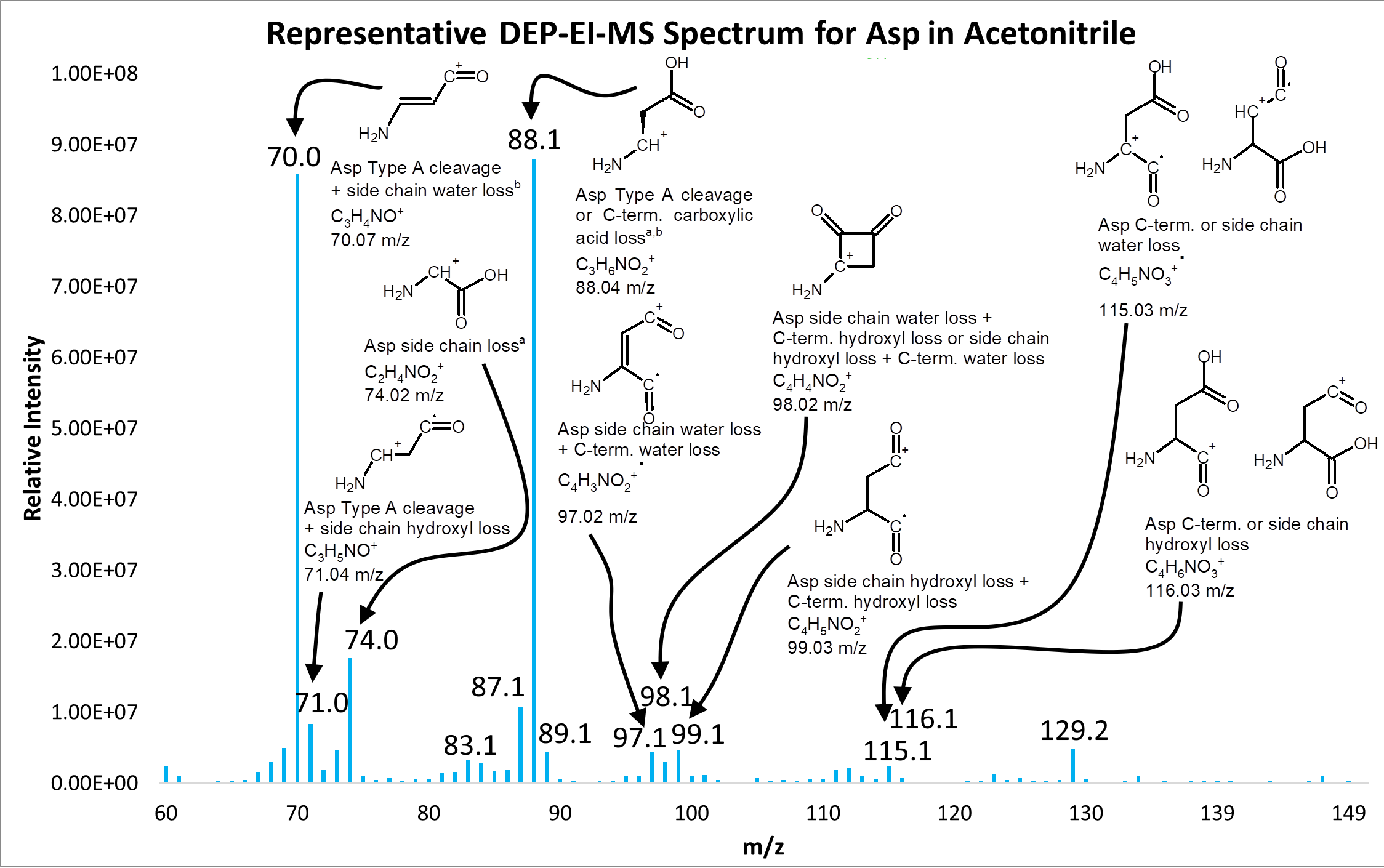

Supplement: S31 Fig — Several fragmentation mechanisms are present in the Asp spectrum. No non-relative peak fragmentation mechanisms were seen. The common full loss of side chain mechanism was observed. Peaks corresponding to Type A-like cleavage and side chain water loss, Type A-like cleavage and loss of the side chain hydroxyl group, and Type A-like cleavage or C-terminal carboxylic acid loss were seen. Peaks corresponding to side chain water loss and C-terminal water loss, and side chain hydroxyl loss and C-terminal hydroxyl loss were observed. A peak corresponding to either side chain water loss and C-terminal hydroxyl loss, or side chain hydroxyl loss and C-terminal water loss was seen. A peak corresponding to either C-terminal or side chain water loss was observed. A peak corresponding to either C-terminal or side chain hydroxyl group loss was observed. The maximum peak intensity for the shown spectrum is 8.80 X 107 counts. Proposed structures are shown along with the resulting fragment formula and monoisotopic m/z. adegradation type also observed in [11], bdegradation type also observed in [3]. (TIF) [file pone.0297752.s032.tif]

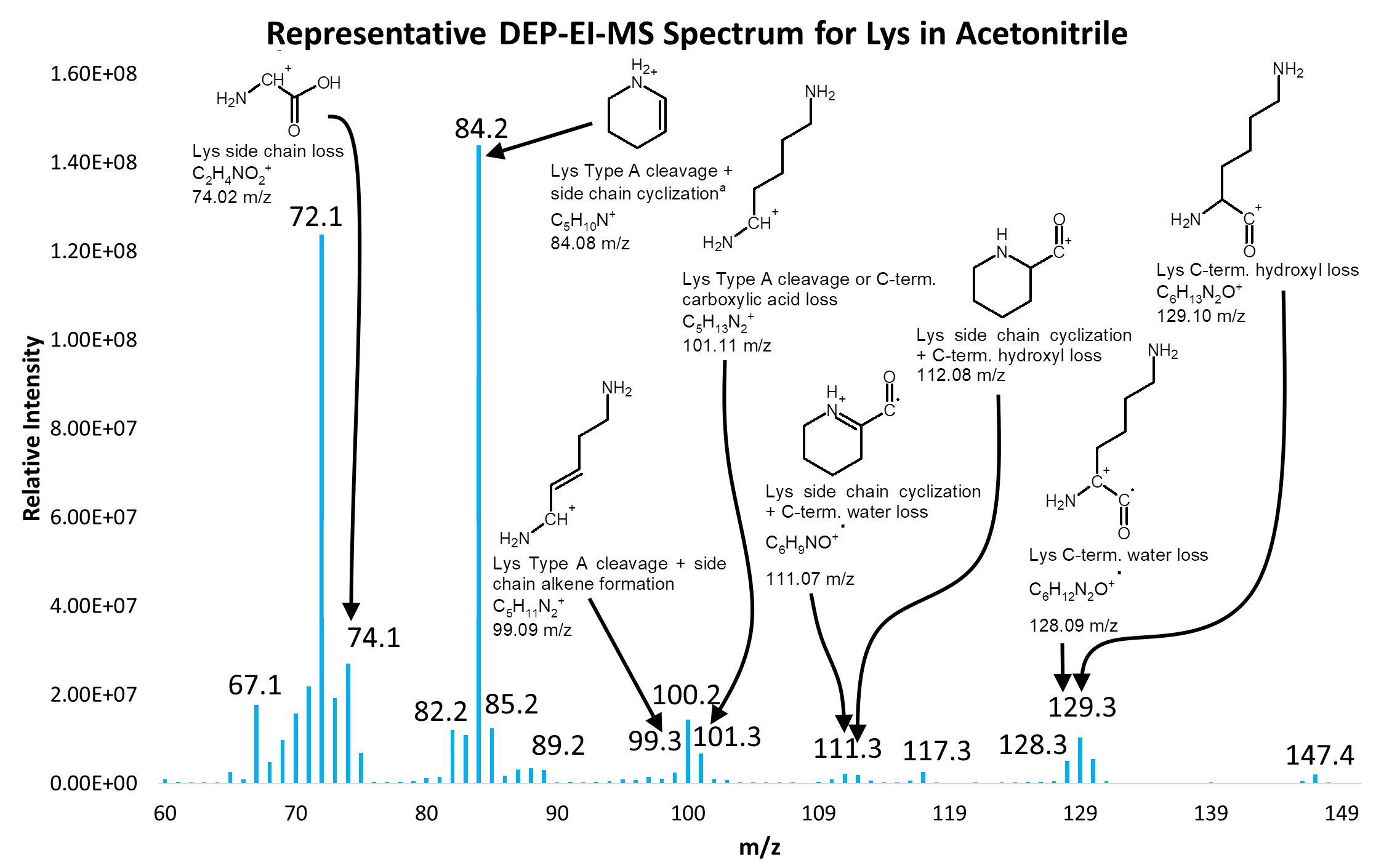

Supplement: S32 Fig — Several fragmentation mechanisms are present in the Lys spectrum. No non-relative peak fragmentation mechanisms were seen. The common full loss of side chain mechanism was observed. Peaks corresponding to Type A-like cleavage and side chain cyclization, Type A-like cleavage and side chain alkene formation, and Type A-like cleavage or C-terminal carboxylic acid loss were seen. Peaks corresponding to side chain cyclization and C-terminal hydroxyl loss, and side chain cyclization and C-terminal water loss were observed. A peak indicating C-terminal hydroxyl loss was seen, as well as a peak indicating C-terminal water loss. The maximum peak intensity for the shown spectrum is 1.44 X 108 counts. Proposed structures are shown along with the resulting fragment formula and monoisotopic m/z. adegradation type also observed in [11], bdegradation type also observed in [3]. (TIF) [file pone.0297752.s033.tif]

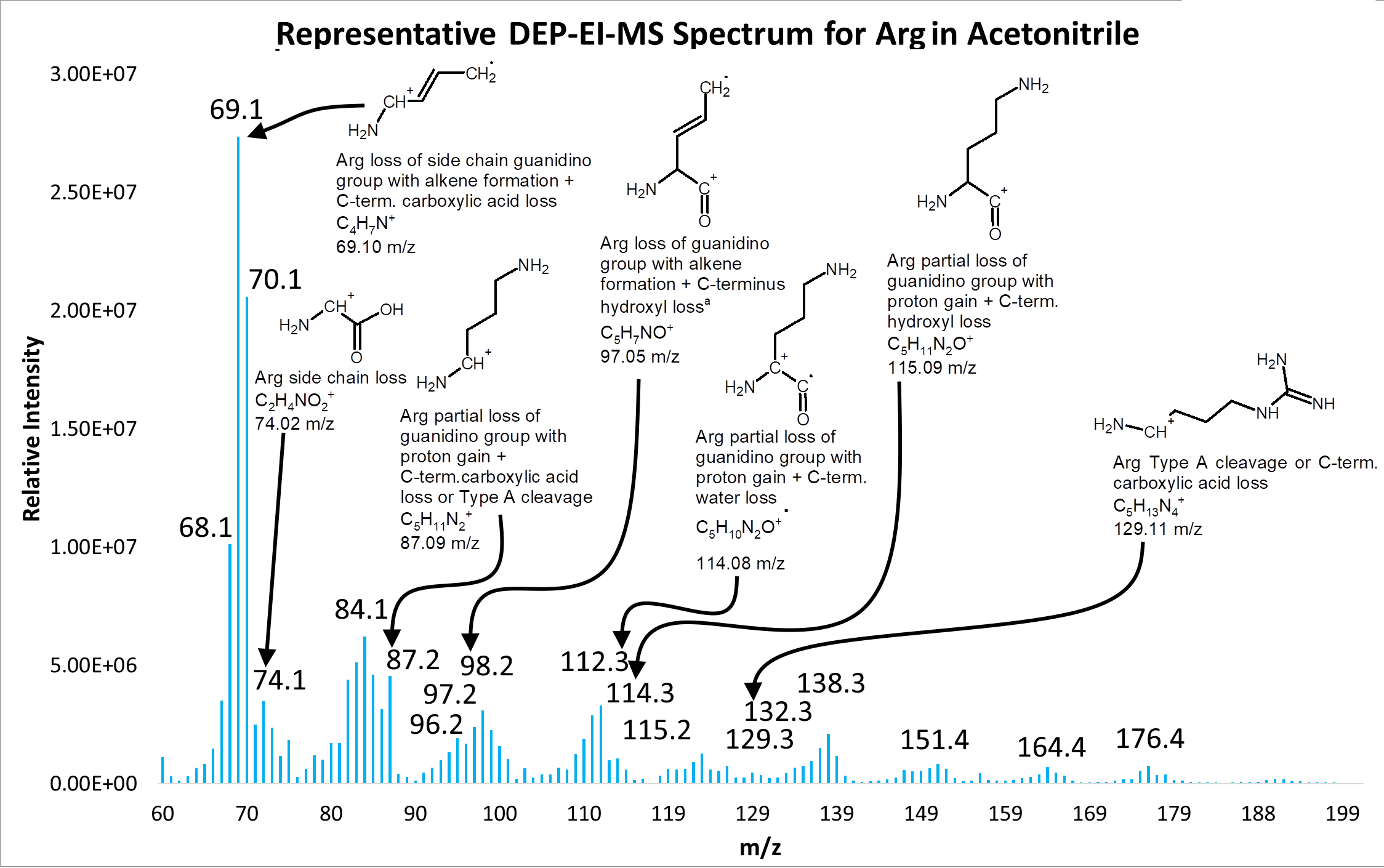

Supplement: S33 Fig — Several fragmentation mechanisms are present in the Arg spectrum. No non-relative peak fragmentation mechanisms were seen. Loss of side chain guanidino group with alkene formation and C-terminal carboxylic acid loss was seen. Peaks corresponding to partial loss of guanidino group with proton gain and C-terminal water loss, as well as partial loss of guanidino group with proton gain and C-terminal carboxylic acid loss or Type A-like cleavage, were observed. A peak corresponding to Type A-like cleavage of C-terminal carboxylic acid loss was seen. The common full loss of side chain mechanism was observed. Loss of the guanidino group with alkene formation and C-terminal hydroxyl loss was noted. Partial loss of the guanidino group with proton gain and C-terminal hydroxyl loss was also observed. The maximum peak intensity for the shown spectrum is 2.73 X 107 counts. Apparent structures are shown along with the resulting fragment formula and monoisotopic m/z. adegradation type also observed in [11], bdegradation type also observed in [3]. (TIF) [file pone.0297752.s034.tif]

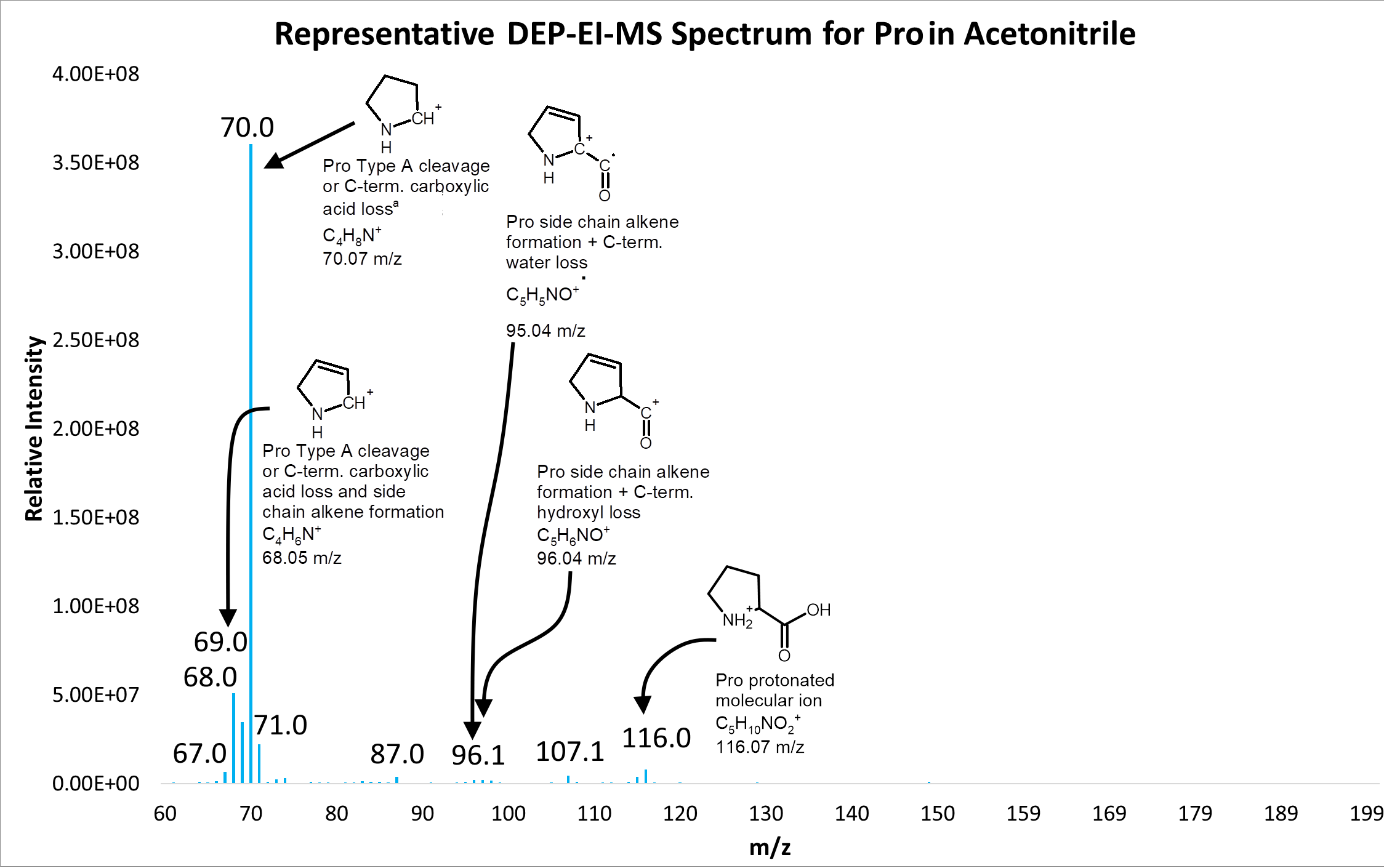

Supplement: S34 Fig — Several fragmentation mechanisms are present in the Pro spectrum. No non-relative peak fragmentation mechanisms were seen. Peaks corresponding to Type A-like cleavage or C-terminal carboxylic acid loss, and Type A-like cleavage or C-terminal carboxylic acid loss and side chain alkene formation were observed. Peaks corresponding to side chain alkene formation and C-terminal water loss, and side chain alkene formation and C-terminal hydroxyl loss were observed. The protonated molecular ion was also observed. The maximum peak intensity for the shown spectrum is 3.60 X 108 counts. Proposed structures are shown along with the resulting fragment formula and monoisotopic m/z. adegradation type also observed in [11], bdegradation type also observed in [3]. (TIF) [file pone.0297752.s035.tif]

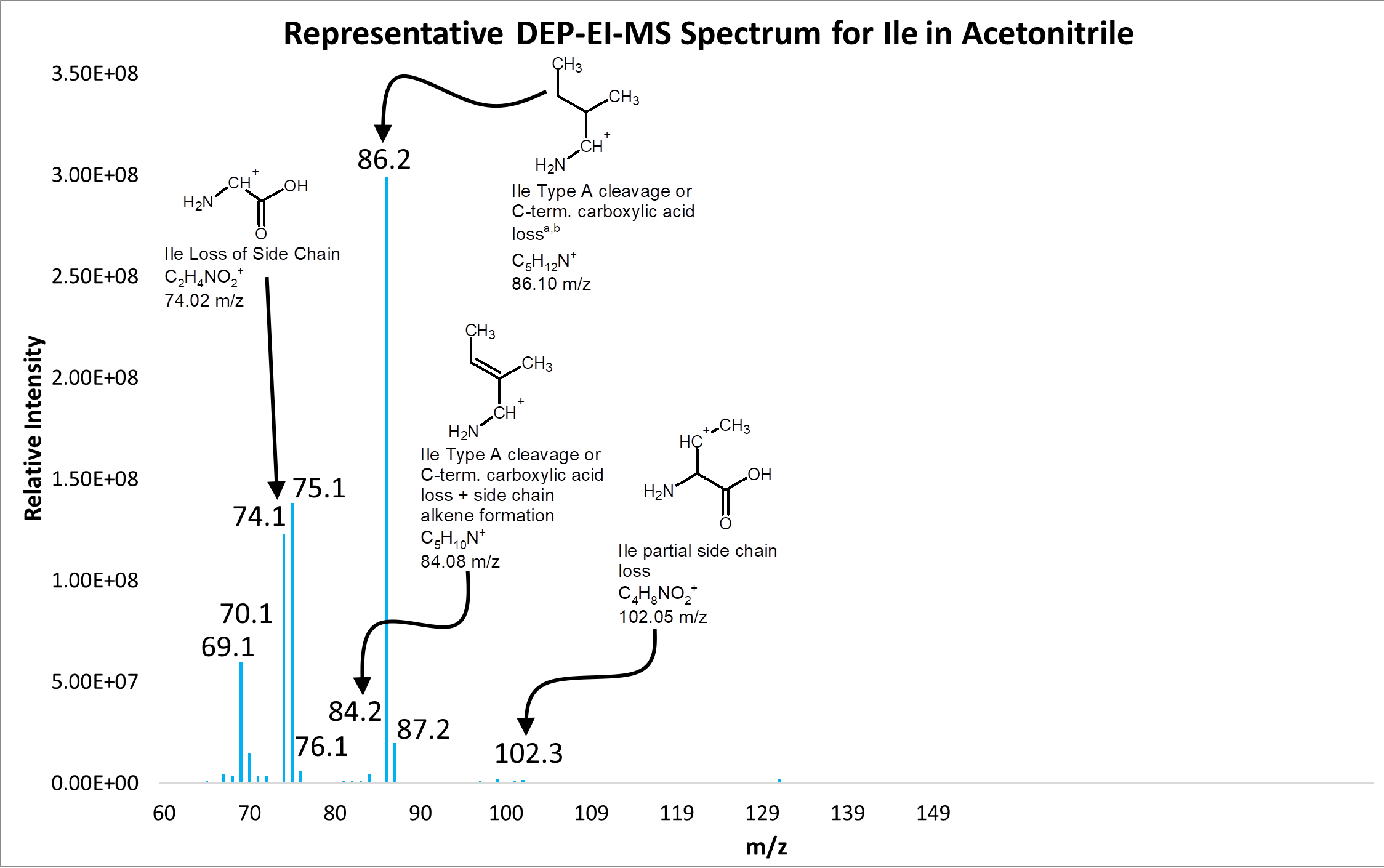

Supplement: S35 Fig — Several fragmentation mechanisms are present in the Ile spectrum. No non-relative peak fragmentation mechanisms were seen. Peaks corresponding to side chain loss and partial side chain loss were seen. Peaks corresponding to Type A-like cleavage or C-terminal carboxylic acid loss and side chain alkene formation, and Type A-like cleavage or C-terminal carboxylic acid loss were also observed. The maximum peak intensity for the shown spectrum is 2.99 X 108 counts. Proposed structures are shown along with the resulting fragment formula and monoisotopic m/z. adegradation type also observed in [11], bdegradation type also observed in [3]. (TIF) [file pone.0297752.s036.tif]

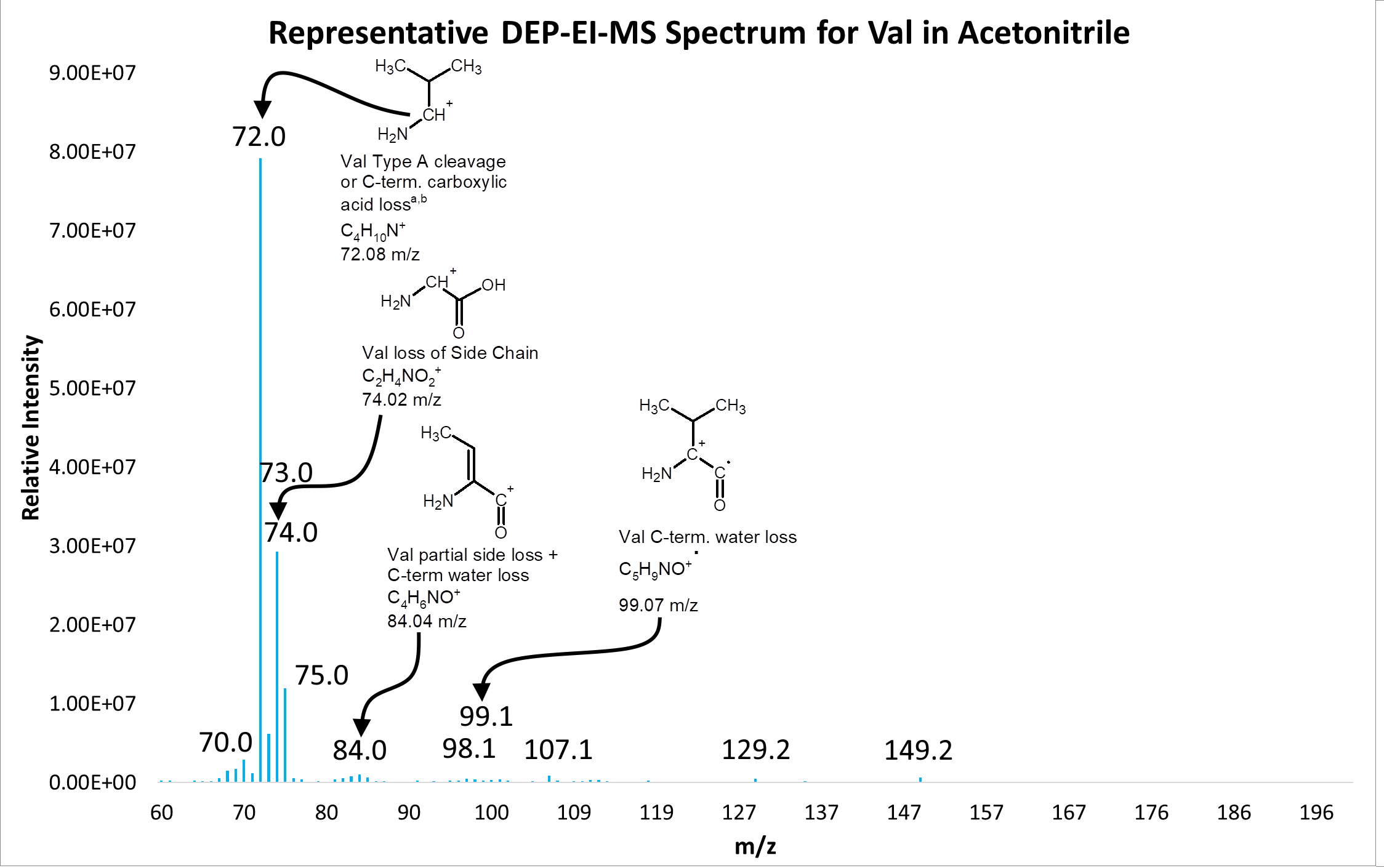

Supplement: S36 Fig — Several fragmentation mechanisms are present in the Val spectrum. No non-relative peak fragmentation mechanisms were seen. Type A-like cleavage or C-terminal carboxylic acid loss was observed. Peaks corresponding to side chain loss, and partial side chain loss and C-terminal water loss were seen. C-terminal water loss was also observed. The maximum peak intensity for the shown spectrum is 7.92 X 107 counts. Proposed structures are shown along with the resulting fragment formula and monoisotopic m/z. adegradation type also observed in [11], bdegradation type also observed in [3]. (TIF) [file pone.0297752.s037.tif]
