# Supplementary material for: Development of a Python-based electron ionization mass spectrometry amino acid and peptide fragment prediction model
Source: PLoS One. 2024 Feb 16;19(2):e0297752. doi: 10.1371/journal.pone.0297752 (PMC10871511; doi:10.1371/journal.pone.0297752)
Supplement: S1 File — Representative spectra for the proteinogenic amino acids alanine and glycine are included along with spectra corresponding to the Trt, Fmoc, Pbf, and Boc/t-Bu protecting groups. (PDF) [file pone.0297752.s038.pdf]

Representative DEP-EI-MS Spectra of AlaOH and GlyOH

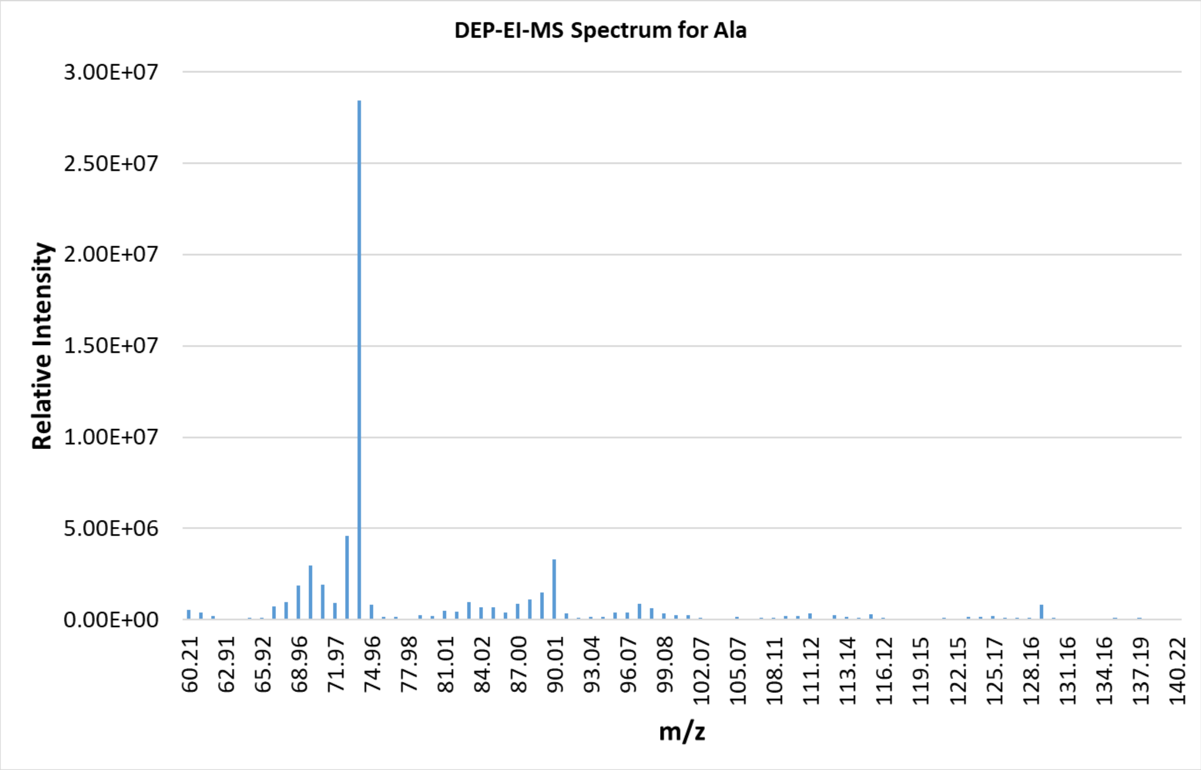

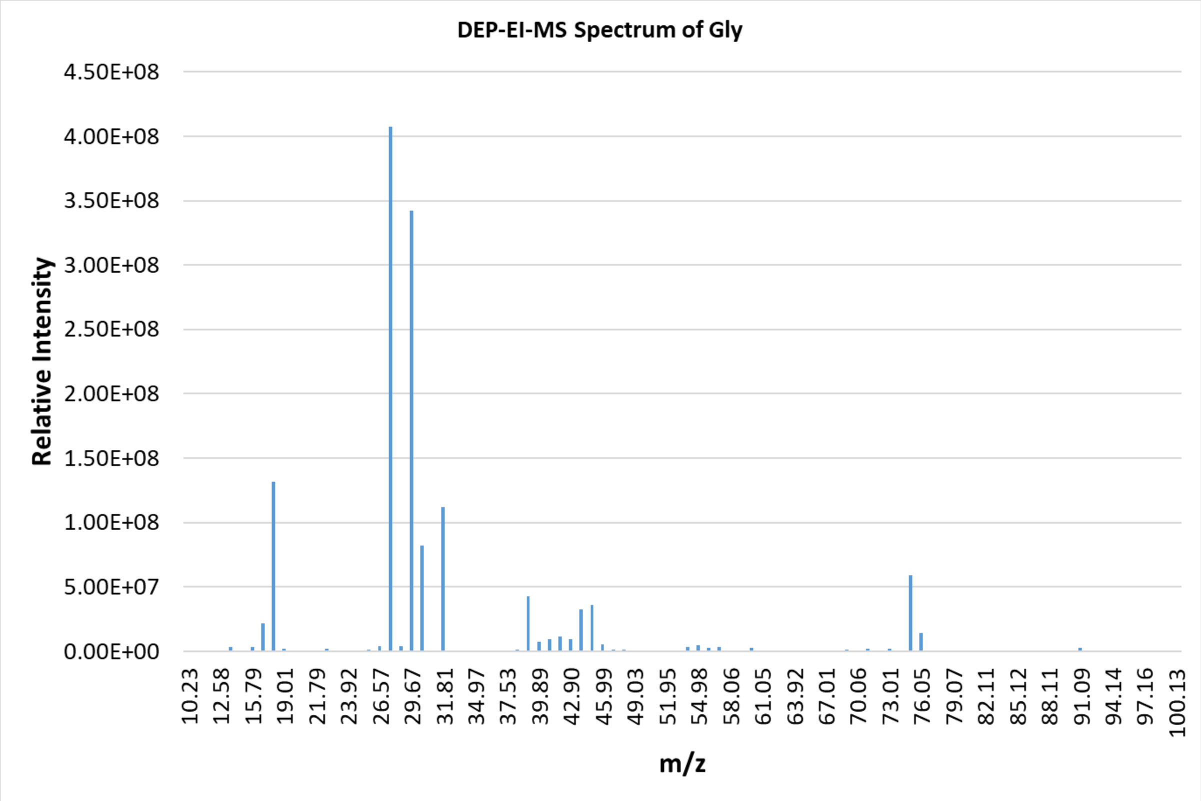

Representative DEP-EI-MS Spectra of Synthesis Protecting Groups

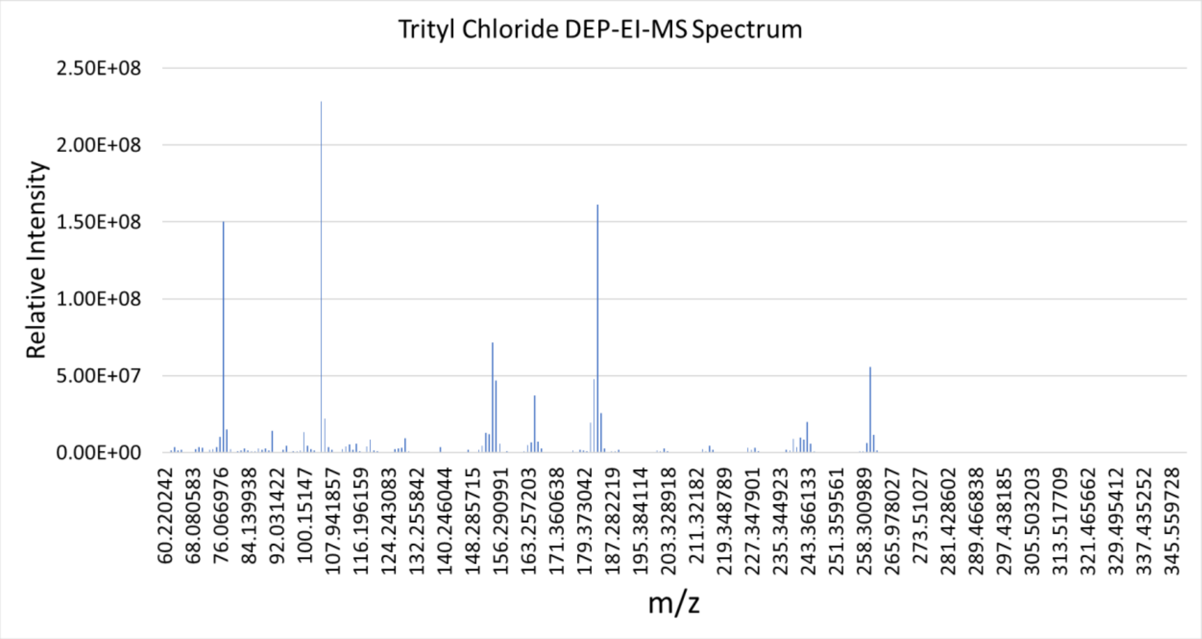

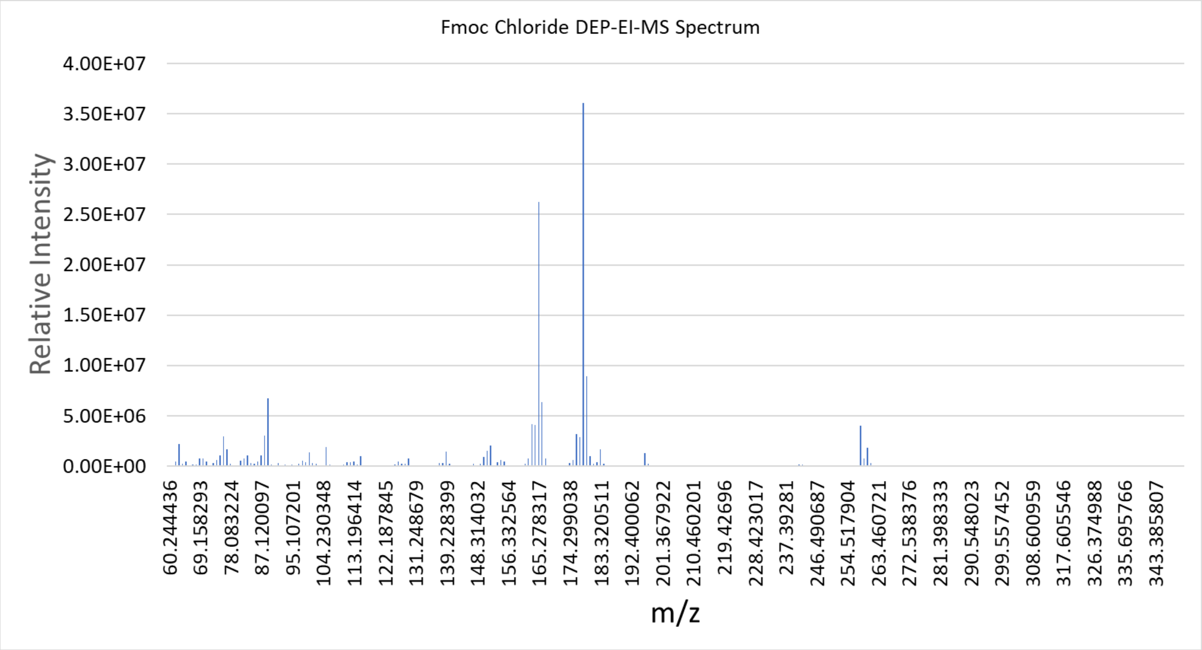

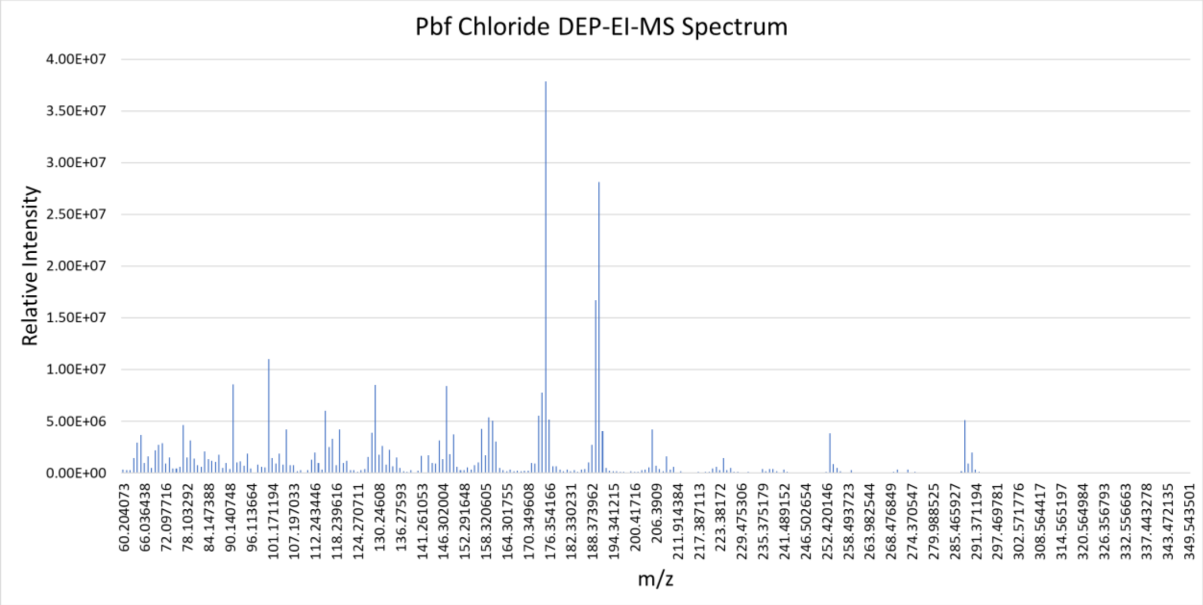
Pbf Chloride DEP-EI-MS Spectrum
Relative Intensity
4.00E+07
3.50E+07
3.00E+07
2.50E+07
2.00E+07
1.50E+07
1.00E+07
5.00E+06
0.00E+00
m/z

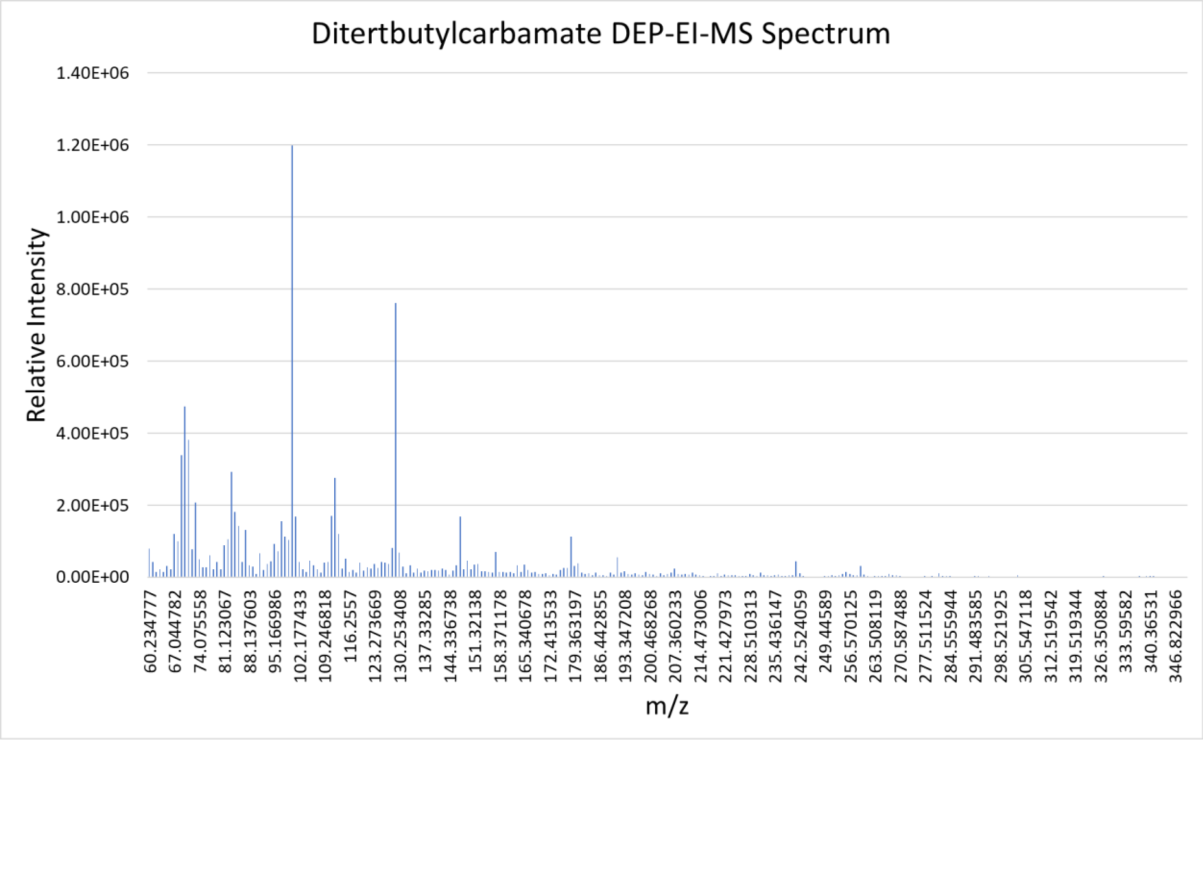
Ditertbutylcarbamate DEP-EI-MS Spectrum
Relative Intensity
1.40E+06
1.20E+06
1.00E+06
8.00E+05
6.00E+05
4.00E+05
2.00E+05
0.00E+00
m/z
