# Supplementary material for: Development of a Python-based electron ionization mass spectrometry amino acid and peptide fragment prediction model
Source: PLoS One. 2024 Feb 16;19(2):e0297752. doi: 10.1371/journal.pone.0297752 (PMC10871511; doi:10.1371/journal.pone.0297752)
Supplement: S2 File — The provided link allows access to the public Github repository with the source Python script file as well as the used definition files. (PDF) [file pone.0297752.s039.pdf]

Link to Github Source Code Repository

https://github.com/mcbrayed/EIMS-Peptide-Fragment-Predictor
